# Climate change may shift metapopulations towards unstable source‐sink dynamics in a fire‐killed, serotinous shrub

**DOI:** 10.1002/ece3.11488

**Published:** 2024-06-03

**Authors:** Rodrigo Souto‐Veiga, Juergen Groeneveld, Neal J. Enright, Joseph B. Fontaine, Florian Jeltsch

**Affiliations:** ^1^ Department of Plant Ecology and Nature Conservation University of Potsdam Potsdam Germany; ^2^ School of Environmental and Conservation Sciences Murdoch University Murdoch Western Australia Australia; ^3^ Institute of Plant Science and Microbiology, Ecological Modeling Universität Hamburg Hamburg Germany; ^4^ Department of Ecological Modelling Helmholtz Centre for Environmental Research – UFZ Leipzig Germany; ^5^ Berlin‐Brandenburg Institute of Advanced Biodiversity Research (BBIB) Berlin Germany

**Keywords:** interval squeeze, Mediterranean‐type ecosystem, process‐based simulation model, source‐sink dynamics

## Abstract

Climate change, with warming and drying weather conditions, is reducing the growth, seed production, and survival of fire‐adapted plants in fire‐prone regions such as Mediterranean‐type ecosystems. These effects of climate change on local plant demographics have recently been shown to reduce the persistence time of local populations of the fire‐killed shrub *Banksia hookeriana* dramatically. In principle, extinctions of local populations may be partly compensated by recolonization events through long‐distance dispersal mechanisms of seeds, such as post‐fire wind and bird‐mediated dispersal, facilitating persistence in spatially structured metapopulations. However, to what degree and under which assumptions metapopulation dynamics might compensate for the drastically increased local extinction risk remains to be explored. Given the long timespans involved and the complexity of interwoven local and regional processes, mechanistic, process‐based models are one of the most suitable approaches to systematically explore the potential role of metapopulation dynamics and its underlying ecological assumptions for fire‐prone ecosystems. Here we extend a recent mechanistic, process‐based, spatially implicit population model for the well‐studied fire‐killed and serotinous shrub species *B. hookeriana* to a spatially explicit metapopulation model. We systematically tested the effects of different ecological processes and assumptions on metapopulation dynamics under past (1988–2002) and current (2003–2017) climatic conditions, including (i) effects of different spatio‐temporal fires, (ii) effects of (likely) reduced intraspecific plant competition under current conditions and (iii) effects of variation in plant performance among and within patches. In general, metapopulation dynamics had the potential to increase the overall regional persistence of *B. hookeriana*. However, increased population persistence only occurred under specific optimistic assumptions. In both climate scenarios, the highest persistence occurred with larger fires and intermediate to long inter‐fire intervals. The assumption of lower intraspecific plant competition caused by lower densities under current conditions alone was not sufficient to increase persistence significantly. To achieve long‐term persistence (defined as >400 years) it was necessary to additionally consider empirically observed variation in plant performance among and within patches, that is, improved habitat quality in some large habitat patches (≥7) that could function as source patches and a higher survival rate and seed production for a subset of plants, specifically the top 25% of flower producers based on current climate conditions monitoring data. Our model results demonstrate that the impacts of ongoing climate change on plant demographics are so severe that even under optimistic assumptions, the existing metapopulation dynamics shift to an unstable source‐sink dynamic state. Based on our findings, we recommend increased research efforts to understand the consequences of intraspecific trait variation on plant demographics, emphasizing the variation of individual traits both among and within populations. From a conservation perspective, we encourage fire and land managers to revise their prescribed fire plans, which are typically short interval, small fires, as they conflict with the ecologically appropriate spatio‐temporal fire regime for *B. hookeriana*, and likely as well for many other fire‐killed species.

## Graphical abstract

We extend a recent mechanistic, process‐based, spatially implicit population model for the well‐studied fire‐killed and serotinous shrub species to a spatially explicit metapopulation model. Our model results demonstrate that the impacts of ongoing climate change on plant demographics are so severe that even under optimistic assumptions, the existing metapopulation dynamics shift to an unstable source‐sink dynamic state.

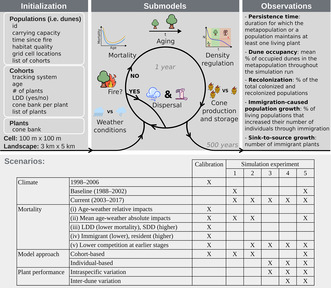

## 1 INTRODUCTION

Climate change is substantially shifting the environmental conditions for plant populations in fire‐prone Mediterranean Type Ecosystems globally (Flannigan et al., 2009; Lozano et al., 2017). For example, in South‐West Australia since the mid‐1970s rainfall has decreased by >15% (Timbal et al., 2006), mean temperatures have increased approx. 1.5°C (Grainger et al., 2022) and hot days >40°C and heat waves have doubled in frequency (Breshears et al., 2021). Climate changes have also altered the fire regime, increasing fire danger, intensity and frequency (Valente & Laurini, 2021) which in turn may change community assembly in these fire‐prone plant communities (Mouillot et al., 2002).

Enright et al. (2015) conceptualized the impact of climate change induced effects (e.g. less precipitation and shorter fire return intervals) on plant demographics in their theory of ‘interval squeeze’. This theoretical framework suggests that the combination of reduced seed production (demographic shift), reduced seedling establishment after fire (post‐fire recruitment shift) and reduction in the time between successive fires (fire interval shift) will cumulatively threaten the persistence of species populations under climate change. Recent simulation models confirmed that indeed the persistence time on local populations of serotinous plant species has decreased based on recent data on fire intervals, seedling survival and precipitation (Henzler et al., 2018) with reduced pollination as an additional accelerating factor of decline (Souto‐Veiga et al., 2022).

However, this pessimistic view remains incomplete since it has been shown that long‐distance dispersal (LDD) may connect local populations to form a metapopulation in which local extinctions may be prevented or compensated by recolonization events (Groeneveld et al., 2008; He et al., 2004, 2010). Though metapopulation dynamics are often difficult to verify in plants because of dormant stages and persistent seed banks, there are a number of examples in the literature including for annuals (Dornier et al., 2011), long‐lived herbs (Bullock et al., 2020) and fire‐prone serotinous perennial shrubs (e.g. *Banksia hookeriana*; Groeneveld et al., 2008). Local extinctions in such systems are often caused by disturbances that vary substantially in size, from small‐scale disturbances such as gap creation by grazing animals (Bullock et al., 2020), up to landscape‐scale events (e.g. fire; Groeneveld et al., 2008). In serotinous plants (seeds held in woody fruits in the plant's canopy) the spatial extent of the disturbance is of particular importance since fire can not only cause correlated local extinctions of several sub‐populations, but fire is also a necessary prerequisite for seed release, dispersal and germination—and so, potential recolonization of lost habitat.

New environmental conditions may also decrease intraspecific competition for water in plants indirectly: At earlier life stages survival rates may be lower due to decreased precipitation and available soil water which lead to an overall lower density of adult plants. This lower overall abundance of plants may decrease intraspecific competition for water and increase survival.

In general, the positive effect of spatially structured metapopulation dynamics depends on recolonization events that facilitate regional survival in spite of local population extinction (Hanski, 1998). Recolonization events require both local population dynamics that provide a sufficient number of seeds and their dispersal to other subpopulations. Although it has been shown that LDD is possible for our study species *B. hookeriana* (He et al., 2004, 2010), it is less clear whether such LDD events can indeed drive metapopulation dynamics and how this depends on the performance of local populations and specific environmental scenarios. New environmental conditions, for example, may cause a complete breakdown of regional dynamics by reducing local seed production and interrupting effective exchange of viable seeds between subpopulations. By reducing the persistence of local populations or their capacity to act as a colonization source, climate change may also transform plant metapopulations into source‐sink systems where long‐distance dispersed seeds are lost rather than serving as an essential element of metapopulation persistence.

Using the well‐studied fire‐killed and serotinous shrub *B. hookeriana* as a model species, we aim to better understand the potential role of metapopulation dynamics for mitigating the negative consequences of climatic changes for species in fire‐prone landscapes. We extend a recent mechanistic, process‐based, spatially implicit population model of *B. hookeriana* (Souto‐Veiga et al., 2022) to a spatially explicit metapopulation model. We systematically test the effects of different ecological processes and assumptions on metapopulation dynamics under past (i.e. baseline 1988–2002) and current (2003–2017) climatic conditions. Through a series of five simulation experiments, we provide a layered understanding of metapopulation dynamics under diverse ecological conditions. Starting with the broad impacts of different fire regimes (size and frequency) under both climate scenarios (Experiment 1), we then focus on the current climate scenario to identify processes and assumptions that enhance persistence today (and likely in the prospective future), exploring effects of lower intraspecific plant competition caused by reduced densities (Experiment 2), observed intraspecific variation in plant performance (Experiment 3) and the combined influence of intraspecific and inter‐patch variation in plant performance (Experiment 4). The culmination of these experiments is the comparison of metapopulation dynamics—recolonization and immigration‐caused population growth events, sink‐to‐source growth and the persistence of source and sink populations—between baseline and current climate conditions. Specifically, under current conditions, we apply an optimized scenario of intraspecific and habitat patch variation, as determined in Experiment 4, to achieve long‐term persistence (Experiment 5). However, this final experiment reveals that even under this optimized scenario, designed to enhance population survival, we observe a shift from traditional metapopulation dynamics, where each patch has the potential to support a viable population, to unstable source‐sink dynamics, characterized by a dependency on a few high‐quality patches for overall population support. This transition underscores the profound impact of climate change on plant demographics and highlights the urgent need for conservation strategies that are adaptive to the complexities of fire‐prone environments, aiming to secure the persistence of *B. hookeriana* and similar species.

## 2 METHODS

### 2.1 Study site and study species

*Banksia hookeriana* (Proteaceae) is a local endemic, fire‐killed serotinous shrub, confined to the Eneabba Plain in Western Australia; a region spanning 30 km by 80 km about 250–330 km north of Perth (Miller et al., 2007; Taylor & Hopper, 1988). It is a dominant structural component of shrublands in this region, primarily due to its size (up to 2.5 m in height), longevity (up to 40 years), adaptation to recurrent fires and its seed‐based regeneration mechanism. The species' lifecycle and distribution have been heavily influenced by human activities, including land clearing for agriculture and mining, and commercial wildflower picking, leading to a 40% reduction in its geographical range since 1960 (He et al., 2010; Lamont et al., 2001; Witkowski et al., 1994). *Banksia hookeriana* exists in at least part of its range as a metapopulation scattered across undulating dune fields, with individual populations present on dune crests (deep, acid sands) geographically separated by uninhabitable intervening dune swales (shallow sands) 0.1 to >1 km wide. These dune fields are aeolian in origin and were last active around the last glacial maximum approx. 20,000 ybp (Krauss et al., 2006). The study area of 3 km × 5 km used here was located at Beekeepers Nature Reserve, 10 km north of Eneabba, Western Australia (He et al., 2010). The fire return interval is estimated to be approx. 20 years, with most intervals in the range 15–25 years for both planned and unplanned fires so that stands rarely exceed 30 years since the last fire (Enright et al., 2012). Fire stimulates the general release of predominantly outcrossed seeds (Barrett et al., 2005), which are held in closed woody fruits in the plant crown for up to 12 years in a state of forced dormancy (serotiny) (Lamont et al., 1991).

In the aftermath of fires, *B. hookeriana* seeds are dispersed through two key mechanisms: wind and bird‐mediated dispersal. Initially, seed dispersal occurs through gravity, with seeds dropping close to the parent plant. However, wind plays a crucial role in subsequent short and LDD. In terms of short‐distance dispersal (SDD), seeds may be blown by wind along the ground for a few meters until they encounter a barrier to further movement such as fallen branches or litter‐filled micro‐depressions (Lamont et al., 1993). LDD of seeds both from open cones on burned plants as well as from seeds on the ground, may be facilitated by wind vortices, which are more common after fires due to the stronger heating of the largely bare soil surface (relative to unburned vegetation cover) over the hot summer months when fires are most likely to occur (He et al., 2004; Keith et al., 2020). Such wind vortices may carry seeds up to several kilometres away from their source (He et al., 2004). The removal of vegetation barriers by fire also aids in this wind‐assisted dispersal. In addition to local deposition, this dual mechanism of gravity and wind dispersal allows seeds to reach a variety of locations, including both immediate fire‐affected areas and regions further away (He et al., 2004).

A second important potential mechanism of LDD involves the Carnaby's black cockatoo (*Calyptorhynchus latirostris*). Following fires, these birds are attracted to *B. hookeriana* due to the increased accessibility of seeds. The heat from the fires causes a rupture in the protective woody follicles on cones, and removes foliage, making the seeds readily available for the birds (He et al., 2004; He & Lamont, 2010). While birds often remove the cones and fly to feeding sites beyond the immediate fire‐affected vicinity, it is possible they occasionally drop these cones en route. This could challenge the concept of directed dispersal. However, in the context of our study, we adopted an optimistic perspective and assumed that all seeds successfully reached suitable *B. hookeriana* habitat patches.

Crucially, *B. hookeriana* does not maintain a soil‐stored seed bank, and seeds must germinate during the next rainy season, otherwise they perish (Enright & Lamont, 1989).

### 2.2 Model description

We developed a model to investigate the role of metapopulation dynamics in mitigating the adverse effects of climate change on fire‐prone species. The model is an extension of the previously published population model of Souto‐Veiga et al. (2022). It includes post‐fire seed dispersal by wind, cone dispersal by birds (cockatoos), patchy fires, and variation in plant performance among and within populations as the main new submodels. A summary description of the model is provided below. The TRACE document (Grimm et al., 2014; Schmolke et al., 2010) in the supporting information (Appendix B) contains a detailed model description following the ODD (Overview, Design concepts, Details) protocol (Grimm et al., 2006, 2010, 2020). The model was implemented in C++ 17, and data analysis and visualization were performed using R v.3.5.3 (R Core Team, 2019). We used the following R packages: ‘ggplot2’ v3.3.6 (Wickham, 2016) for generating 2D figures and ‘plotly’ v4.10.0 (Sievert, 2020) for 3D figures; ‘dplyr’ v1.0.9 (Wickham et al., 2022) and ‘plyr’ v1.8.7 (Wickham, 2011) for data manipulation; ‘fitdistrplus’ v1.1.8 (Delignette‐Muller & Dutang, 2015) for fitting distributions to data; ‘ggpubr’ v0.4.0 (Kassambara, 2020) for enhancing the presentation of ggplot2 graphics; ‘ggrepel’ v0.9.1 (Slowikowski, 2021) for adding non‐overlapping text labels to plots; and ‘reticulate’ v1.26 (Ushey et al., 2022) for interfacing between R and Python, enriching our analysis capabilities.

The model included three entities: populations, age cohorts and individual *B. hookeriana* plants. The entities and their state variables are outlined in the ‘Initialization’ section of the standardized visual Overview, Design concepts, and Details (vODD; Szangolies et al., under review) as presented in Figure A1. Populations correspond to the geographical populations of *B. hookeriana* located in suitable habitat patches on dune crests, which are defined as areas with deep sands that support the complete life cycle of the species, contrasting with the uninhabitable intervening depressions. They were characterized by their unique id, carrying capacity (i.e. the maximum number of adult plants per unit area that can be sustained), time since last fire, habitat quality, list of cohorts entities, grid cell locations of the dune and grid cell locations of cone dispersal by birds (see below). A key aspect tracked for populations was their status, including whether they were extinct (defined as having no remaining individual plants, thus representing empty but suitable habitat patches), recolonized or burnt. Cohorts were characterized by the age of the plants, the number of plants, whether the cohort was established by LDD seeds, the average size of the canopy cone bank per plant—where the cones were classified according to their age (used only in the cohort‐based approach), and the list of individual plants (used only in the individual‐based approach). In addition to characterizing cohorts by attributes such as age, number of plants and dispersal type, we implemented a triple identifier system for tracking the development and spatial dynamics of these cohorts from seed dispersal to plant maturity across populations throughout the simulation. This system comprises three key identifiers: the ‘initial population ID’, which is the unique identifier of the population where the seeds that initiated the cohort were dispersed; the ‘previous population ID’, used to trace the source population of these seeds in the preceding generation and to track immigration rates; and the ‘current population ID’, which reflects the population where these seeds have established and grown into plants.

By differentiating between ‘previous’ and ‘current’ population IDs, we can dynamically categorize cohorts originating from seed dispersal as either ‘residents’ or ‘immigrants’ based on their dispersal and establishment events across populations. For example, a cohort that is initiated by seeds dispersing from population ID 1 and establishing in population ID 2, transitioning from (1, 1, 1) to (1, 1, 2), is denoted as an immigrant in the new location. This methodical tracking facilitates a comprehensive investigation into the dynamics of population structure across the landscape.

The plant entity was included as a model expansion to include intraspecific variation in plant performance within dunes (i.e. individual‐based approach). Details on the analysis and implementation related to this aspect are elaborated further in the discussion on cone production and storage that follows later in this section.

**FIGURE 1:**
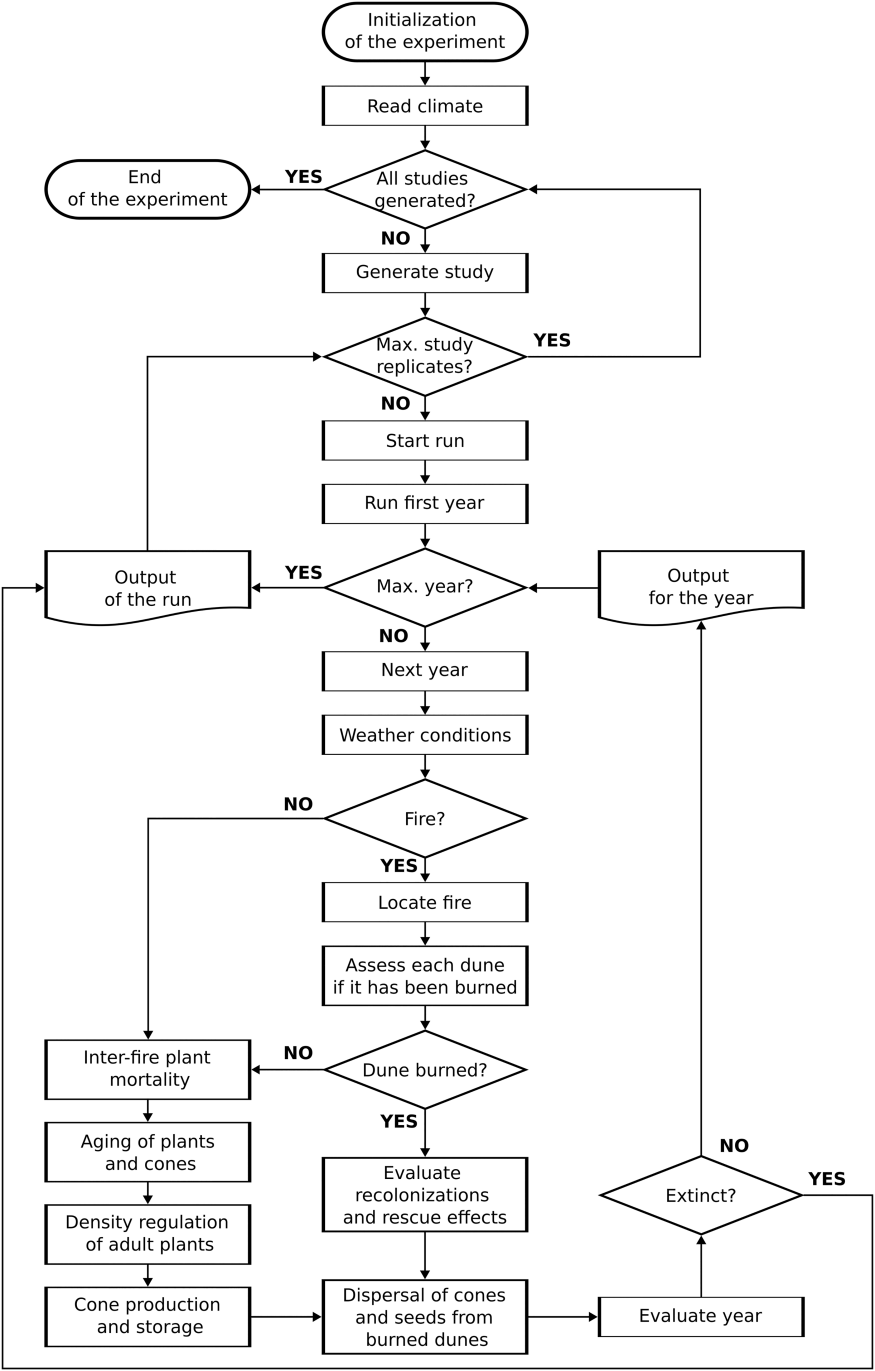
Key processes of the metapopulation model of *B. hookeriana*. Detailed description in the main text.

The most important processes of the model are presented in Figure 1. The initialization phase of the experiment involves validating input parameter values and calculating an essential derived parameter for computational efficiency. A key aspect of this pre‐computation phase is the determination of viable seeds per cone age, based on empirical observations of seed development, losses from insect damage, decay and sporadic rupture of follicles, as detailed in our previous work (Souto‐Veiga et al., 2022). As a reference, a 1‐year‐old cone produced under baseline conditions had approx. 10 viable seeds, and under current conditions, approx. seven viable seeds. This approach ensures that our simulations of seed dispersal and recruitment dynamics following burn events are both efficient and firmly rooted in empirical data. Then the climate data (specifically, rainfall data) were read in, which contained three predictor variables formed from the real rainfall conditions of the weather station closest to the study area (Eneabba Weather station nr 08225; Australian Bureau of Meteorology): winter–spring rainfall from previous year, annual rainfall from previous year, and sum winter–spring rainfall of the last 3 years. These variables were selected based on their established correlation with flower production and inter‐fire plant mortality as demonstrated in our previous study (Souto‐Veiga et al., 2022) and foundational work (Keith et al., 2014), making them essential for understanding the ecological responses of *B. hookeriana*. Next, the study area replicate was generated. The study area covered a 3 km × 5 km area of Eneabba Sandplain in South‐West Australia (He & Lamont, 2010), comprised of 100 m × 100 m grid cells. One mature plant was assumed to require an area of 2 m × 2 m (Esther et al., 2008), that is carrying capacity used in the process of density regulation of adult plants. The metapopulation contained 37 geographical populations (i.e. dunes), each defined by a unique population ID, area size (indicating variation in dune sizes with the range from 1 to 54 ha, rounded to the nearest whole number to match the grid cell resolution), initial individuals in the simulation, and habitat quality (Table A1). The metapopulation consisted of 18 initially occupied dunes and 19 unoccupied dunes.

Dunes were randomly located in the study area using a random walk algorithm, imitating the mosaic of irregular shape dunes of the Eneabba Sandplain landscape (He et al., 2010). This algorithm initiates with a randomly selected grid cell for each dune and sequentially adds adjacent cells through a process of equal‐probability selection, ensuring that each dune's specified area is accurately represented in terms of both size and shape. The minimum distance between dunes was maintained at 100 m to reflect the natural spacing observed in the Eneabba Sandplain (He et al., 2010).

All experiments commenced with the first simulation year subjecting all dunes to a burn event, simulating the scenario that reflects the last significant wildfire in 1998, which affected the entire study area (He et al., 2010). This approach not only aligns with the documented fire history of the Eneabba region (Miller et al., 2007) but also ensures a fair comparison across simulation scenarios. Following this initial event, all plants die and disperse their viable seeds, setting the stage for subsequent regeneration and population dynamics.

After the first year simulation, the yearly life cycle of the *B. hookeriana* metapopulation continued until either maximum number of simulated years was reached (500 years), or the metapopulation went extinct, meaning that all populations within the metapopulation had no remaining individual plants. Each year, which started at the end of the fire season (approx. April), began (1) by picking the weather conditions at random from the climate input data, followed (2) by evaluating whether fire occurred in the study area. Although our model can simulate both deterministic or stochastic fires (see Appendix B), we focused on deterministic fires only. If fire occurred, a patchy area was explicitly located at random in the study area and each dune was assessed as regards whether it was burnt or not (see Section 2.2.3).

In case of an occupied dune was burnt, all plants of the population died, and the total number of available fertile cones and viable seeds ready for dispersal for each cohort within the burned population were calculated. The calculation of seeds available in each cohort was made by multiplying the pre‐computed parameter ‘viable seeds per cone age’ during the initialization step of the simulation by the number of fertile cones within each age category and summing across all categories.

After calculating the total of fertile cones and viable seeds for each cohort across all the burned populations, seed dispersal for each cohort occurred as follows: Initially, 7% of the cones were dispersed through LDD of cones by birds (Section 2.2.1). Subsequently, 15% of the remaining viable seeds were dispersed by LDD of seeds by post‐fire wind (Section 2.2.2). These percentages were established during the calibration process using pattern‐oriented modelling (Railsback & Grimm, 2019), as detailed in Section 2.3. Seeds not dispersed by these LDD mechanisms were categorized as SDD seeds and stayed in the source population.

After dispersing all seeds, new cohorts were formed from seeds that landed on burned populations, as recruitment between fires was not considered; *B. hookeriana* recruits almost solely under post‐fire conditions, and the contribution of recruitment between fires to the population's seed dynamics was considered negligible (Souto‐Veiga et al., 2022). Seeds dispersed through the two LDD mechanisms initiated LDD cohorts. Seeds from a new LDD cohort that landed within the source population labelled the cohort's individuals as residents. Conversely, if seeds were dispersed to a different population, the individuals were labelled as immigrants. Seeds resulting in SDD always led to cohorts being classified as residents, emphasizing that only seeds dispersing to and germinating in burned areas contributed to new cohort formation.

In contrast, for populations that remained unburned in a given year—either due to the absence of fire events in the study area or because of patchy fires that did not reach these populations (see Section 2.2.3)—the following processes were carried out in sequence: inter‐fire plant mortality, aging of plants and cones, density regulation of adult plants and production and storage of cones.

With regard to inter‐fire plant mortality in our model, we drew from empirical datasets on annual mortality rates of *B. hookeriana* plants analysed by Keith et al. (2014). These datasets represent plants experimentally burned at two closed sites, one in spring and another in autumn, at Eneabba during 1988–2002, covering plant ages 1 to 15 years. A critical distinction between the spring and autumn datasets emerged in the mortality rates for the early years of plant life. Specifically, the autumn dataset revealed markedly higher mortality rates for plants aged 1 to 3 years. This increase in early mortality rates at the autumn site was attributed to higher seedling densities, suggesting density‐dependent mortality (Enright & Lamont, 1989). Utilizing this data, we were able to construct five scenarios of inter‐fire plant mortality, each exploring different ecological assumptions derived from the same foundational mortality rate data:

1. Age‐weather relative impacts: Previously used in our study (Souto‐Veiga et al., 2022), follows a hierarchical approach by Keith et al. (2014). It first calculates a base mortality rate from plant age (combining both datasets from autumn and spring sites), then adjusts it according to weather conditions. Notably, Keith et al. (2014) discovered a linear correlation between the deviation of age‐standardized mortality rates and the prior year's winter–spring rainfall, indicating a 6% mortality rate adjustment for every 100 mm change from the average rainfall. This method highlights how both age and lag rainfall intricately affect plant survival between fires, providing a detailed foundation for our model's mortality assumptions.
2. Mean age‐weather absolute impacts: In this scenario, we averaged the mortality rates from separate spring and autumn dataset regressions, providing a balanced mortality baseline.
3. LDD cohorts have lower mortality than SDD cohorts: Informed by He et al. (2009), this scenario postulated that LDD seeds, potentially landing away from competitive hotspots, might enjoy higher survival rates. Thus, in this scenario, LDD cohorts (both resident and immigrant LDD cohorts) used the fitted curve of the spring site and SDD cohorts of the autumn site.
4. Immigrant cohorts have lower mortality than resident cohorts: Also informed by He et al. (2009), this scenario explored the possibility of immigrant seeds having better survival due to inherited drought‐resistance traits. Thus, in this scenario, immigrant cohorts used the spring site fitted curve, and resident cohorts from the autumn site.
5. Lower intraspecific competition at earlier life stages under current climate conditions: The reduced vegetation cover in Mediterranean‐type ecosystems due to more frequent and intense droughts under current climate conditions (Carvalho et al., 2022; Daliakopoulos et al., 2017; Dong et al., 2019) may paradoxically lead to decreased competition for resources such as light, nutrients, and water. Thus, in this scenario, all plants in the metapopulation (i.e. no difference among cohorts) selected the lowest mortality from the two fitted curves of the spring and autumn sites.

Figure A2 shows the inter‐fire plant mortality curves for five different levels of total winter–spring rainfall for the different mortality scenarios above mentioned for comparison. For a more detailed account of these scenarios and the underpinning empirical data, readers are directed to Appendix B.

As for density regulation of adult plants, this process was applied once plants reach maturity at 5 years (Enright et al., 1996). Here, new mature plants can only be established if the population of mature plants is below the carrying capacity threshold. Otherwise, the plant dies. All new mature plants have the same probability of establishing as young adult plants.

Finally, the last process addressed for the unburned populations is the production and storage of cones. Adult plants (≥5 years old; Enright et al., 1998) produce new cones when weather conditions are favourable, thus increasing the canopy cone bank. Regarding cone production and storage, two approaches were utilized: the cohort‐based approach and the individual‐based approach.

The cohort‐based approach for calculating mean flower production in plants employs a two‐step hierarchical method, as described by Souto‐Veiga et al. (2022). This method begins with the calculation of potential flower production based solely on the age of the plant. Subsequently, the calculated value is modified by weather conditions. The analysis incorporates two periods of annual flower count data: a baseline dataset from 1988 to 2002 for establishing a historical perspective, and a more recent dataset from 2008 to 2017, applied to current climate scenarios. In the baseline dataset, a linear correlation was identified between mean annual flower production and the winter–spring rainfall of the previous year (Keith et al., 2014). The recent dataset revealed two significant predictors for flower production: the total annual rainfall of the previous year and the sum of the last 3 years' winter–spring rainfall. To analyse these relationships, linear mixed‐effect models were employed, offering a nuanced framework for capturing the variability across different plants and environmental conditions. For more details on the methodology, see Appendix B.

For the individual‐based approach, the number of flowers produced per plant each year was determined from fitted density curves, utilizing the current flower count data from 2008 to 2017. This approach allowed for the inclusion of intraspecific variation in plant performance within populations, specifically under the current climate scenario. It underpins our analysis of flower and cone production by classifying plants into ‘poor producers’ (below the 75th percentile in accumulation of cones) and ‘good producers’ (above the 75th percentile). This classification not only highlights survival rates—indicating that ‘good producers’ have significantly higher survival rates (Figure A3)—but also suggests local‐scale variation in habitat quality within dunes, likely due to differential access to soil water. Predictor variables, as identified in Souto‐Veiga et al. (2022)—namely, previous annual rainfall and the sum of winter–spring rainfall over the last 3 years—were applied in different membership functions following the fuzzification stage of fuzzy logic theory (Zadeh, 1988) (Figure A4). These membership functions, constructed using triangle and trapezoid shapes, effectively capture the impact of climate on flower production. For each plant performance class—‘poor’ and ‘good producers’—the best fitting model was selected from three common discrete density functions: Poisson, negative binomial, and geometric distribution, based on the lowest AIC value (Table A2; Figure A5).

After calculating the flowers produced, coefficients of mean increase in flower production were applied to reflect the observed dune habitat quality. These classifications—‘low’, ‘moderate’ and ‘high’—were based on flower count data from 2016 across plots with plants aged from 18 to 44 years post‐fire. As flower production in *B. hookeriana* stabilizes from 15 years post‐fire onwards, differences in plant age among plots do not affect comparability (Enright et al., 1996; Souto‐Veiga et al., 2022). A single plot in Eneabba represented the ‘low’ quality habitat, serving as our study area's reference location with a coefficient of 1.00, indicating no change in flower production. For ‘moderate’ quality, observed in three plots within the Eneabba Reserve area, we calculated the coefficient as 1.38 by comparing their average flower production to that of the Eneabba plot. The ‘high’ quality habitat, identified in a plot in South Eneabba with the highest flower production, was assigned a coefficient of 2.23, determined by dividing its flower count by the reference plot's average flower count. These coefficients, relative to the ‘low’ quality benchmark, allow our model to adjust flower production predictions based on habitat quality, with detailed methodology and comparative data illustrated in Figure A6 and Table A3. Finally, a percentage of flowers were converted into fertile cones (90% under baseline conditions, and 65% under current conditions; Souto‐Veiga et al., 2022) and the resultant number of fertile cones was stored in the state variable, canopy seed bank.

At the end of the year, the current state of the metapopulation was evaluated, summarizing various output metrics such as the number of recolonizations, the number of extinct populations and other more specific metrics which depend on the simulation type being executed (see Section 2.5).

#### 2.2.1 LDD of cones by birds

We considered dispersal of cones as direct dispersal; that is, birds dispersed a cone within the same population or among populations. The calculation of the number of seeds available for dispersal within each cone before birds disperse them was as follows: First, the number of follicles was drawn randomly from the fitted density curve for the baseline or current climate scenario (Figure A7). The best fit for the baseline scenario was the negative binomial distribution (size = 6.22, and mu = 10.08), and for the current scenario was the Poisson distribution (lambda = 7.33). Then, the number of viable seeds in each cone is calculated for 1‐year‐old cones, because cockatoos were most likely to remove recently produced cones that are located on the growing outer edges of the canopy. The remaining viable seeds for each dispersed cone were calculated by picking a random proportion of open follicles from 0.5 to 1 from a uniform distribution because 50% of the follicles were open 2 h after a fire (Enright & Lamont, 1989). The cone with the remaining viable seeds was finally dispersed by randomly picking one of the grid cells within the maximum distance dispersal observed (1250 m; He et al., 2004).

#### 2.2.2 LDD of seeds by post‐fire wind

Viable seeds can be dispersed within the source population (resident), in another population (immigrant), and in unsuitable areas or outside the study area (lost seed). Any viable seed to be dispersed by LDD through post‐fire wind went through the following steps: First, the starting point of dispersal was randomly selected within the population. Second, a random angle was selected from a uniform distribution since no pattern was observed in the data (He et al., 2010). Third, a random distance was chosen from the dispersal kernel, which was fitted to the observed immigrants (He et al., 2010). The best fit was a log‐normal distribution (meanlog = 6.79, sdlog = 0.68; Table A4). Finally, the drop point was calculated using the Euclidean distance formula considering the calculated origin point, angle and distance.

#### 2.2.3 Patchy fires

**FIGURE 2:**
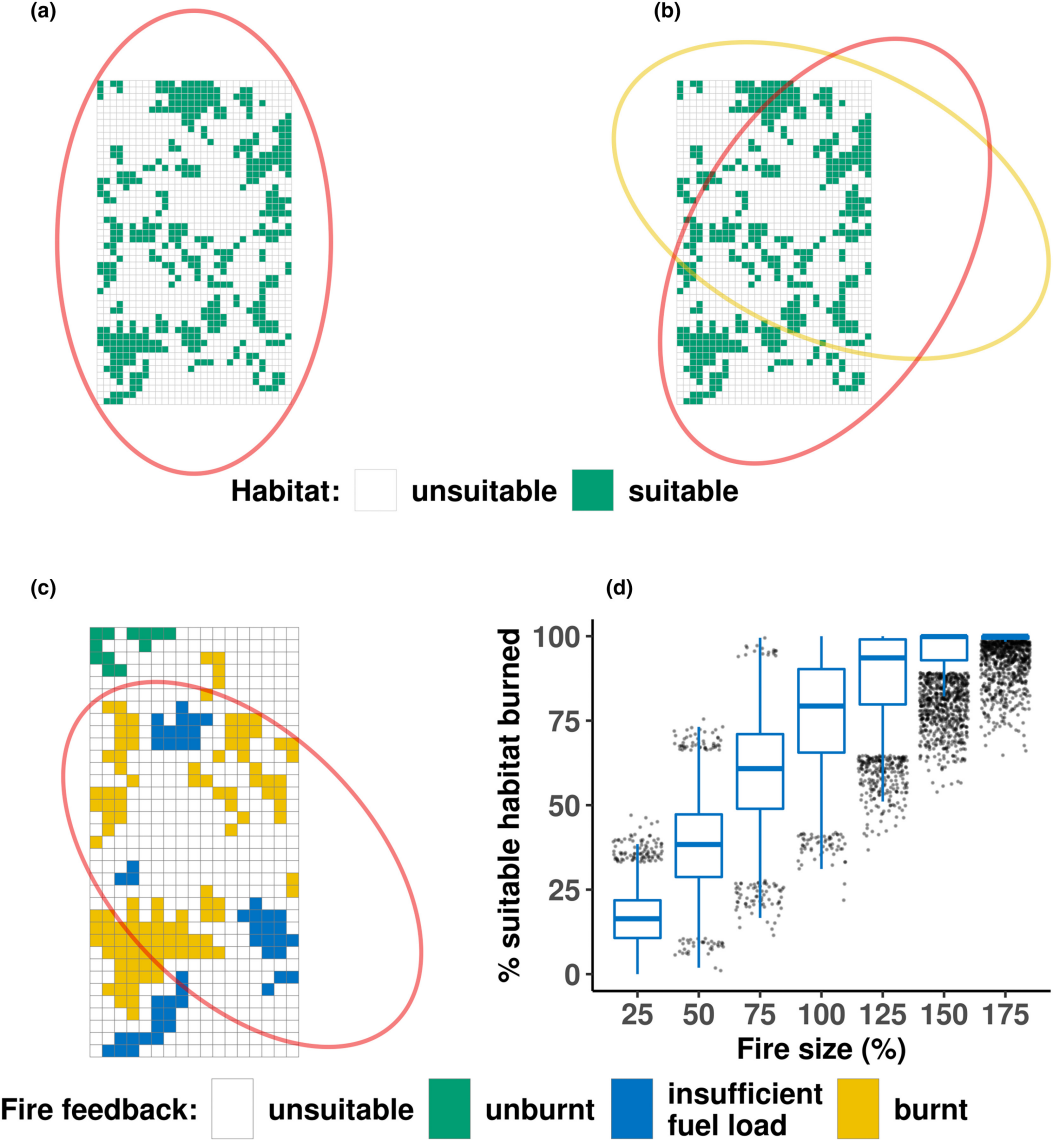
Demonstration of patchy fire events. (a) Circumscribed ellipse represents the baseline fire size (100%). (b) Two 100% fire size events with different centre point positions and orientations. (c) Possible population feedback to patchy fires: unburnt (green) because it is outside of the fire ellipse, unburnt due to insufficient fuel load (blue), and burnt (orange) population from being partly overlapped or fully covered by the fire ellipse and with enough fuel load. The boxplot in (d) shows the burnt suitable area distribution for all fire sizes tested in the study.

Regarding patchy fire events, we chose the ellipse shape as it is the most used fire shape in ideal conditions (Glasa & Halada, 2008; Green, 1983) and most fires in the region are wind‐driven with elliptical shapes (Enright et al., 2012). We defined as ‘baseline’ patchy fire size the circumscribed ellipse, that is, the ellipse passing through all vertices of the study area boundaries (Figure 2a). Patchy fire events were randomly located given the centre grid cell, the size (*x* and *y* semi‐axes), and the angle orientation (Figure 2b). A population was assessed as either completely burned or not burned at all, based on any degree of overlap with the patchy fire ellipse, no matter how minimal (Figure 2c). This initial assessment was further refined by considering the population's fuel load, which was determined using the fire spread formula of Groeneveld et al. (2008). A critical factor in this assessment was the time elapsed since the population's last fire, as it is a derivative of fuel load; a greater time since last fire signifies more vegetation, thereby increasing the fuel load. When the time since the last fire in a population or ‘patch’ exceeded 12 years, the accumulated fuel load was considered sufficient to carry a fire, hence the population was always burned (i.e. there was a 100% probability of the population being burned if an ignition occurred). A minimum interval of 3 years post‐fire was needed for a population to become eligible for burn assessment. Between 3 and 12 years, the probability of a population burning increased linearly based on our established assumptions and the fire spread formula. Figure 2d shows the distribution of burned suitable area for each fire size tested.

### 2.3 Model calibration

While most model parameters were directly sourced from existing data or literature (Appendix B for a comprehensive list), a few key parameters required detailed calibration to accurately simulate the ecological dynamics of *B. hookeriana* metapopulations within the Mediterranean‐type ecosystems of the Eneabba Sandplain.

The calibration of our model employed pattern‐oriented modelling, with a particular emphasis on inverse modelling techniques (Gallagher et al., 2021; Railsback & Grimm, 2019). This approach was essential not only for fine‐tuning specific LDD parameters—such as the percentage of cones dispersed by birds and the percentage of seeds dispersed by wind—but also for calibrating initial number of plants for each initially occupied population. These elements are critical within the broader LDD processes and population dynamics, directly impacting the model's ability to replicate observed patterns of seed dispersal, plant mortality and complex population structures.

Initial number of plants was specifically calibrated to match the 8‐year‐old population density reported by He et al. (2010). This effort was informed by the five inter‐fire plant mortality scenarios outlined in Section 2.2. Such calibration aimed to ensure consistency in representing population dynamics under a variety of ecological assumptions: (i) age‐weather relative impacts, (ii) mean age‐weather absolute impacts, (iii) LDD cohorts with lower mortality than SDD cohorts, (iv) immigrant cohorts with lower mortality than resident cohorts and (v) lower intraspecific competition at earlier life stages under current climate conditions.

In focusing on the LDD mechanisms, we meticulously calibrated two critical parameters: the ‘percentage of LDD cones by birds’ at 7% and the ‘percentage of LDD seeds by wind’ at 15%. Our calibration efforts were specifically aimed to replicate observed patterns within 8‐year‐old populations, as documented in the research by He et al. (2004, 2010). These patterns include immigration rates reported at 5.5% and 6.8%, respectively, and the crucial ecological structure where each initially occupied dune supports at least two population IDs, a condition also observed in He et al. (2010).

To this end, we embarked on a thorough examination of all combinations within the ranges from 0 to 20% for both parameters, incrementing in 1% steps. The benchmark of 7% for the dispersion of cones by birds was inspired by Witkowski et al. (1994), who observed that approximately 7% of flowers were removed by cockatoos. Although this reference primarily focused on pre‐seed‐set impacts, it provided a plausible estimate for post‐fire cone dispersal.

It was determined through the calibration process that a 15% dispersion rate of seeds by post‐fire wind was the minimum required to simultaneously match both critical observed patterns, as illustrated in Figure A8. Importantly, only under mortality scenarios (iii) and (iv)—which account for differential mortality rates among cohort types—did the model successfully replicate the observed immigration rates within a ±10% error margin (i.e. 4.95%–7.48%) and the presence of at least two population IDs per initially occupied dune.

### 2.4 Sensitivity analysis

Building on the methodology employed in Souto‐Veiga et al. (2022), we conducted a local sensitivity analysis to evaluate the robustness of our model outcomes against variations in parameter values. This analysis involved varying one parameter at a time by ±5% and ±10% to examine the influence of each on the mean metapopulation persistence (Table A5). The analysis was based on 900 replicates, comprising 30 study area replicates with each being simulated 30 times, under current climate conditions with deterministic fire intervals, with a set maximum persistence time of 500 years. We assessed the sensitivity of our model to parameter changes by calculating the percentage deviation between the mean persistence times of the reference values and those of each varied parameter.

This sensitivity analysis aimed to identify parameters that significantly affect the model's predictions, thereby highlighting critical factors that influence the persistence of metapopulations under current climate conditions. By rigorously examining how small changes in parameters influence model outcomes, we gained insights into the relative importance of different ecological processes and parameters, enhancing our understanding of metapopulation dynamics within the specific context of current environmental conditions.

### 2.5 Simulation experiments

Table 1 shows the five simulation experiments conducted in this modelling study. The simulation runtime was 500 years or until the metapopulation became extinct. All scenarios had 30 randomly generated replicates of study areas, and each study area replicate was replicated 30 times (i.e. a total of 900 simulation runs per scenario). The visualization of the experimental results shows the grand mean and pooled standard deviation of the output metric. In Experiment 1 and in two subplots of Experiment 5, only the grand mean is shown, since in the first case we were dealing with three‐dimensional plots and in the second with percentage units.

**TABLE 1:** Details of the five simulation experiments tested.

Experiment	Output metric	Driver			Plant performance	
		Climate	Pollination success	Fire^a^	Inter‐dune variation	Intraspecific variation
1. Spatio‐temporal fire impacts under different scenarios	Metapopulation persistence	Baseline, current	Baseline, current	5–30 years in 1 steps, and 25–175% in 25% steps	All ‘low’ habitat quality	No variation (mean)
2. Effects of lower competition	Metapopulation persistence	Current	Current	5–30 years in 1 steps, and 100%	All ‘low’ habitat quality	No variation (mean)
3. Effects of intraspecific variation of plant performance and dune habitat quality	Metapopulation persistence	Current	Current	21 years, and 100%	All dunes set to ‘low’, ‘moderate’, or ‘high’ habitat quality, without inter‐dune variation	‘Poor’ and ‘good’ producers, and survival increase of good producers from 0% to 27% in 3% steps
4. Effects of intraspecific variation of plant performance and inter‐dune variation	Metapopulation persistence	Current	Current	21 years, and 100%	Varied dune qualities (‘low’ to ‘high’), incrementally increasing ‘high’ quality dunes, with ‘moderate’ scenarios in appendix	‘Poor’ and ‘good’ producers, and 6% survival increase of good producers
5. Effects of climate change on metapopulation dynamics	Recolonization, immigration‐caused population growth, sink‐to‐source growth, and population persistence	Baseline, current	Baseline, current	21 years, and 100%	‘Low’ and ‘high’ habitat quality	‘Poor’ and ‘good’ producers, and 6% survival increase of good producers only under current climate conditions

^a^ Time in years and size in % of the circumscribed ellipse of the study area.

The metapopulation dynamics were assessed using five key output metrics (see Table 1 and Figure A1): (i) Persistence time (the principal metric in this study) refers to the duration for which the metapopulation or a population, respectively, maintains at least one living plant. For this study, long‐term persistence is defined as the ability of the metapopulation to sustain at least one living plant for a period exceeding 400 years, a threshold selected to evaluate resilience against environmental and demographic fluctuations over extended timeframes. (ii) Dune occupancy is the mean percentage of occupied dunes in the metapopulation throughout the simulation run, providing insight into spatial distribution and habitat utilization. (iii) Recolonization success was calculated as the percentage of total colonized and recolonized populations that were extinct or unoccupied at the start of the simulation experiment, which measures the ability of populations to recover. (iv) Immigration‐caused population growth success indicates the percentage of living populations that increased their number of individuals through immigration from other populations, highlighting the contribution of immigrant individuals to population growth. (v) Sink‐to‐source growth quantifies the number of immigrant plants from sink populations in source populations that survived until the next fire, illustrating the impact of immigration on metapopulation renewal. These metrics recolonization and immigration‐caused population growth were measured at four plant stages: seeds (viable seeds that fall within a suitable patch), seedlings (1‐year‐old plants), adults (5‐year‐old plants) and source plants (adult plants with canopy seed banks that reach the next fire), thereby capturing the lifecycle dynamics essential for understanding metapopulation persistence.

To ensure a comprehensive understanding of the hierarchical structure and rationale behind the simulation experiments, it is essential to refer to Figure A1 (the vODD). Within this figure, a specific table in the ‘Scenarios’ section details the principal levels of complexity and aspects of model development and feature implementation used in each experiment. This table within the figure visually supplements the narrative that follows, highlighting how each experiment builds upon the insights gained from its predecessors. The structure of these experiments is designed to systematically dissect the complex interplay between fire regimes, intraspecific competition and variation in plant performance under changing climatic conditions.

Experiment 1 establishes a foundational understanding of how different spatio‐temporal fire regimes affect metapopulation dynamics under both baseline and current climatic conditions, serving as a benchmark for assessing changes in persistence over time. Subsequently, Experiments 2 through 4 delve deeper into the current climate scenario, each focusing on specific ecological processes and assumptions. Experiment 2 examines the potential mitigating effects of reduced intraspecific competition due to lower plant densities on metapopulation persistence. Experiment 3 then explores the impact of observed intraspecific variation in plant performance, while Experiment 4 combines insights from the previous experiments to assess the joint influence of intraspecific and inter‐patch variation in plant performance. Finally, Experiment 5 synthesizes these findings by comparing optimized scenario—incorporating the key factors identified as beneficial for persistence under current conditions—to baseline climate conditions.

This hierarchical and iterative approach enables a nuanced exploration of the factors influencing *B. hookeriana* metapopulation dynamics, revealing the pivotal roles of fire regimes, competition and plant performance variation in shaping responses to climatic change. By structuring the experiments in this manner, we aim to isolate and understand the relative contributions of these factors to metapopulation persistence, guiding conservation efforts in fire‐prone landscapes.

## 3 RESULTS

### 3.1 Spatio‐temporal fire impacts under different scenarios

The key finding of Experiment 1 was that metapopulation dynamics did not lead to longer‐term regional survival of *B. hookeriana* under current conditions and basic model assumptions.

**FIGURE 3:**
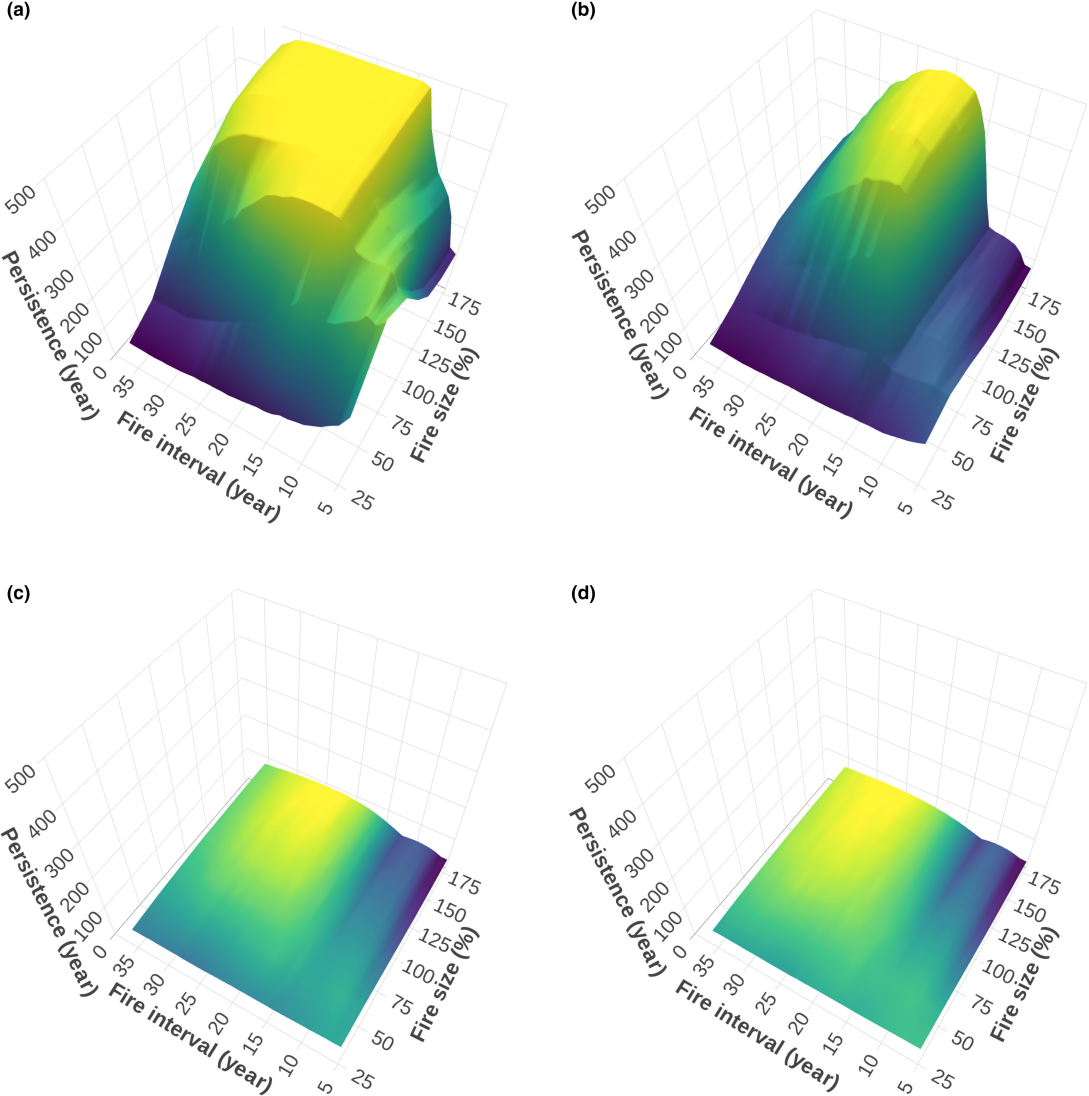
Experiment 1: Mean metapopulation persistence of the simulated *B. hookeriana* metapopulation (*z*‐axis) depending on fire interval (5 to 30 years in increments of one) and fire size (25% to 175% of the study area in increments of 25%) under (a) baseline climate conditions, (b) change in pollination decline only (i.e. percentage of fertile cones from 90% to 65%, and the number of follicles per cone from 9.97 to 7.32), (c) climate change only (i.e. rainfall from 1988–2002 to 2003–2017), and (d) combined climate and pollination change.

Baseline conditions, a fire size of 100%, and fire intervals of 14–24 years led to the highest persistence times (>495 years; Figure 3a), with the intervals increasing in range with fire size: for example, increasing the fire size to 175% expanded the range of fire intervals to 14–29 years, further extending the conditions for achieving high persistence. Interestingly, very short fire intervals reduced metapopulation persistence considerably and require patchy fires to be smaller for optimal persistence. For instance, under baseline conditions (Figure 3a), the highest persistence times with a fire interval of 5 years occurred with fire sizes of 50% (persistence of 434 years) (see Figure A9 for this specific fire interval scenario). The effects of declining pollination success and fruit set alone (Figure 3b) slightly reduced the maximum persistence time from 500 to 482 years. In this scenario, the optimal fire interval window shrunk, for example, to 16–23 years with 100% fire size (with a persistence time of 404–442 years). The effects of climate change alone on metapopulation dynamics were so severe that they led to a decrease in survival from 500 to 110 years, as shown in Figure 3a,c. Under current climate conditions, the effects of changing fire intervals and fire sizes on survival were insignificant. This context underscores that, when comparing the sole effect of climate change to the additional impact of reduced pollination success, climate change emerges as the primary driver of decline in metapopulation persistence. Reduced pollination success further decreases persistence by 18%, underscoring the dominant role of climate change (Figure 3c,d). To reduce complexity in presenting all the following results, fire size was held at 100% when testing all other mechanisms.

### 3.2 Effects of lower competition under current conditions

**FIGURE 4:**
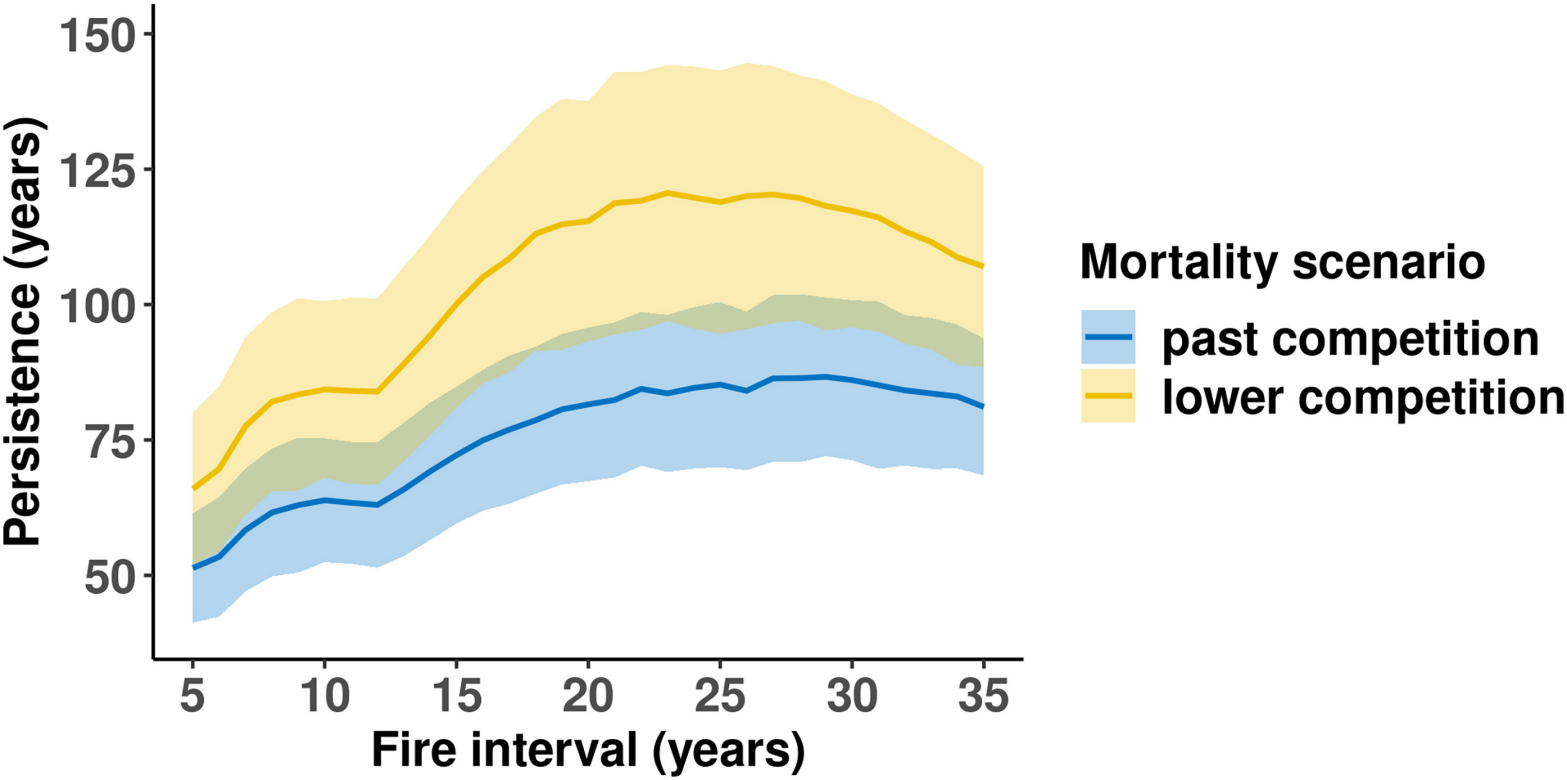
Experiment 2: The metapopulation persistence time (*y*‐axis) of two mortality scenarios under current climate‐pollination conditions: past (blue) and current (yellow) competition. The current mortality scenario was assumed to have lower mortality rates at earlier life stages due to lower population densities. Each mortality scenario was tested with fire intervals from 5 to 30 years (*x*‐axis) with a fire size of 100%. Lines show the grand mean values and the shaded areas are the ± pooled standard deviation.

Assuming there was lower plant competition at earlier life stages under current climatic conditions as a result of reduced plant density, the survival time of the metapopulation increased by only 15–37 years compared to the (higher) past mortality scenario (Figure 4). In general, the persistence time increased with increasing fire interval and reached the highest level at fire intervals of 21–29 years with approx. 120 years persistence. We chose a fire interval of 21 years for the following experiments because this was the mean fire interval observed at Eneabba (Enright et al., 2012) and was within the simulated optimal fire interval window (Figure 4).

### 3.3 Effects of intraspecific variation of plant performance and dune habitat quality on metapopulation persistence

**FIGURE 5:**
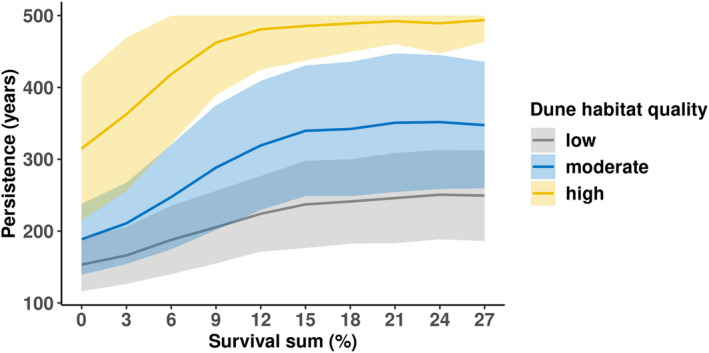
Experiment 3: Effects of intraspecific variation of plant performance (e.g. caused by trait variation or by microsite variation of the dune habitat) on metapopulation persistence (*y*‐axis) under current conditions. In this experiment, the survival of ‘good producers’ (i.e. plants that had a high seed production, see Figure A1) increased from 0% to 27% in 3% incremental steps (*x*‐axis). Three dune habitat quality scenarios (i.e. coefficient of the mean increase in flower production) are compared: (i) low (grey), (ii) moderate (blue) and (iii) high (yellow). All 37 dunes were assigned to the same quality in each habitat quality scenario. The fire interval used was 21 years, and the fire size was 100%. Lines show the grand mean values and the shaded areas are the ± pooled standard deviation.

As expected, for all three dune habitat quality scenarios the persistence of the metapopulation increased with an increasing survival of plants that had a higher seed production (‘good producers’, see Figure A3; Figure 5). However, the metapopulation only achieved long‐term survival (i) when all 37 dunes were classified as high habitat quality, and (ii) when the survival rate of ‘good producers’ was at least 6% higher than that of ‘poor producers’ (i.e. reached 500 years persistence with one standard deviation). For example, with a 6% survival rate and high habitat quality, metapopulation persistence was 418 ± 97 years (grand mean ± pooled standard deviation). Assuming that plants with higher seed production did not have an increased survival rate only led to minor increase of metapopulation persistence of 12–37 years compared to complete invariability in plant performance (Figure A10).

### 3.4 Effects of intraspecific variation of plant performance and inter‐dune variation on metapopulation persistence

**FIGURE 6:**
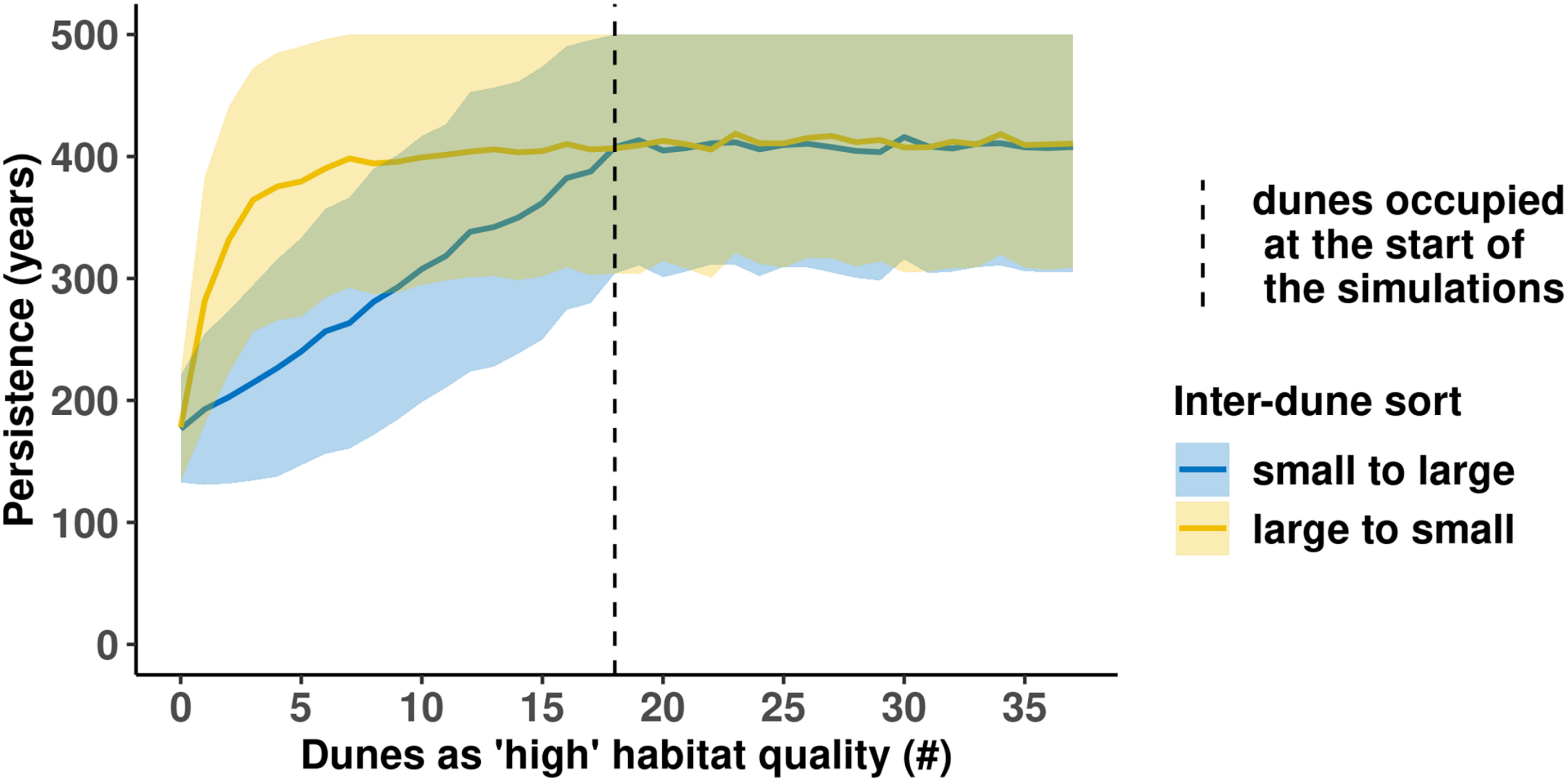
Experiment 4: Effects of intraspecific variation of plant performance and inter‐dune variation on metapopulation persistence (*y*‐axis) under current conditions. ‘Good producers’ were assumed to have a 6% higher survival. The fire interval was 21 years, and the fire size was 100%. In this experiment, the number of ‘high’ habitat quality dunes was systematically increased from 0 to 37 dunes (*x*‐axis) with all other dunes being of ‘low’ habitat quality. The inclusion order of ‘high’ habitat quality dunes depended on dune habitat size and was either classified from small to large (blue) or from large to small (yellow). The sorting of the dunes was done in two steps: first, the initially occupied dunes, and then the initially unoccupied dunes. All initially occupied dunes start with the same plant density (seven plants per ha). Lines show the grand mean values and the shaded areas are the ± pooled standard deviation.

Combining intraspecific variation of plant performance and inter‐dune variation increased persistence time of the metapopulation by 104 years (i.e. from 178 ± 45 to 282 ± 102 years) when only the largest dune was classified as high quality (Figure 6). Classifying the seven largest occupied dunes as high habitat quality was sufficient to achieve a maximum long‐term persistence of approx. 400 years (500 years persistence with one standard deviation). Conversely, when sorting from small to large dune habitats (blue), all initially occupied dunes had to be included to achieve maximum persistence. In addition, persistence did not increase further with other fire interval scenarios (Figure A11). In contrast to adding a small number of high‐quality dune patches, the inclusion of moderate quality dunes had no significant effect on metapopulation persistence and average dune occupancy (Figure A12). Also, dune occupancy only increased when dunes with high quality were included.

### 3.5 Effects of climate change on metapopulation dynamics

**FIGURE 7:**
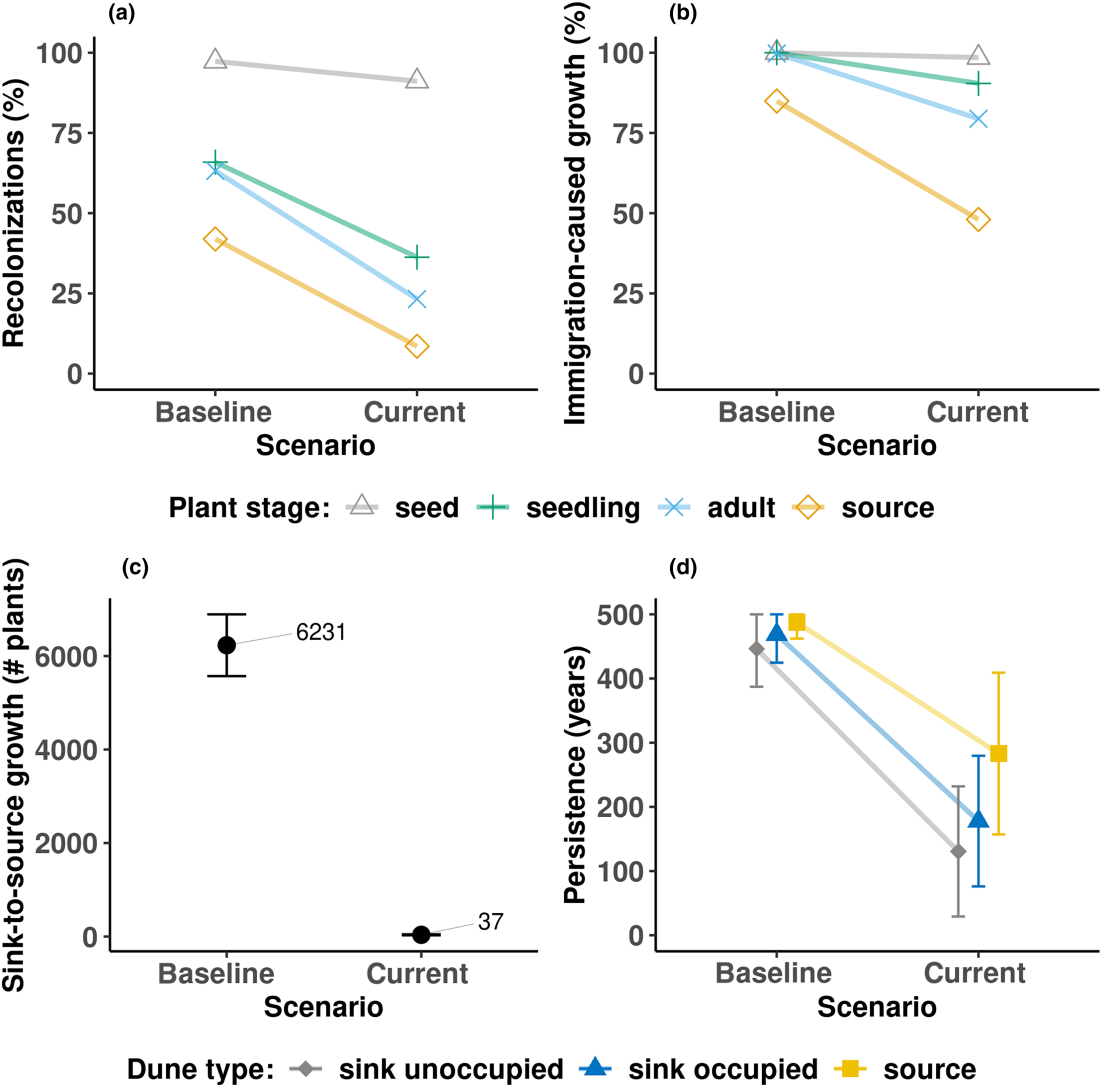
Experiment 5: Effects of climate change on metapopulation dynamics (i.e. baseline vs. current climate scenarios) using four output metrics: (a) recolonizations (i.e. mean percentage of the total colonized and recolonized populations), (b) immigration‐caused population growth (i.e. mean percentage of living populations that increased their number of individuals through immigration), (c) sink‐to‐source growth (i.e. number of immigrant plants in the source populations that made it to the next fire) and (d) persistence of source and sink dunes. Fire interval was 21 years, and the fire size was 100%. The seven largest occupied dunes were assigned as high habitat quality (i.e. sources), and the rest as low (sinks).

Even under the most optimistic scenarios tested before the *B. hookeriana* metapopulation at Eneabba changed from a reasonably stable metapopulation to a non‐equilibrium source‐sink dynamics (Figure 7). Regarding recolonization success (Figure 7a), under baseline conditions, almost all extinct dunes were successfully recolonized at the seed stage (97%). More than 60% of recolonizations reached the seedling and adult stages (66% and 63% respectively). The success of recolonization by source plant stage (adults plant with canopy seed bank that reach next fire) was 42%. In contrast, under current conditions, while recolonization remained relatively high at the seed stage (91%) it dropped at all other plant stages with only 9% by source plant stage (Figure 7a). Under baseline conditions, the immigration‐caused population growth (Figure 7b) were practically 100% for the seed, seedling and adult stages and 85% for the source stage. Under current conditions, the immigration‐caused population growth was reduced to 98%, 90%, 79% and 48% for the germination, seedling, adult, and source stages respectively (Figure 7b). The number of plants from sink to source strongly decreased from 6231 to 37 plants (Figure 7c) when baseline and current climates are compared. Correspondingly, persistence time (Figure 7d) decreased under current conditions from 446 ± 59 to 131 ± 101 (for the initially unoccupied sink‐dunes), from 469 ± 44 to 177 ± 102 (for the initially occupied sink‐dunes) and from 487 ± 25 to 283 ± 126 (for the source‐dunes).

### 3.6 Sensitivity analysis

Most of the model parameters were robust to ±5% and ±10% shifts from their reference values, that is, this variation of the parameters showed little effect on metapopulation persistence (Table A6). For example, all alterations of LDD parameters did not affect metapopulation persistence by more than 1%. The most sensitive parameters were those related to post‐fire recruitment and flower production, where the persistence time changed by two and three times from the alteration of these parameters. Post‐fire recruitment was more sensitive to flower production parameters. A 10% decrease in the upper limit and mean of post‐fire recruitment mortality parameters increased metapopulation persistence by approx. 60%.

## 4 DISCUSSION

In our previous study, we showed that isolated populations of *B. hookeriana* will not survive long‐term under current climate conditions (Souto‐Veiga et al., 2022). Here, we extended the model to a metapopulation framework that includes the explicit regional process of LDD, and wildfires as the primary disturbance. The model was parameterized using data from the Eneabba region for the period 1988 to 2018, and we tested several optimistic hypotheses to determine if long‐term persistence is possible under current (climate data 2003–2017 cf. baseline data 1988–2002) conditions at Eneabba. In general, metapopulation dynamics could increase the overall regional survival of *B. hookeriana*. However, it became clear that this potential for increased survival, even under optimistic scenarios, was limited by a transition from traditional metapopulation dynamics—where each patch can independently support a viable population—to source‐sink dynamics in a strict sense. This shift exposed the reality that some patches (sinks) required support from others (sources) to sustain their populations, underscoring the profound impact of current climate conditions on habitat quality and the viability of populations.

### 4.1 Plant metapopulation and fire regime

Our modelling shows the importance of large fires that enable a correlated *seed release—long distance dispersal—colonization* dynamics between different (sub‐)populations of *B. hookeriana*. Simulations showed that more than 61%–79% of the suitable area had to be burned to achieve maximum persistence of the spatially structured population. This finding clearly has to be contrasted with multi‐species studies that advocate using pyrodiversity to support population hotspots of multiple species (Durigan & Ratter, 2016; He et al., 2019). Interestingly, our finding is also in clear contrast to general metapopulation theory which suggests that such systems can only persist when critical correlation length of the local extinction processes is short, that is, patch‐level extinction is largely uncorrelated (Durigan & Ratter, 2016; but see Bullock et al., 2020). For fire‐prone metapopulations such as *B. hookeriana* these general assumptions do not hold since both processes, seed release and recolonization of empty patches, require a correlated occurrence of fire. Overall, our simulations suggest that the *B. hookeriana* metapopulation dynamics require large fires with intermediate to long inter‐fire intervals (specifically, 21–29 years) to ensure long‐term survival.

In the context of fire regimes, species persistence and adaptation are profoundly influenced by key factors such as frequency, size, season and severity of fires (Keeley et al., 2011). This becomes particularly relevant when considering our finding that larger fire sizes can significantly contribute to species resilience through enhanced LDD, a crucial insight given the historical and contemporary understanding of fire regimes and their management practices. Throughout Australia, consideration of the potential ecological and social benefits of cultural burning (indigenous‐led burning seeking to emulate pre‐European practices where possible) is spreading rapidly (Fletcher et al., 2021; Mariani et al., 2022). Typically the practices included burning at shorter interval, smaller size and lower severity relative to post‐European settlement managed‐ and wild‐fire regimes. Long practiced in the tropical north where strong indigenous knowledge persists (but not in temperate Australia) there is considerable debate about when and where cultural burning might be applied. This debate rests on our understanding of past burning which often is limited. Enright and Thomas (2008) examined the early European historical record for the Mediterranean‐type shrublands region which supports *B. hookeriana*. They conclude that Aboriginal peoples likely concentrated their activities, including cultural burning, near the coast and in river valleys where water and food were most available. Results suggest that populations of *B. hookeriana* in these dry shrublands may have been more conditioned by infrequent, larger wildfires (from lightning or people) rather than frequent, small patchy burns most closely associated with more mesic woodlands and forests in higher rainfall areas. Such observations also accord with the recent record where wildfire has tended to burn large areas covering many *B. hookeriana* populations (Enright et al., 2012).

Contemporary fire management often is tasked with balancing wildfire risk and biodiversity considerations with substantial uncertainty as global heating progresses. The interval squeeze hypothesis clearly posits, and our modelling confirms, that longer fire intervals (relative to recent mean fire recurrence times) are required for population persistence. This is counter to the perspectives of many government agency fire practitioners where focus on fire hazard and its elevated levels under climate change warrant an ‘adaptive management’ response of more frequent burning and therefore shorter fire intervals in order to reduce the available fuel and thus the risk of high severity wildfires. Fire management is becoming more complex: as well as needing to consider the potential role of indigenous cultural burning as part of the formal fire‐fighting tool‐kit, and responding to climate change‐induced shifts in fire frequency and severity, climate change is also reducing the number of available suitable days per year for managed burns (Clarke et al., 2022) and increasing the risk of fire escapes which may burn unintended critical habitat (Black et al., 2020). In the case of *B. hookeriana*, the evidence presented here suggests that small fire size may not provide the source‐sink dynamics required for long‐term persistence, while increasing risk of managed fire escapes could impact unintended populations (e.g. young stands), also compromising long‐term persistence.

### 4.2 Within patch and among patch processes

One of our tested assumptions was to differentiate plant performance within populations (i.e. patches). We classified plants into poor and good producers according to empirical data. Data indicated that good producers also had higher survival rates with this binary classification of plant performance. Interestingly, we found no trade‐off between maintenance and reproduction. This is in contrast to other studies claiming that plant strategies are either optimized to expend energy on maintenance or on reproduction and that those that expend energy on reproduction are more susceptible to droughts and other adverse effects (Huxman et al., 2008; Wang et al., 2015). A possible explanation for our contrasting result could be that the sources for an observed better performance of certain individuals were better local microsite conditions (e.g. nutrients, moisture) rather than intraspecific genetic trait variations. However, since that was not investigated here we do not exclude any alternative explanation.

We found that achieving long‐term regional persistence, defined as exceeding 400 years, necessitates not only intraspecific variation in plant performance within patches—specifically, granting a 6% higher survival rate to good producers—but also requires that several key patches exhibit higher habitat quality, as evidenced by classifying the seven largest patches as high‐quality habitats.

Regarding variation within populations, other studies also have identified the existence of superior individuals. For example, Smith et al. (2022) classified two different oaks with masting strategies as a function of their plant performance. They observed that super producers, although a small percentage within a population, contribute a significant amount of seed at the population level. We made some isolated observations (not used in this study) of *B. hookeriana* individuals in a different geographic area that could be classified as super producers (up to 200 flowers). This goes beyond the increased seed production of ‘good producer’ individuals considered for this study. Such additional super producers could further contribute to the population‐level seed bank for *B. hookeriana* metapopulation dynamics. However, given the current lack of data this remains speculative.

Our findings on the significance of ‘super producers’ within *B. hookeriana* populations align with broader ecological studies emphasizing the role of individual heterogeneity in plant population dynamics. For instance, a study on *Plantago lanceolata* highlighted that non‐selective demographic variation significantly influences survival and reproduction, suggesting a substantial impact of individual stochasticity on population dynamics (Steiner et al., 2021). These insights underline the critical importance of incorporating individual variability into models to accurately predict ecological and evolutionary outcomes. Such approaches could be particularly relevant in understanding the dynamics of Mediterranean ecosystems under changing climate and fire regimes.

On the regional scale, our findings suggest that it is important to have some source habitat patches in the landscapes that are of better quality than others. Such differences in patch quality can have a buffering effect in case of deteriorating environmental conditions. In our case, these differences enabled a shift from a traditional metapopulation to a source sink dynamics that at least prevented a rapid extinction. Also, the connectivity among patches may change in time with changes in the abundance of animal vectors (de Paula Mateus et al., 2018), pollinators (Ramos‐Jiliberto et al., 2020), and changes in the wind patterns (Keith et al., 2020). Dispersal is the main mechanism to maintain stable metapopulations by recolonizations, and gene flow. Surprisingly, during the calibration of the percentage of LDD seeds—which can be dispersed within the same source population or to other populations—we had to assume that these seeds had lower mortality compared to SDD seeds (i.e. those solely dispersed within the source population) to obtain realistic values for the proportion of seeds dispersed by post‐fire wind. Although we tested a wide range of proportions for LDD seeds and cones (from 0% to 20%) in our calibration process, it remains possible that our modelling of LDD mechanisms—specifically, seeds dispersed by post‐fire wind and cones by birds—may reflect an underestimation of the actual number of LDD seeds both produced and effectively dispersed. He et al. (2009) attributed the high immigration rate in our studied metapopulation (approx. 7%) to two possible causes: (i) LDD seeds have a higher probability of falling in places where there is less competition than SDD seeds, and (ii) immigrant seeds from sites with lower habitat quality may be better adapted to drought and other causes of decline. It is important to note that post‐fire wind dispersal is expected to increase due to climate change (warm or dry season fires) by increasing the frequency and intensity of wind updrafts. Wind vertical updrafts are the most sensitive factor to reach LDD of seeds (He et al., 2004). Warm or dry season fires encourage more frequent and stronger post‐fire updrafts than fires that occur in other seasons (Dunker et al., 2019). Since climate change is increasing the fire season time span, Keith et al. (2020) predict that these fires will result in lower extinction risks because more seeds will be dispersed, there will be more room for recolonization, and there will be more gene flow. Future work should investigate the effects of the new wind patterns on the ability of fire‐killed, serotinous species to compensate local extinctions by recolonizing suitable habitat patches.

### 4.3 Model sensitivities, shortcomings and future directions

Process‐based modelling can identify which processes or group of processes significantly affect the dynamics of the system and which parameters are susceptible to changes. We discovered that most model parameter changes (±5% and ±10%) had little effect on the metapopulation's ability to persist. A few parameters, such as those related to post‐fire recruitment, one of the three main drivers of the Interval Squeeze theory put forth by Enright et al. (2015), had an excessively significant impact on the metapopulation's persistence. Our previous non‐spatial population study obtained similar results (Souto‐Veiga et al., 2022). In addition, ongoing monitoring plans on flower and cone production are also needed to improve model parameterization and predictions. In particular, new monitoring plans should be established for other metapopulations located in better environmental and habitat conditions to determine whether these metapopulations are also at risk of becoming unstable metapopulations.

We here left out the possibility of having successful recruitment between fires because we found earlier that introducing an inter‐fire recruitment probability did not change the persistence probability substantially (Souto‐Veiga et al., 2022). Although climate change is generally reducing recruitment success due to more frequent and intense droughts, the vegetation density and cover are also reducing in fire‐prone regions (Carvalho et al., 2022; Daliakopoulos et al., 2017; Dong et al., 2019). This may not only reduce competition‐induced mortality but also positively affect inter‐fire recruitment, that is, less competition for light. If this is true, inter‐fire recruitment within and among patches may become more important for metapopulation persistence under future conditions. Thus we encourage putting effort into post‐fire recruitment studies and investigate the possible positive effects of inter‐fire recruitment under current and prospective climates.

Our model results also highlight the potential relevance of intra‐population variation in plant traits. Here, further experiments are needed to quantify intraspecific trait variation in plants within and among patches focusing on cone production, survival, recruitment, adaptation and phenotypic plasticity.

Furthermore, the impact of shifting climate and fire regimes on Mediterranean ecosystems extends beyond our study species, *B. hookeriana*, to influence the broader plant communities they are part of. Notably, inter‐specific interactions between key non‐sprouter species, like *B. hookeriana*, and key resprouter species may undergo significant changes as these environmental conditions evolve. Models that explore these dynamics could offer valuable insights, especially considering that the ratio of non‐sprouter to resprouter species across Australian ecosystems tends to increase with decreasing rainfall (Lawes et al., 2022). However, the dynamics driving this pattern remain poorly understood. Future research could beneficially investigate how these inter‐specific interactions might shift and their demographic consequences on *B. hookeriana* and similar species within Mediterranean ecosystems, thus providing a more comprehensive understanding of the ecological impacts of climate change and altered fire regimes.

## 5 CONCLUSION

We found that the consequences of continuing climate change on plant demographics are so severe that the existing metapopulation dynamics shift to an unstable source‐sink dynamic state, even under optimistic assumptions. One of the main bottlenecks to persistence is the decline in recruitment success, which should be compensated by other mechanisms. From the conservation perspective, *B. hookeriana* (and likely other similar fire‐killed species) must have relatively long and large fires to reach optimal survival. This contrasts with the contemporary procedure of fire managers, that is, using short, small and low‐intensity fires. Our study also highlights the importance of variation, both within and among populations. Deciphering the role of trait variation becomes even more important under conditions of population decline when a superior performance of a few individuals can make the difference between population extinction and survival. As shown here, also on the regional level of metapopulation dynamics the variation between subpopulations and habitat patches can play a crucial role for a potential buffering transition towards a source‐sink dynamics under conditions of climate change.

## AUTHOR CONTRIBUTIONS

**Rodrigo Souto‐Veiga:** Conceptualization (lead); data curation (lead); formal analysis (lead); investigation (lead); methodology (lead); software (lead); visualization (lead); writing – original draft (lead); writing – review and editing (equal). **Juergen Groeneveld:** Conceptualization (supporting); investigation (equal); methodology (supporting); supervision (equal); writing – original draft (supporting); writing – review and editing (equal). **Neal J. Enright:** Conceptualization (supporting); funding acquisition (equal); investigation (equal); methodology (supporting); supervision (equal); writing – original draft (supporting); writing – review and editing (equal). **Joseph B. Fontaine:** Conceptualization (supporting); funding acquisition (equal); investigation (equal); methodology (supporting); supervision (supporting); writing – original draft (supporting); writing – review and editing (equal). **Florian Jeltsch:** Conceptualization (supporting); funding acquisition (equal); investigation (equal); methodology (supporting); supervision (equal); writing – original draft (supporting); writing – review and editing (equal).

## CONFLICT OF INTEREST STATEMENT

The authors declare no conflict of interest.

### OPEN RESEARCH BADGES

This article has earned Open Data, Open Materials and Preregistered Research Design badges. Data, materials and the preregistered design and analysis plan are available at [[insert provided URL(s) on the Open Research Disclosure Form]].

## Supporting information


Data S1

## Data Availability

The complete set of files, including the source code for the model, data analysis and visualization scripts, along with supporting data, has been deposited in the Dryad digital repository and is available under the following doi:10.5061/dryad.zpc866tgr. Additionally, the model code and scripts (without the supporting data) continue to be available on GitHub for development and collaboration: https://github.com/rsoutoveiga/metasqueeze‐model.
