# Supplementary material for: Climate change may shift metapopulations towards unstable source‐sink dynamics in a fire‐killed, serotinous shrub
Source: Ecol Evol. 2024 Jun 3;14(6):e11488. doi: 10.1002/ece3.11488 (PMC11148395; doi:10.1002/ece3.11488)
Supplement: Supplementary file 1 — Data S1 [file ECE3-14-e11488-s001.zip › ece311488-sup-0001-SupinfoS1/sm_0001-Supinfo.docx]

# Appendix A

## Tables

**Table A1** Initialization input file of the metapopulation. Eighteen habitat patches (i.e., population ID) were initially occupied, and the other 19 were unoccupied

| **Population ID** | **Size (ha)** | **Individuals** | **Habitat quality** |
| --- | --- | --- | --- |
| 1 | 21 | 5 | low |
| 2 | 14 | 254 | low |
| 3 | 47 | 826 | low |
| 4 | 49 | 917 | low |
| 5 | 4 | 34 | low |
| 6 | 54 | 292 | low |
| 7 | 8 | 151 | low |
| 8 | 10 | 2 | low |
| 9 | 22 | 62 | low |
| 10 | 5 | 66 | low |
| 11 | 7 | 88 | low |
| 12 | 5 | 72 | low |
| 13 | 3 | 14 | low |
| 14 | 14 | 171 | low |
| 15 | 16 | 93 | low |
| 16 | 16 | 196 | low |
| 17 | 8 | 65 | low |
| 18 | 18 | 255 | low |
| 19 | 3 | 0 | low |
| 20 | 8 | 0 | low |
| 21 | 2 | 0 | low |
| 22 | 2 | 0 | low |
| 23 | 3 | 0 | low |
| 24 | 15 | 0 | low |
| 25 | 3 | 0 | low |
| 26 | 10 | 0 | low |
| 27 | 2 | 0 | low |
| 28 | 4 | 0 | low |
| 29 | 4 | 0 | low |
| 30 | 9 | 0 | low |
| 31 | 4 | 0 | low |
| 32 | 4 | 0 | low |
| 33 | 5 | 0 | low |
| 34 | 5 | 0 | low |
| 35 | 3 | 0 | low |
| 36 | 1 | 0 | low |
| 37 | 13 | 0 | low |

**Table A2** Flower count probability density functions for poor and good producers. The Fuzzy set "low" corresponds to Fig. A4b, and "high" to Fig. A4c. It is noted that Fig. A4a represents the initial transitional fuzzy set used to select between these two detailed fuzzy sets

| Fuzzy set | Membership function | Flower producer class | Distribution type | Distribution parameters |
| --- | --- | --- | --- | --- |
| low | low | poor | none | zero flowers |
|  |  | good | none | zero flowers |
| low | medium | poor | Poisson | lambda = 0.071 |
|  |  | good | Poisson | lambda = 0.111 |
| low | high | poor | geometric | prob = 0.673 |
|  |  | good | geometric | prob = 0.288 |
| high | very low | poor | geometric | prob = 0.618 |
|  |  | good | geometric | prob = 0.415 |
| high | low | poor | Poisson | lambda = 1.214 |
|  |  | good | geometric | prob = 0.250 |
| high | medium | poor | geometric | prob = 0.203 |
|  |  | good | negative binomial | size = 3.254,  mu = 8.443 |
| high | high | poor | geometric | prob = 0.205 |
|  |  | good | negative binomial | size = 6.275,  mu = 10.100 |

**Table A3** Coefficients of mean increase in flower production based on habitat quality, derived from 2016 flower count data. Eneabba (HD1, 18 years post-fire) serves as the 'low' quality reference (coefficient of 1.00). 'Moderate' quality from Eneabba Reserve plots (26–44 years post-fire) has a coefficient of 1.38, and 'high' quality at South Eneabba (23 years post-fire) has a coefficient of 2.23. Post-fire ages are provided, underscoring that flower production stabilizes after 15 years

| Site (plot) | Habitat quality | Mean of flowers (µF)* | Coefficient of mean increase in flower production |
| --- | --- | --- | --- |
| Eneabba (HD1) | Low | 6.367 | 1.00 (Reference) |
| Eneabba Reserve (SER.E3, SER.E4, and SER1990) | Moderate | 8.761 | 1.38 (µF of Moderate / µF of Low) |
| South Eneabba (MPS1993) | High | 14.200 | 2.23 (µF of High / µF of Low) |

*Refer to Fig. A6 for the distribution of flower counts.

**Table A4** The AIC values of the three probability density functions fitted to the observed immigration data in He et al. (2010)

| **Distribution type** | **AIC value** |
| --- | --- |
| lognorm | 363.44 |
| gamma | 363.80 |
| Weibull | 364.79 |

**Table A5** Parameter values used in the local sensitivity analysis, conducted under current climate conditions with deterministic fire intervals. This table presents the reference values for each parameter alongside their variations at –10%, –5%, +5%, and +10%. Parameters that were truncated due to their nature (e.g., probabilities) are indicated with their percentage of variation in parentheses

| **Parameter** | **Reference** | **Variation** | | | |
| --- | --- | --- | --- | --- | --- |
|  |  | **–10%** | **–5%** | **+5%** | **+10%** |
| carrying_capacity | 2 500 | 2 250 | 2 375 | 2 625 | 2 750 |
| init_cones | 85.87 | 77.28 | 81.58 | 90.16 | 94.46 |
| init_seeds | 623.14 | 560.83 | 591.98 | 654.3 | 685.46 |
| long_term_rain | 453.85 | 408.46 | 431.16 | 476.54 | 499.23 |
| fire_size_x | 2 121.320 | 1 909.188 | 2 015.254 | 2 227.386 | 2 333.452 |
| fire_size_y | 3 535.534 | 3 181.981 | 3 358.757 | 3 712.311 | 3 889.087 |
| fire_interval_mean | 17 | 15 | 16 | 18 | 19 |
| burned_lower_cut | 3 | 2 (–33.3%) | 2 (–33.3%) | 4 (33.3%) | 4 (33.3%) |
| burned_upper_cut | 12 | 10 (–16.7%) | 11 (–8.3%) | 13 (8.3%) | 14 (16.7%) |
| wind_prop | 0.15 | 0.14 | 0.14 | 0.16 | 0.17 |
| wind_a | 6.79 | 6.11 | 6.45 | 7.13 | 7.47 |
| wind_b | 0.68 | 0.61 | 0.64 | 0.71 | 0.74 |
| birds_dist_max | 1 250.000 | 1 125.000 | 1 187.500 | 1 312.500 | 1 375.000 |
| birds_prop | 0.07 | 0.06 | 0.07 | 0.07 | 0.08 |
| follicles_distr_a | 7.33 | 6.59 | 6.96 | 7.69 | 8.06 |
| follicles_distr_b | 0 | 0 | 0 | 0 | 0 |
| postfire_follicles_open | 0.5 | 0.45 | 0.48 | 0.53 | 0.55 |
| young | 5 | 4 (–20%) | 4 (–20%) | 6 (20%) | 6 (20%) |
| adult | 15 | 13 (–13.3%) | 14 (–6.7%) | 16 (6.7%) | 17 (13.3%) |
| recruit_post_min | 0.91 | 0.82 | 0.87 | 0.96 | 0.99 |
| recruit_post_max | 0.99 | 0.911 (–7.7%) | 0.94 | 1 (1.3%) | 1 (1.3%) |
| recruit_post_mean | 0.92 | 0.83 | 0.88 | 0.97 | 1 |
| recruit_weather | 0.06 | 0.05 | 0.06 | 0.06 | 0.07 |
| senescence_age | 25 | 22 (–12%) | 23 (–8%) | 27 (8%) | 28 (12%) |
| senescence_increase | 0.01 | 0.01 | 0.01 | 0.01 | 0.01 |
| mort_min | 0.02 | 0.02 | 0.02 | 0.02 | 0.02 |
| mort_a | 0.29 | 0.26 | 0.27 | 0.3 | 0.32 |
| mort_b | 0.0000 | 0.0000 | 0.0000 | 0.0000 | 0.0000 |
| mort_c | 0.32 | 0.29 | 0.3 | 0.33 | 0.35 |
| mort_d | 0.65 | 0.58 | 0.62 | 0.68 | 0.71 |
| mort_e | −0.001 | −0.00053 | −0.00056 | −0.00061 | −0.00065 |
| mort_f | 0.29 | 0.26 | 0.27 | 0.3 | 0.32 |
| cone_cycle | 1 | 0 (–100%) | 0 (–100%) | 2 (100%) | 2 (100%) |
| seed_longevity | 12 | 10 (–16.7%) | 11 (–8.3%) | 13 (8.3%) | 14 (16.7%) |
| flower_age_a | 9.179 | 8.261 | 8.720 | 9.638 | 10.097 |
| flower_age_b | 8.710 | 7.839 | 8.275 | 9.146 | 9.581 |
| flower_age_c | 0.621 | 0.559 | 0.590 | 0.652 | 0.683 |
| flower_weather_a | 0.050 | 0.048 | 0.051 | 0.056 | 0.058 |
| flower_weather_b | −15.212 | −13.691 | −14.452 | −15.973 | −16.734 |
| flower_weather_c | 0.022 | 0.02 | 0.021 | 0.023 | 0.024 |
| flower_weather_d | 0.023 | 0.021 | 0.022 | 0.024 | 0.025 |
| flower_weather_e | −33.345 | −30.010 | −31.677 | −35.012 | −36.679 |
| pollination | 65 | 58.5 | 61.75 | 68.25 | 71.5 |
| follicles | 7.32 | 6.59 | 6.95 | 7.69 | 8.05 |
| seeds | 2 | 1.8 | 1.9 | 2.1 | 2.2 |
| firm_seeds | 0.83 | 0.747 | 0.788 | 0.872 | 0.913 |
| viable_seeds | 0.744 | 0.67 | 0.707 | 0.781 | 0.818 |
| insect_a | 0.02 | 0.018 | 0.019 | 0.021 | 0.022 |
| insect_b | 0.18 | 0.162 | 0.171 | 0.189 | 0.198 |
| decay_a | 0.34 | 0.306 | 0.323 | 0.357 | 0.374 |
| decay_b | −5.950 | −5.355 | −5.652 | −6.247 | −6.545 |
| open_a | 0.39 | 0.351 | 0.37 | 0.409 | 0.429 |
| open_b | 6.687 | 6.019 | 6.353 | 7.022 | 7.356 |
| open_c | 0.959 | 0.863 | 0.911 | 1.007 | 1.055 |

**Table A6** Results of the local sensitivity analysis performed under current climate conditions with deterministic fire intervals. It shows the percent deviation of the simulation results for the mean persistence time, relative to variations in each parameter (-10%, -5%, +5%, +10%). When a parameter is changed, all other parameters remain constant, allowing for a focused assessment of each parameter's influence. For the reference values and their specific variations, see Table A5

| **Parameter** | **Variation** | | | |
| --- | --- | --- | --- | --- |
|  | **–10%** | **–5%** | **+5%** | **+10%** |
| carrying_capacity | –0.72 | –0.12 | –0.77 | –1.4 |
| init_cones | –0.29 | –1.15 | –0.34 | –1.46 |
| init_seeds | –1.89 | –0.24 | 0.05 | –0.53 |
| long_term_rain | 37.86 | 14.42 | –12.35 | –20.54 |
| fire_size_x | 0.24 | –0.72 | –0.57 | –1.26 |
| fire_size_y | –1.24 | –0.7 | –0.15 | –0.35 |
| fire_interval_mean | –7.92 | –3.42 | 1.6 | 3.81 |
| burned_lower_cut | –0.3 | –0.47 | –1.02 | –0.96 |
| burned_upper_cut | –0.84 | –1.13 | –0.59 | 0.24 |
| wind_prop | –1.09 | –1.16 | –1.15 | –1.19 |
| wind_a | 0.19 | –0.52 | –0.56 | –1.04 |
| wind_b | –1.1 | –0.97 | –0.46 | –0.35 |
| birds_dist_max | –0.9 | –0.07 | –0.62 | –0.72 |
| birds_prop | –0.41 | –0.54 | –0.54 | 0.14 |
| follicles_distr_a | –1 | –1.79 | –0.88 | –1.44 |
| follicles_distr_b | –1.55 | –0.29 | –1.59 | –0.9 |
| postfire_follicles_open | –0.15 | –0.71 | –0.33 | –1.12 |
| young | –0.8 | –0.99 | –0.67 | –0.86 |
| adult | –2.37 | –1.19 | –0.27 | –0.15 |
| recruit_post_min | –1.25 | 0.12 | –4.34 | –24.29 |
| recruit_post_max | 62.1 | 34.59 | –47.06 | –5.74 |
| recruit_post_mean | 57.67 | 38.6 | –23.38 | –24.94 |
| recruit_weather | 1.85 | –0.03 | –1.66 | –3.68 |
| senescence_age | –3.22 | –2.04 | –0.4 | 0.4 |
| senescence_increase | –0.82 | –0.27 | –1.15 | –1.58 |
| mort_min | –1.75 | –0.93 | –0.56 | –1.46 |
| mort_a | 2.69 | 1.91 | –2.28 | –3.51 |
| mort_b | –7.71 | –4.35 | 3.63 | 8.58 |
| mort_c | 13.52 | 6.21 | –6.14 | –11.68 |
| mort_d | 5.97 | 2.44 | –3.58 | –5.82 |
| mort_e | –8.88 | –5.11 | 4.15 | 9.3 |
| mort_f | 12.96 | 4.71 | –6.02 | –11.27 |
| cone_cycle | 2.9 | 1.66 | –2.83 | –2.12 |
| seed_longevity | –2.26 | –0.42 | –0.99 | –0.85 |
| flower_age_a | –3.59 | –1.87 | 1.04 | 2.18 |
| flower_age_b | 1.91 | 0.31 | –0.99 | –4.54 |
| flower_age_c | –1.86 | –0.35 | –1.44 | –0.34 |
| flower_weather_a | 9.55 | 3.79 | –3.28 | –6.79 |
| flower_weather_b | –5.23 | –2.36 | 1.53 | 3.54 |
| flower_weather_c | –10.56 | –5.89 | 3.16 | 9.11 |
| flower_weather_d | –22.88 | –14 | 12.68 | 26.61 |
| flower_weather_e | 32.69 | 15.72 | –16.18 | –27.68 |
| pollination | –3.64 | –2.03 | 0.47 | 1.8 |
| follicles | –3.09 | –2.5 | –0.15 | 2.63 |
| seeds | –3.77 | –3.02 | 1.1 | 2.29 |
| firm_seeds | –2.75 | –1.16 | 1.2 | 1.82 |
| viable_seeds | –2.18 | –2.81 | 0.61 | 1.07 |
| insect_a | 0.31 | –1.15 | –1.07 | –1.29 |
| insect_b | –0.39 | –0.3 | –1.19 | –0.52 |
| decay_a | –0.08 | –0.65 | –1.73 | –1.43 |
| decay_b | –0.36 | –0.91 | –1.24 | –0.79 |
| open_a | 0.35 | –1.5 | –0.92 | –0.71 |
| open_b | –0.49 | –0.31 | –0.32 | 0.05 |
| open_c | –0.53 | –0.53 | –1.52 | –0.54 |

## List of figure captions in Appendix A

**Fig. A1** Standardized vODD of our *B. hookeriana* metapopulation model. The figure outlines crucial model components, such as 'Initialization', 'Submodels', and 'Observations'. Additionally, the 'Scenarios' section is included to emphasize the hierarchical structure of our simulation experiments, showcasing the comprehensive approach taken in model development.

**Fig. A2** Annual mortality probability curve between fires by the age of the *B. hookeriana* shrub in Eneabba in different rainfall scenarios. Three mortality scenarios are shown: (a) age-weather relative impacts, (b) mean age-weather absolute impacts, and (c) always lowest mortality of age-weather absolute impact (lower intraspecific competition at earlier life stages under current climate conditions).

**Fig. A3** Classification of our flower and cone count data under current climate conditions: (a) plants were classified into two poor and good producers using the 75th percentile. (b) Flower count distribution per plant per year with the binary plant performance classification. (c) Good producers held a significantly (Mann-Whitney test) higher number of cones, and (d) had higher survival rates than poor producers.

**Fig. A4** Hierarchical fuzzy sets used to classify plant performance. (a) Initial transitional fuzzy set using 'sum of winter–spring rainfall of the last three years', where the membership function labeled as 'low' directs to fuzzy_set_low (b), and the membership function labeled 'high' directs to fuzzy_set_high (c). These subsequent fuzzy sets (b and c) detail the classification of flower counts into 'poor' and 'good' producers, with each flower count within each membership function fitted to a density curve (see Table A2 and Fig. A5 for related density functions).

**Fig. A5** Flower count probability density functions for poor (blue) and good (yellow) producers. Each subplot corresponds to a membership function in Fig. A4: (a) "medium", and (b) "high" membership functions in fuzzy_set_low (Fig. A4b). (c) "very low", (d) "low", (e) "medium", and (f) "high" membership functions in fuzzy_set_high (Fig. A4c). The coefficient values for each density distribution are in Table A2.

**Fig. A6** Flower count data from 2016 across Eneabba plots of varying post-fire ages, informing habitat quality categorization: 'low' (HD1, 18 years post-fire), 'moderate' (Eneabba Reserve: SER.E3, 29 years; SER.E4, 44 years; and SER1990, 26 years), and 'high' (South Eneabba: MPS1993, 23 years). Plant age is noted, although it does not significantly influence flower production beyond 15 years post-fire. The mean values served as a basis for calculating the habitat quality coefficients of mean increase in flower production, as outlined in Table A3.

**Fig. A7** (a) Number of follicles per fertile cone (i.e., cones with one or more follicles) in 1986 (baseline) and 2018 (current). Cones were burned to expose and rupture the follicles, and the number of follicles per cone was counted. In 2018 we collected cones aged 1, 3, and 5 years from 12 plants (n = 77 fertile cones) in a plot (HD8) near our current long-term monitoring plot (HD1). We compared these data with 1986 data for 1, 3, and 5-year-old cones from the same ten plants studied in the proportion of fertile cones (n = 243 fertile cones). A Mann-Whitney test showed that the number of follicles per cone was significantly higher for the baseline data (median = 10) than for the current data (median = 8), W = 12545.5, p < 0.0001. (b) The fitted density curves of the number of follicles per cone for the baseline dataset (blue, negative binomial distribution with size = 6.22 and mu = 10.08) and for the current dataset (yellow, Poisson distribution with lambda = 7.33).

**Fig. A8** Calibration results for LDD parameters via inverse modeling, showcasing the outcomes across the first four inter-fire plant mortality scenarios, which correspond to (a) age-weather relative impacts, (b) mean age-weather absolute impacts, (c) LDD cohorts with lower mortality than SDD cohorts, and (d) immigrant cohorts with lower mortality than resident cohorts, respectively. Each subplot examines a range of combinations between percentages of LDD of cones by birds (0–20%, y-axis) and LDD of seeds by wind (0–20%, x-axis), assessing their effectiveness in matching two empirical patterns: the percentage of immigrants and the number of population IDs per habitat patch. The model was considered successful in replicating these patterns when the immigrant percentage aligned with the observed immigration rate within a ±10% error (i.e., 4.95–7.48%) and when the number of population IDs was equal to or greater than two, as observed by He et al. (2004, 2010). The fifth mortality scenario is not shown, as its results mirrored those of scenarios (a) and (b), failing to differentiate mortality rates among cohort types and thus not providing additional insights into the calibration process aimed at replicating observed patterns of seed dispersal and population structuring within the *B. hookeriana* metapopulations on the Eneabba Sandplain.

**Fig. A9** Experiment 1 under baseline conditions with a fire interval of five years. Line shows the grand mean values and the shaded area is the ± pooled standard deviation.

**Fig. A10** Experiment 3 under current climatic conditions using the current mortality scenario. This experiment compares the intra-dune variation on flower production (yellow) against no intra-dune variation between plants (blue). Lines show the grand mean values and the shaded areas are the ± pooled standard deviation.

**Fig. A11** Effects of inter- and intra-dune plant performance on the metapopulation persistence (z-axis) under current conditions. Good producers had a 6% higher survival. The fire interval scenarios ranged from 5 to 35 years in 1-year incremental steps (x-axis), and the fire size was 100%. In this experiment, all dunes were initially assigned as ‘low’ habitat quality, and the number of ‘high’ habitat quality dunes was increased from 0 to 37 dunes (y-axis). The inclusion of ‘high’ habitat quality dunes was classified from small to large (blue) or from large to small (yellow). The classification of dune sizes was done in two steps: first, we sort the 18 occupied dunes at the beginning of the simulation, and then the unoccupied dunes.

**Fig. A12** Experiment 4 shows the effects of intraspecific variation of plant performance and inter- and intra-dune variation on the (a) metapopulation persistence, and (b) dune occupancy including ‘moderate’ habitat quality under current conditions. Good producers had a 6% higher survival. The fire interval was 21 years, and the fire size was 100%. The default state of dunes was ‘low’ habitat quality (x-value 0). We stepwise increased the number of habitat patches from low to high or moderate (x-axis) depending on the maximum number of high quality patches (indicated by colour). The sorting of the dunes was done in two steps: first, the initially occupied dunes, and then the initially unoccupied dunes. All initially occupied dunes start with the same plant density (seven plants per ha). It becomes evident that persistence and dune occupancy rapidly increases with high but not with moderate dune qualities. Lines show the grand mean values and the shaded areas are the ± pooled standard deviation.
