# Supplementary material for: Climate change may shift metapopulations towards unstable source‐sink dynamics in a fire‐killed, serotinous shrub
Source: Ecol Evol. 2024 Jun 3;14(6):e11488. doi: 10.1002/ece3.11488 (PMC11148395; doi:10.1002/ece3.11488)
Supplement: Supplementary file 1 — Data S1 [file ECE3-14-e11488-s001.zip › ece311488-sup-0001-SupinfoS1/3_Appendix_B_TRACE_document.pdf]

Supporting Information. TRACE documentation of model design, testing, and development.

TRACE document

This TRACE document ("TRAnsparent and Comprehensive model Evaludation") provides supporting evidence for the model presented in our study, detailing how climate change may influence metapopulation dynamics towards unstable source-sink behaviors in fire-killed, serotinous shrubs. It comprehensively covers the assumptions, methodologies, and findings, underscoring the robustness and implications of our research:

> **Souto-Veiga, R., Groeneveld, J., Enright, N. J., Fontaine, J. B., & Jeltsch, F. (2024). Climate change may shift metapopulations towards unstable source-sink dynamics in a fire-killed, serotinous shrub. *Ecology and Evolution*. Advance online publication. https://doi.org/10.1002/ece3.11488**

Corresponding author:

Rodrigo Souto-Veiga[1,2,3]

rodrigo.souto.veiga@uni-hamburg.de

ORCID: 0000-0001-8639-620X

[1] Plant Ecology and Nature Conservation, University of Potsdam, Potsdam, Germany

[2] School of Environmental and Conservation Sciences, Murdoch University, Murdoch, Western Australia, Australia

[3] Institute of Plant Science and Microbiology, Universität Hamburg, Hamburg, Germany

Table of Contents

1. Problem formulation........7

2. Model description........10

2.1 Purpose and patterns........11

2.2 Entities, state variables, and scales........11

2.3 Process overview and scheduling........14

2.4 Design concepts........18

2.5 Initialization........20

2.6 Input data........21

2.7 Submodels........23

3. Data evaluation........45

3.1 The parameters related to study area........46

3.2 The parameters and data related to metapopulation........46

3.3 The parameters related to fire........47

3.4 The parameters and data related to dispersal........48

3.5 The parameters and data related to plant demographics and characteristics........52

4. Conceptual model evaluation........64

4.1 Plant mortality........64

4.2 Seed production and storage........65

4.3 Fire........65

4.4 Dispersal........66

5. Implementation verification........67

6. Model output verification........68

7. Model analysis........71

8. Model output corroboration..75
Literature cited...76

Index of Tables

Table 1: Population state variables....................13
Table 2: Cohort state variables....................14
Table 3: Plant state variable....................14
Table 4: Vertex values defining each membership function in Fuzzy_set_1 (see Fig. 6a)....................40
Table 5: Vertex values defining each membership function in Fuzzy_set_low (see Fig. 6b)....................40
Table 6: Vertex values defining each membership function in Fuzzy_set_high (see Fig. 6c)....................40
Table 7: Flower count probability density functions for poor and good producers. The Fuzzy set "low" corresponds to Fig. 6b, and "high" to Fig. 6c....................40
Table 8: Parameters related to study area....................47
Table 9: Initialization input file of the metapopulation. Eighteen habitat patches (i.e., population ID) were initially occupied, and the other 17 were unoccupied....................47
Table 10: The parameters related to fire....................48
Table 11: The parameter related to dispersal....................49
Table 12: The AIC values of the three probability density functions fitted the observed immigration data (see Fig. 8)....................50
Table 13: The parameters related to plant demography....................53
Table 14: Habitat quality coefficients of mean increase in flower production....................59
Table 15: Classification of plant performance used in the main results in the main paper....................62
Table 16: Results of significant of the four linear-mixed models of flower production under current climate conditions (see Fig. 15)....................64
Table 17: Calibration of initial plants per population in each mortality scenario....................70
Table 18: Parameter values of reference and sensitivity analysis. The parameters that were truncated are shown in red next to their percentage of variation....................73
Table 19: Results of the local sensitivity analysis. The values indicated are the percent deviation of the simulation results of the mean persistence time. Each parameter was changed by –10%, –5%, +5% and +10%. When a parameter is changed, all other parameters remain constant. The parameters that were truncated are shown in red font (see Table xx). Results showing more than twice the percentage deviation in parameter change are highlighted....................74

Table of Figures

Fig. 1: Key processes of the metapopulation model of B. hookeriana. Detailed description in the main text........16

Fig. 2: The climate scenarios used in the simulation experiments: were baseline (1988–2002) and current (2003–2017). Three predictor variables were used in the model: a) winter–spring rainfall of the previous year, b) annual rainfall of the previous year, and c) sum of winter–spring rainfall of the last three years. Each predictor decreased on average by approx. 15%. The rainfall data were retrieved from the closest weather station to the study area, i.e., Eneabba Weather Station no. 8225. 22

Fig. 3: Demonstration of patchy fire events. (a) the circumscribed ellipse represents the baseline fire size (100%). (b) two 100% fire-size events with different center point positions and orientations. (c) possible population feedback to patchy fires: unburnt (green) because it is outside of the fire ellipse, unburnt due to insufficient fuel load (blue), and burnt (orange) population from being partly overlapped or fully covered by the fire ellipse and with enough fuel load. The boxplot in (d) shows the burnt suitable area distribution for all fire sizes tested in the study........24

Fig. 4: Annual mortality probability curve between fires by the age of the B. hookeriana shrub in Eneabba in different rainfall scenarios. Three mortality scenarios are shown: (a) age-weather relative impacts, (b) mean age-weather absolute impacts, and (c) always lowest mortality of age-weather absolute impact (lower intraspecific competition at earlier life stages under current climate conditions)........30

Fig. 5: The two flower production scenarios used in the simulation experiments. Each row corresponds to a scenario: on the left side the selected model fit of the relationship between annual flower production per adult B. hookeriana plant and rainfall prediction variables is shown, and on the right side the impacts of the left best-fit model to the age-standardized flower production model, which corresponds to the black line in Fig. 4b. The first row shows the best linear mixed-effect model between the flower count baseline data set (1988–2002, plant age 17–31 yeas) and the total rainfall in winter–spring of the previous year (i.e., lag winter–spring). The second row shows the best linear mixed-model between current flower count data set (2008–2017, plant age 11–20 years) and the two covariates: total annual rainfall from previous years (i.e., lag annual rainfall, in the x-axis) and sum of winter–spring rainfall of the previous three years (displayed as Viridis colour ramp). The weather impacts of each best-fit model on plant age (right side) is shown with the same amount of rainfall to compare the flower production scenarios [the second predictor in the current

data set (i.e., sum of winter–spring rainfall of the previous three years) was set to the average rainfall in the current climate scenario (1130 mm in 2003–2017)]. The shaded areas distinguish the status of the plant between seedling (grey), where all flowers failed to set seed, and adult (green), where a proportion of flowers are potentially pollinated..35

Fig. 6: The three fuzzy set functions used to apply the intraspecific variability of plant performance within populations (i.e., individual-based approach). (a) fuzzy_set_1, (b) fuzzy_set_low, and (c) fuzzy_set_high..38

Fig. 7: Flower count probability density functions for poor (blue) and good (yellow) producers. Each subplot corresponds to a membership function in Fig. 6: (a) "medium", and (b) "high" membership functions in fuzzy_set_2 (Fig. 6b). (c) "very low", (d) "low", (e) "medium", and (f) "high" membership functions in fuzzy_set_3 (Fig. 6c). The coefficient values for each density distribution are in Table 7..40

Fig. 8: The results of the three probability density functions fitted the observed immigration data (He et al., 2010)...49

Fig. 9: The fitted density curves of the number of follicles per cone for the baseline dataset (blue, negative binomial distribution with size = 6.22 and mu = 10.08) and for the current dataset (yellow, Poisson distribution with lambda = 7.33)...50

Fig. 10: Number of follicles per fertile cone (i.e. cones with one or more follicles) in 1986 and 2018. Cones were burned to expose and rupture the follicles, and the number of follicles per cone was counted. In 2018 we collected cones aged 1, 3, and 5 years from 12 plants (n = 77 fertile cones) in a plot (HD8) near our current long-term monitoring plot (HD1). We compared these data with 1986 data for 1, 3, and 5-year-old cones from the same ten plants studied in the proportion of fertile cones (n = 243 fertile cones). A Mann-Whitney test showed that the number of follicles per cone was greater for the 1986 data (median = 10) than for the 2018 data (median = 8), W = 12545.5, p < 0.0001. As model parameters, we used the mean number of follicles per cone, i.e., 9.97 follicles in 1986 (i.e. baseline scenario) and 7.32 in 2018 (current scenario)...51

Fig. 11: Linear regression analysis of the two mortality rate sites, i.e., spring site, and autumn site. The spring formula was as Mortality rate = mort_a / age + mort_b * lag rainfall + mort_c, where mort_a = 0.2877622, mort_b = –0.0005888, and mort_c = 0.3179949 with all the three coefficients at 0.05 level of significance, and the adjusted R-squared = 0.435. The autumn formula was as Mortality rate = mort_d / age + mort_e * lag rainfall + mort_f, where mort_d = 0.6475991, mort_e = –0.0006502, and mort_f = 0.2867799 with all the three coefficients at 0.01 level of significance, and the adjusted R-squared = 0.844..56

Fig. 12: Relationship between cone age and the proportion of follicles open per cone in 1986 in Eneabba. The black curve represents the best fitted logistic growth model and the shaded area the 95% CI. The model is: OpenFollicles(cone_age) = a / (1 + exp(c (b – cone_age)), where a = 0.3896,

b = 6.6873 years, and c = 0.9588 years–1, with 95% CIs [0.2903, 0.5690], [5.7092, 8.1585], and [0.5173, 2.5298], respectively, and S.E. = 0.1412, d.f. = 85..57

Fig. 13: Flower count data in three different sites in Eneabba. The mean differences between these three sites was used to create the habitat quality coefficient of mean increase in flower production. 58

Fig. 14: *Classification of our flower and cone count data under current climate conditions: (a) plants were classified into two poor and good producers using the 75th percentile. (b) Flower count distribution per plant per year with the binary plant performance classification. (c) Good producers held a significantly (Mann-Whitney test) higher number of cones, and (d) had higher survival rates than poor producers*...60

Fig. 15: Dependence of flower production under current climate conditions (2008–2017, plant age 11–20 years) on the total annual rainfall of previous year (i.e., lag annual rainfall), and sum of winter–spring rainfall of the previous three years using (A) the linear mixed-effect model assuming that the residual error have a Gaussian distribution, and (C) the generalized linear mixed-effect model for the negative binomial family. Model versions B and D use only the predictor variable lag annual rainfall with the same modeling techniques of A and C, respectively. The four formulas had as random intercepts “year” and “tag”. See statistical results in Table 16.......................................62

Fig. 16: Calibration of LDD of seeds by post-fire wind via inverse modeling using two empirical patterns: percentage of immigrants and number of population IDs per habitat patch. We assumed that the model reproduces the two patterns when the immigrant percentage matches the observed immigration rate with ±10% error (i.e., 4.95–7.48%) and the number of population IDs is equal to or greater than two (He et al., 2004, 2010)...70

1. Problem formulation

This TRACE element provides supporting information on: The decision-making context in which the model will be used; a precise specification of the question(s) that should be answered with the model, including a specification of necessary model outputs; and a statement of the domain of applicability of the model, including the extent of acceptable extrapolations.

Summary:

> **The MetaSqueeze model will be used by fire managers, conservationists, and scientists interested in climate change and fire effects on serotinous plants at the local and landscape scales. The model was designed under the Interval Squeeze framework (Enright et al., 2015). The model was implemented so that extrapolation to other species and landscapes is possible.**

Climate change is significantly impacting Mediterranean ecosystems, affecting species like the fire-killed, serotinous shrub *Banksia hookeriana* through increased temperatures, reduced rainfall, and altered fire regimes (Breshears et al., 2021; Flannigan et al., 2009; Grainger et al., 2022; Lozano et al., 2017; Timbal et al., 2006). The interval squeeze concept (Enright et al., 2015) suggests climate-driven demographic shifts and reduced post-fire recruitment, challenging species persistence. Despite these challenges, long-distance dispersal mechanisms, including post-fire wind dispersal and bird-mediated dispersal, along with spatially structured metapopulation dynamics, offer potential compensatory mechanisms against local extinction risks (Groeneveld et al., 2008; He et al., 2004, 2010).

Using the well studied fire-killed and serotinous shrub species *B. hookeriana* as a model species, we aim to better understand the potential role of metapopulation dynamics for mitigating the negative consequences of climatic changes for species in fire-prone landscapes. We extend a recent mechanistic, process-based, spatially implicit population model of *B. hookeriana* (Souto-Veiga et al., 2022) to a spatially explicit metapopulation model. We systematically test the effects of different ecological processes and assumptions on metapopulation dynamics under past (1988–2002) and current (2003–2017) climatic conditions. In particular, we focus on effects of different spatio-temporal fire scenarios as a driver of regional dynamics and the role of local populations as potential seed sources. For the latter we explore effects of (possibly) lower plant competition at earlier life stages under current conditions because of reduced plant densities, and effects of intraspecific variability in plant performance among and within patches. Overall, effects of different scenarios are quantified with regard to the effectiveness of recolonization events for metapopulation persistence.

2. Model description

This TRACE element provides supporting information on: The model. Provides a detailed written model description. For individual/agent-based and other simulation models, the ODD protocol is recommended as standard format. For complex submodels it should include concise explanations of the underlying rationale. Model users should learn what the model is, how it works, and what guided its design.

Summary:

Here we present the model description following the ODD (Overview, Design concepts and Details) protocol (Grimm et al., 2006, 2010, 2020). This model extends the previous population dynamics model (Souto-Veiga et al., 2022) by including subpopulations connected by long-distance dispersal processes, and spatially explicit patchy fires as main disturbance factor. The model, which was implemented in C++ 17, is open source and can be downloaded from https://github.com/rsoutoveiga/metasqueeze-model. Data analysis and visualization were performed using R v.3.5.3 (R Core Team 2019). We used the following R packages: 'ggplot2' v3.3.6 (Wickham, 2016), 'plotly' v4.10.0 (Sievert, 2020), 'dplyr' v1.0.9 (Wickham et al., 2022), 'fitdistrplus' v1.1.8 (Delignette-Muller & Dutang, 2015), 'plyr' v1.8.7 (Wickham, 2011), 'ggpbur' v0.4.0 (Kassambara, 2020), 'ggrepel' v0.9.1 (Slowikowski, 2021), 'reticulate' v1.26 (Ushey et al., 2022).

Section contents

2. Model description....10
2.1 Purpose and patterns....11
2.2 Entities, state variables, and scales....11
2.3 Process overview and scheduling....14
2.4 Design concepts....18

2.4.1 Basic principles....18
2.4.2 Interaction....19
2.4.3 Stochasticity....19
2.4.4 Observation....19
2.5 Initialization....20
2.6 Input data....21
2.7 Submodels....23
2.7.1 Weather conditions....23
2.7.3 Dispersal....25
2.7.3.1 LDD of cones by birds....25
2.7.3.2 LDD of seeds by post-fire wind....25
2.7.4 Inter-fire plant mortality....26
2.7.5 Aging of plants and cones....31
2.7.6 Density regulation of adult plants....31
2.7.7 Cone production and storage....31
2.7.7.1 Flower production using cohort-based approach....32
2.7.7.1 Flower production using plant individual-based approach....36
2.7.8 Viable seeds per cone age....41

2.1 Purpose and patterns

The overall purpose of the model is to investigate the role of metapopulation dynamics in mitigating the adverse effects of climate change on serotinous fire-killed species to support their conservation.

This model extends a previously published spatially-implicit population model based on the well-studied and highly serotinous *Banksia hookeriana* shrub (Souto-Veiga et al., 2022) into a spatially-explicit metapopulation. The main added parts are: (i) the explicit location of geographic populations (i.e. suitable patches), (ii) the processes of long-distance dispersal of cones and seeds, and (iii) the location of wildfires. The model is based on the concept of the Interval Squeeze (Enright et al., 2015). We compared the results under the initial climatic conditions (i.e., the reference conditions) with the current conditions.

The model's results were compared with empirical data on immigration to determine whether the model provides realistic and relevant patterns for the target (immigration rates and population ID per patch).

2.2 Entities, state variables, and scales

The model includes populations, age cohorts, and individual *B. hookeriana* plants. Populations correspond to the geographical populations of *B. hookeriana* located in suitable habitat patches on dune crests, which are defined as areas with deep sands that support the complete life cycle of the species, contrasting with the uninhabitable intervening depressions. They were characterized by their unique identifier, carrying capacity (i.e., the maximum number of adult plants per unit area that can be sustained), time since the last fire, habitat quality, list of cohorts, location of the dune in grid cells, and location of birds in grid cells during cone dispersal. A key aspect tracked for populations was their status, including whether they were extinct (defined as having no remaining individual plants, thus representing empty but suitable habitat patches), recolonized, or burnt. Cohorts were characterized by the age of the plants, the number of plants, whether the cohort originated from LDD (long-distance dispersal) seeds, the average size of the cone bank per plant—with cones classified by age (only for the cohort-based approach), and the list of individual plants (only for the individual-based approach). In addition to characterizing cohorts by attributes such as age, number of plants, and dispersal type, we implemented a triple identifier system for tracking the

development and spatial dynamics of these cohorts from seed dispersal to plant maturity across populations throughout the simulation. This system comprises three key identifiers: the ‘initial population ID,’ which is the unique identifier of the population where the seeds that initiated the cohort were dispersed; the ‘previous population ID,’ used to trace the source population of these seeds in the preceding generation and to track immigration rates; and the ‘current population ID,’ which reflects the population where these seeds have established and grown into plants.

By differentiating between ‘previous’ and ‘current’ population IDs, we can dynamically categorize cohorts originating from seed dispersal as either ‘residents’ or ‘immigrants’ based on their dispersal and establishment events across populations. For example, a cohort that is initiated by seeds dispersing from population ID 1 and establishing in population ID 2, transitioning from (1, 1, 1) to (1, 1, 2), is denoted as an immigrant in the new location. This methodical tracking facilitates a comprehensive investigation into the dynamics of population structure across the landscape. Individual plants were characterized by their cone bank in the canopy. The global environment describes the global variables at the system level: annual weather conditions, time since the last fire, fire interval, and simulation year.

The state variables for populations, cohorts, and plants are described in Tables 1–3.

A time step corresponds to one year, and the simulations were run for 500 years or until metapopulation extinction. The study area is a grid rectangle 3 km × 5 km in length (He et al., 2010), and the size of the grid cells is 100 m × 100 m (Esther et al., 2008).

Table 1: *Population state variables*

Symbol	Code	Variable type [units]	Description
id	id	Natural, static; [#].	Population identification number.
nr_{cells}	num_cells	Whole, static, [#].	Patch size.
K	carrying_capacity	Whole, static, [#].	Maximum number of adult plants that the population can hold.
$cell_{loc}$	cell_locations	One-dimensional vector of whole numbers,	Cell locations of the population.

Symbol	Code	Variable type [units]	Description
		static.	
tsf	time_since_fire	Whole, dynamic [years].	Number of years since the last fire.
is_{ext}	is_extinct	Boolean, dynamic.	Population status whether extinct or not.
is_{recol}	is_recolonized	Boolean, dynamic.	Population status whether recolonized or not.
is_{fire}	is_fire	Boolean, dynamic.	Population status whether it is covered by fire or not.
coh_{list}	cohort_list	List of cohort entities, dynamic.	List of cohort entities.
habitat	habitat_quality	String, static.	Habitat quality (low, moderate, high).

Table 2: *Cohort state variables.*

Symbol	Code	Variable type [units]	Description
age	age	Whole, static; [#].	Age of the cohort.
nr_{plants}	num_plants	Whole, static, [#].	Number of plant individuals.
$bank_{coh}$	conebank	One-dimensional vector of whole numbers, dynamic.	Average size of the canopy conebank per plant individual and age class of the cone. The size of the vector is defined by the sum of the plant parameters *cone_cycle* and *seed_longevity.*
is_{LDD}	is_LDD	Boolean, static.	Classifies a cohort between LDD (true) and SDD (false) cohort.
plt_{list}	plants_list	List of plant entities, dynamic.	List of plant entities.

Table 3: *Plant state variable.*

Symbol	Code	Variable type [units]	Description
$bank_{plt}$	conebank	One-dimensional vector of whole numbers, dynamic.	Average size of the canopy conebank per plant individual and age class of the cone. The size of the vector is defined by the sum of the plant parameters *cone_cycle* and *seed_longevity.*

2.3 Process overview and scheduling

Process overview: The most important processes of the model are presented in Fig. 1. The model started with the initialization of the experiment, checking the validity of the input parameters values, and calculation of some derived parameters from input parameters such as available viable seeds per cone age. Then the climate data were read in, which contained three predictor variables formed from the real rainfall conditions of the weather station closest to the study area (Eneabba Weather station nr 08225; Australian Bureau of Meteorology): lag winter–spring rainfall, lag annual rainfall, and sum winter–spring rainfall of the last three years. These rainfall predictor variables were used in flower production and inter-fire plant mortality processes (Souto-Veiga et al., 2022). Next, the study area replicate was generated. The dunes were randomly located in the study area using a random walk algorithm, imitating the mosaic of irregular shape dunes of the Eneabba Sandplain landscape (He et al., 2010). The minimum distance between dunes was 100 m (He et al., 2010). Then, all experiments start with the first simulation year burning all dunes (i.e., occupied and unoccupied habitat patches), in which all plants die and disperse their viable seeds.

After the first year of simulation, the yearly life cycle of the *B. hookeriana* metapopulation continued until either the maximum number of simulated years was reached (500 years), or the metapopulation went extinct. Each year, which started at the end of the fire season (approx. April), began (1) by picking the weather conditions at random from the climate input data, followed by (2) the evaluation of whether there was a fire event. If a fire occurred, the fire spread formula of Groeneveld et al. (2008) was used to assess whether each population is burnt or not. In case of a local fire, all plants of the population died, and the number of available fertile cones and viable seeds were calculated. Then, the evaluation of recolonization and immigration-caused population growth effects was performed for each plant stage. The following dispersal process included (in the given order) the LDD of cones by birds and the LDD of viable seeds by post-fire wind. The remaining viable seeds stayed within the source population. In contrast, if the population was unburned, the following processes were executed in the given order: inter-fire plant mortality, aging of plants and cones, density regulation of adult plants, and cone production and storage. At the end of the year, the current state of the metapopulation was evaluated, summarizing various output metrics such as the number of recolonization and immigration-caused population growth effects, the number of extinct populations, and other more specific metrics which depend on the experiment type being executed.

The fate of the individual plants depended on the probability of plant mortality between fires or by crown fires, in which case all plants died (Enright et al., 1998). Adult plants (≥ five years old; Enright et al., 1998) produce new cones when weather conditions werere favorable, thus increasing the canopy cone bank.

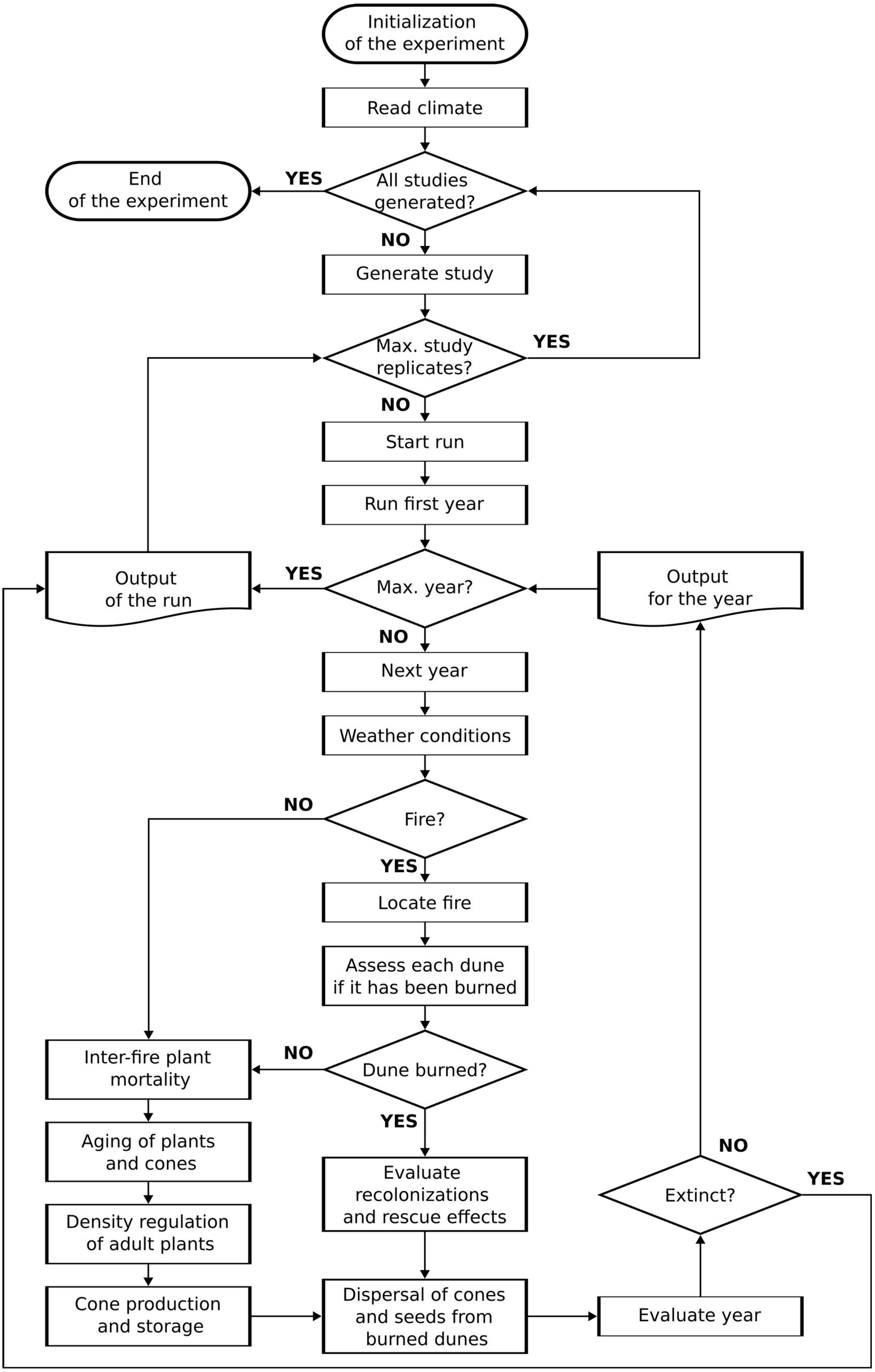

Fig. 1: *Key processes of the metapopulation model of B. hookeriana. Detailed description in the main text.*

Schedule: Following it is presented the model schedule at each time step. The populations executed their actions from lowest to highest population ID. The cohorts executed the actions in order of highest to lowest value of their state variable age, i.e. oldest to youngest. The plants execution order was trivial:

1. The global environment updates its *weather_conditions* variable by picking a year randomly from the selected climate scenario.

2. Global environment increments its *time_since_fire* variable by one year.

3. Populations increment their *time_since_fire* variable by one year

4. Fire event is evaluated by comparing the metapopulations variables *time_since_fire* and *fire_interval*.

4.1 If fire occurs, i.e. *time_since_fire* is equal to *fire_interval*, then:

4.1.1 Global environment updates its *fire_interval* variable based on the selected fire scenario (i.e., deterministic or stochastic).

4.1.2 Global environment sets its *time_since_fire* variable to zero.

4.1.3 Fire patch is located randomly on the landscape.

4.1.4. Each population is assessed whether it is burned or not. Burned populations evaluate recolonizations and immigration-caused population growth effects state.

4.2 If population was not burned:

4.2.1 The cohorts each executes their “Inter-fire plant mortality” submodel, in which updates the cohort variable *num_individuals* subtracting the calculated number of dead plants.

4.2.2 The cohorts (or plants when the individual-approach is performed) execute the “aging of plants and seeds” submodel, in which their state variable *age* increases to one and the elements of the conebank vector are shifted one unit to the right.

4.2.3 The cohort entities which are at the plant age maturity executes the “density regulation of adult plants” submodel, and it updates its variable *num_individuals*.

4.2.4 The population entity executes the “remove empty cohorts” submodel, in which it removes a cohort if its variable *num_individuals* is at zero.

4.2.5 All mature cohorts (or plants when the individual-approach is performed) execute “cone production and storage” submodel, in which the new produced cones are assigned to the first element of the vector of the state variable *conebank.*

5. Burned populations execute dispersal submodels.

6. At the end of the year, the current state of the metapopulation is evaluated, summarizing various output metrics such as the number of recolonization and immigration-caused population growth effects, the number of extinct populations, and other more specific metrics which depend on the experiment type being executed.

2.4 Design concepts

We describe only here the ODD elements that apply to our model, i.e., basic principles, interaction, stochasticity, and observation.

2.4.1 Basic principles

Our metapopulation model follows the Interval Squeeze conceptual framework proposed by Enright et al. (2015). The Interval Squeeze concept postulates that changes in climate conditions in the direction of more frequent and intense droughts in combination with more frequent fires will decrease the population persistence of fire-killed, serotinous woody plants in fire-prone ecosystems. Weather conditions directly impact the production of flowers of adult plants, mortality rates of adult plants (demographic shift), and the recruitment of seedlings after a fire (post-recruitment shift). When a fire occurs, all plants die, and all seeds are released from serotinous cones. At a local scale,

a population may become extinct if a fire occurs either before plants reach maturity or if plants do not hold enough seeds in their canopy seed bank.

However, LDD may connect local populations to form a metapopulation in which local extinctions may be prevented or compensated by recolonization events (Groeneveld et al., 2008; He et al., 2004, 2010). The positive effect of spatially structured metapopulation dynamics generally depends on recolonization events that facilitate regional survival despite local population extinction (Hanski, 1998).

2.4.2 Interaction

There is no direct interaction between cohorts and plants. An indirect interaction between cohorts and plants occurs in the sub-model "Density regulation of adult plants", where carrying capacity is applied.

Since the possible relationships between weather conditions and fires are not sufficiently clear, both environmental variables are independent. This independence allows the investigation of a broader range of scenarios.

2.4.3 Stochasticity

All stochastic processes use the pseudo-random generator Mersenne Twister (Matsumoto & Nishimura, 1998). Inter-fire plant mortality, flower production in the individual-based approach (see submodel section), weather conditions, and location of the patchy fire involve stochastic events. Also, the location and shape of the habitat patches (i.e., populations) were randomly located to generalize the results in the context of landscape configuration and reduce artifacts.

2.4.4 Observation

All variables were recorded yearly. Cohort entities recorded the number of individuals and cones per individual. Population updated whether it is extinct or recolonized. Furthermore, recolonization

and immigration-caused population growth effects are updated yearly. Both are disentangled into four plant stages:

- seed (viable seeds that fall within a suitable patch)
- seedling (1-year-old plant)
- adult (5-year-old plant, i.e., maturity age), and
- source (adult plant with canopy seed bank that reaches subsequent fire)

Each population tracked the LDD of seed by wind and bird and their location. Four locations were tracked for LDD seeds by wind: outside the study area, unsuitable, immigrant, and resident. Two locations were tracked for LDD of cones by cockatoos: immigrant and resident.

The global environment recorded the persistence of the metapopulation, and populations recorded their persistence and the number of generations. The global environment recorded the total of resident and immigrant plants in the metapopulation. The simulation runtime was 500 years or until the metapopulation became extinct.

2.5 Initialization

The landscape was generated randomly: The habitat patches (i.e., populations or dunes) were read from an input file with the following data: population id, the initial number of individuals, and habitat quality (low, moderate, high). First, the file was read, then the list was randomly reorganized. Then, habitat patches were located randomly using a random walk algorithm.

The initial plants per initially occupied population and the percentage of LDD of seeds parameters required further calibration using pattern-oriented modeling (Railsback & Grimm, 2019). Initial plants in each population were adjusted for each mortality scenario tested by reaching the same 8-year-old population density observed in He et al. (2010).

The study area covered a 3 km × 5 km area of Eneabba Sandplain in South-West Australia (He et al., 2010), comprised of 100 m × 100 m grid cells. One mature plant was assumed to require an area of 2 m × 2 m (Esther et al., 2008), i.e., the carrying capacity used in the process of density

regulation of adult plants. The metapopulation contained 37 geographical populations (i.e., dunes or habitat patches) defined by the population ID, area size, initial individuals in the simulation, and habitat quality. The metapopulation consisted of 18 initially occupied dunes and 17 unoccupied dunes. The dunes were randomly located in the study area using a random walk algorithm, imitating the mosaic of irregular shape dunes of the Eneabba Sandplain landscape (He et al., 2010). The minimum distance between dunes was 100 m (He et al., 2010). Then, all experiments start with the first simulation year burning all dunes (i.e., occupied and unoccupied habitat patches), in which all plants die and disperse their viable seeds.

The main results of the paper, the study area (landscape), was replicated 30 times to generalize the results in the context of landscape configuration and reduce artifacts. All simulation experiments took the same seeds for the random generator engine (from 1 to 30) to have the same random study area replicated among simulation experiments and take the landscape configuration effects out of the equation when comparing among scenarios.

2.6 Input data

The climate is the only input data to represent time-varying processes. The climate file contains real rainfall conditions taken from the closest weather station to the study area, i.e., Eneabba Weather Station no. 8225 (*Australia's Official Weather Forecasts & Weather Radar - Bureau of Meteorology*, n.d.). Two climate scenarios were compared and tested systematically: baseline (1988–2002) and current (2003–2017). Keith et al. (2014) found that antecedent rainfall conditions are strongly correlated with flower production and plant mortality. Each climate year is composed of a set of three predictor variables driving the annual flower production and plant mortality: total winter–spring rainfall of the previous year, total annual rainfall of the previous year, and total winter–spring rainfall of the previous three years (all variables in mm). See the submodels “flower production” and “inter-fire plant mortality” for further information. Fig. 2 shows the distribution of rainfall for both climate scenarios for each predictor variable. Each predictor decreased on average by approx. 15%.

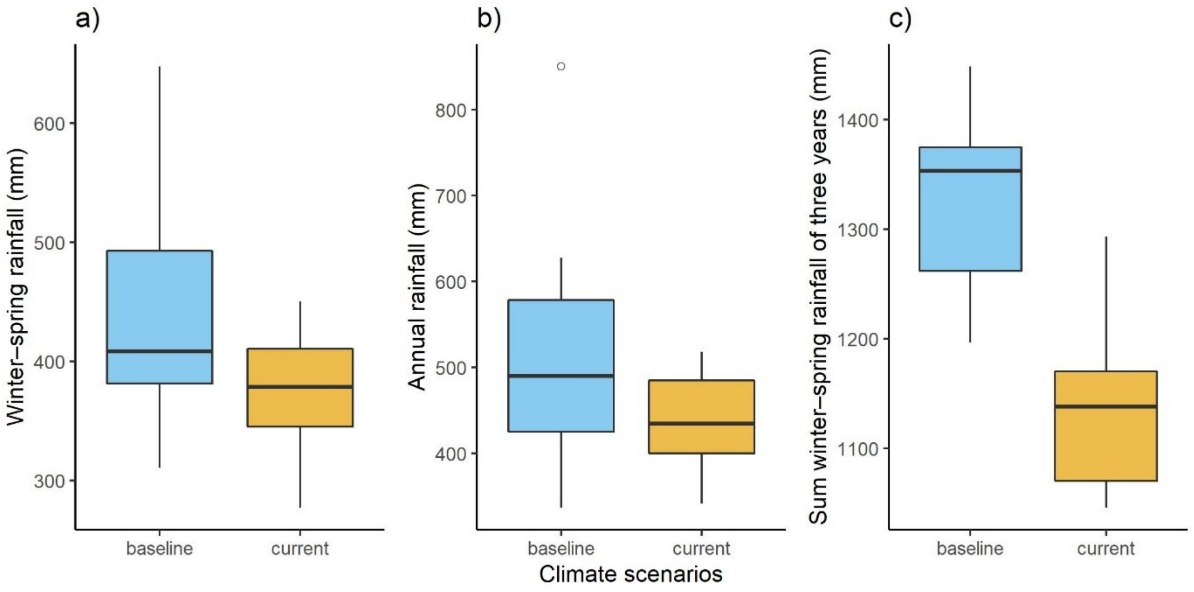

Fig. 2: *The climate scenarios used in the simulation experiments were baseline (1988–2002) and current (2003–2017). Three predictor variables were used in the model: a) winter–spring rainfall of the previous year, b) annual rainfall of the previous year, and c) sum of winter–spring rainfall of the last three years. Each predictor decreased on average by approx. 15%. The rainfall data were retrieved from the closest weather station to the study area, i.e., Eneabba Weather Station no. 8225.*

2.7 Submodels

The model's parameters are summarized in Section 3 (Data evaluation).

2.7.1 Weather conditions

In each time step a climate year set is randomly picked from the selected climate scenario (see 2.6 Input data). All climate year sets have an equal probability to be selected. The selected climate year is stored in the variable weather_conditions of the global environment.

2.7.2 Fire events

The fire was implemented in three main steps:

1. Evaluation of whether there is a fire event.
2. The spatial location of the fire area in the landscape matrix.
3. Evaluate whether a habitat patch is burned.

Fire event is evaluated every time step (i.e., every year) and checks whether fire occurs by comparing the two state variables *time_since_fire* and *fire_interval* of the global environment. A fire event occurs if both variables have the same value, i.e., the same year. Otherwise, fire does not occur.

When a fire event occurs, *time_since_fire* is set to zero, and *fire_interval* is updated from the selected fire event scenario: deterministic and stochastic. For deterministic fire events, each run simulation has a fixed fire interval, and the model parameter *fire_interval_mean* defines its value. For stochastic fire events, the time between two consecutive fires is determined randomly. Although the model could simulate stochastic fires (normal distribution and Weibull distribution), we used only deterministic fire events in this study. Fire interval cannot be lower than the parameter *fire_interval_lower_cut*.

To locate the fire area in the landscape matrix, we chose the ellipse shape as it is the most used fire shape in ideal conditions (Glasa & Halada, 2008; Green, 1983), and most fires in the region are

wind-driven with elliptical shapes (Enright et al., 2012). We defined as ‘baseline’ patchy fire size the circumscribed ellipse, i.e., the ellipse passing through all vertices of the study area boundaries (Fig. 3a). Patchy fire events were randomly located given the center grid cell, the size (x and y semi-axes), and the angle orientation (Fig. 3b).

A population was assessed as either completely burned or not burned at all, based on any degree of overlap with the patchy fire ellipse, no matter how minimal (Fig. 3c). This initial assessment was further refined by considering the population's fuel load, which was determined using the fire spread formula of Groeneveld et al. (2008) A critical factor in this assessment was the time elapsed since the population's last fire, as it is a derivative of fuel load; a greater time since last fire signifies more vegetation, thereby increasing the fuel load. When the time since the last fire in a population or 'patch' exceeded 12 years, the accumulated fuel load was considered sufficient to carry a fire, hence the population was always burned (i.e., there was a 100% probability of the population being burned if an ignition occurred). A minimum interval of three years post-fire was needed for a population to become eligible for burn assessment. Between three and twelve years, the probability of a population burning increased linearly based on our established assumptions and the fire spread formula. Fig. 3d shows the distribution of burned suitable areas for each fire size tested.

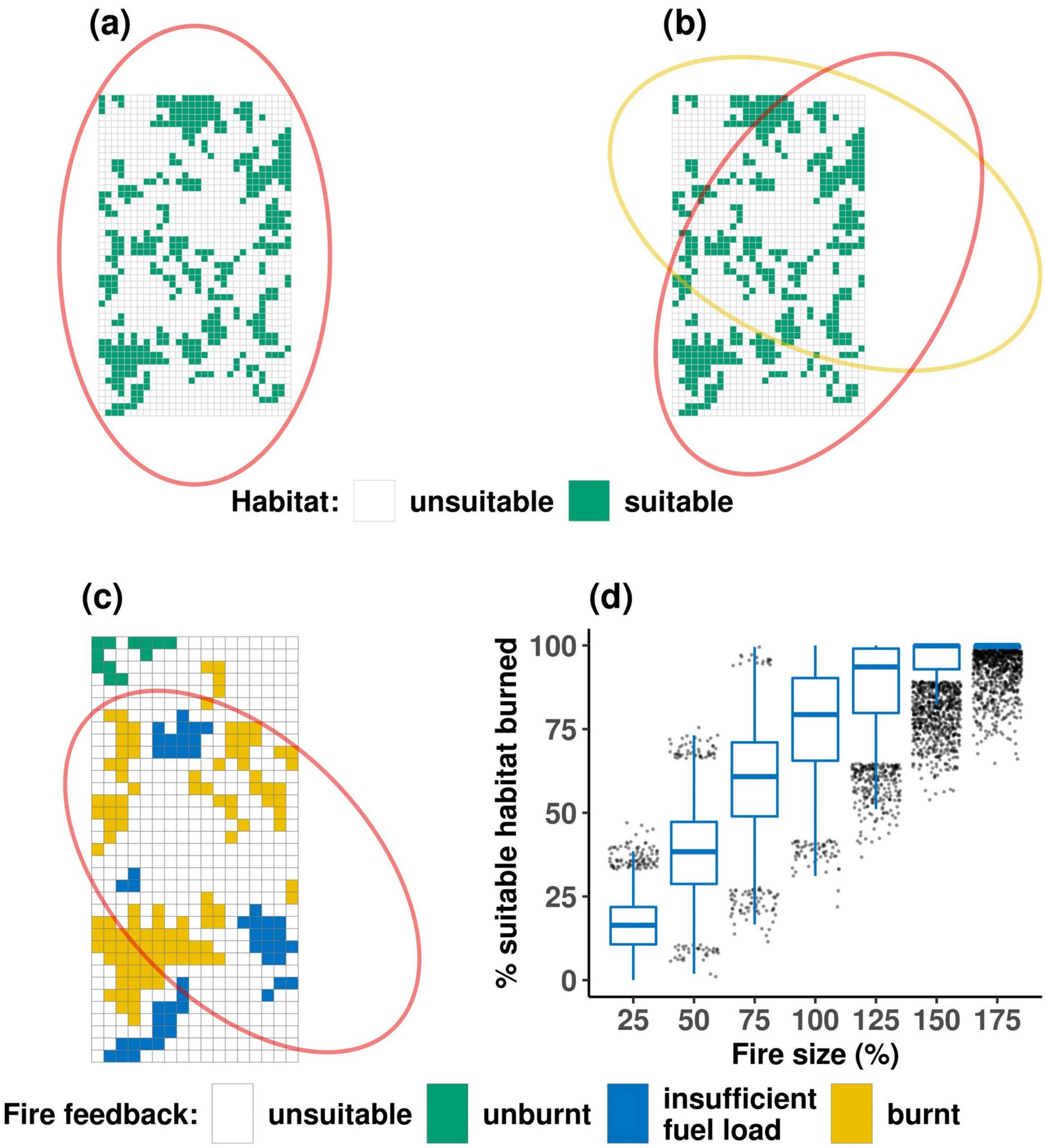

Fig. 3: *Demonstration of patchy fire events. (a) the circumscribed ellipse represents the baseline fire size (100%). (b) two 100% fire-size events with different center point positions and orientations. (c) possible population feedback to patchy fires: unburnt (green) because it is outside of the fire ellipse, unburnt due to insufficient fuel load (blue), and burnt (orange) population from being partly overlapped or fully covered by the fire ellipse and with enough fuel load. The boxplot in (d) shows the burnt suitable area distribution for all fire sizes tested in the study.*

2.7.4 Dispersal

Two long-distance dispersal (LDD) mechanisms were presented in the following order: LDD of cones by birds and LDD of seeds by post-fire wind. The remaining seeds stay in the source population (He et al., 2004).

2.7.4.1 LDD of cones by birds

We considered the dispersal of cones as direct dispersal; that is, birds dispersed a cone within the same population or among populations. The calculation of the number of seeds available for dispersal within each cone before birds disperse them was as follows: first, the number of follicles was drawn randomly from the fitted density curve for the baseline or current climate scenario (Fig. 9, Section 3). The best fit for the baseline scenario was the negative binomial distribution (size = 6.22, and mu = 10.08), and for the current scenario was the Poisson distribution (lambda = 7.33). Then, the number of viable seeds in each cone is calculated for 1-year-old cones, because cockatoos were most likely to remove recently produced cones that are located on the growing outer edges of the canopy. The remaining viable seeds for each dispersed cone were calculated by picking a random proportion of open follicles from 0.5 to 1 from a uniform distribution because 50% of the follicles were open two hours after a fire (Enright & Lamont, 1989). The cone with the remaining viable seeds is finally dispersed by randomly picking one of the grid cells within the maximum distance dispersal observed (1250 m) (He et al., 2004).

2.7.4.2 LDD of seeds by post-fire wind

Viable seeds can be dispersed within the source population (resident), in another population (immigrant), and in unsuitable areas or outside the study area (lost seed). Any viable seed to be dispersed by LDD through post-fire wind went through the following steps: First, the starting point of dispersal was randomly selected within the population. Then, a random angle was selected from a uniform distribution since no pattern was observed in the data (He et al., 2010). Then, a random distance was chosen from the dispersal kernel, which was fitted to the observed immigrants (He et al., 2010). The best fit was a log-normal distribution (meanlog = 6.79, sdlog = 0.68) (Fig. 8, Section 3). Finally, the drop point was calculated using the Euclidean distance formula considering the calculated origin point, angle, and distance.

2.7.5 Inter-fire plant mortality

The "inter-fire plant mortality" submodel is a partly stochastic process. The stochasticity was done by comparing a number chosen at random, r, from a uniform distribution between 0 and 1, r $\in$ $U[0, 1]$, with the calculated probability of inter-fire plant mortality: If $r \leq$ P(*mortality*), then the plant dies. Otherwise, if $r >$ P(*mortaltity*), then the plant survives. When a plant dies during the inter-fire period, all seeds within closed cones decay and lose viability (Enright et al., 1996).

For the sake of computation performance, stochasticity of plant mortality was done individually for each plant within a cohort only when the total number of individuals in that cohort was equal to or lower than the population's carrying capacity. When the number of individuals in a cohort is greater than the carrying capacity, the total number of dead plants is randomly picked from a binomial distribution.

Using a plant mortality rate dataset, we tested five inter-fire plant mortality scenarios based on different ecological assumptions (Fig. 4): (i) age-weather relative impacts (Souto-Veiga et al., 2022), (ii) mean age-weather absolute impacts, (iii) LDD cohorts have lower mortality than SDD (short-distance dispersal) cohorts, (iv) immigrant cohorts have lower mortality than resident cohorts, and (v) lower intraspecific competition at earlier life stages under current climate conditions.

The P(*mortality*) of the scenario "age-weather relative impacts" (Fig. 4a) was calculated following a two-step hierarchical approach. First, the mortality P was calculated based on plant age (Eqns. 1 and 2), and then weather conditions modified the mortality P (Eqns. 3 and 4). The mortality probabilities based on plant age were constituted in three age segments (Eqn. 2): i) when plant age was zero (i.e., mortality of post-fire recruits), the probability was 0.923 (mean between the spring- and autumn-burned areas (Enright & Lamont, 1989), ii) for plant ages 1–25 years, the mortality probability curve was taken from the age-standardized quadratic model with negative exponents of mean mortality rates from Keith et al. (2014), where the age of 15 years was assumed as the lowest age-related mortality rate, and thereafter, iii) the mortality probability increased by 0.01 steps each year due to plant senescence, $sens_{incr}$ (Enright et al., 1998). Keith et al. (2014) found a robust linear relationship between the variation around the mean mortality rate of the age-standardized quadratic model and the total winter–spring rainfall from the previous year (Eqn. 3). This linear equation

impacts plant mortality based on age with a slope of approx. 6% per 100 mm around the long-term average winter–spring rainfall impacts the probability of plant mortality based on age, as shown in Eqn. 4.

$$M(age) = mort_a + \frac{mort_b}{age} + \frac{mort_c}{age^2} \tag{1}$$

where,

$mort_a$ = 0.05553,

$mort_b$ = 0.2645 year^{-1}, and

$mort_c$ = 0.179 years^{-2}

$$P_{mortalityAge} \begin{cases} 0.969, & age = 0 \\ M(age), & 1 \geq age \leq adult_{age} \\ M(adult_{age}), & adult_{age} \geq age \leq sen_{age} \\ M(age) + sen_{incr} * (age - sen_{age}), & age > sen_{incr} \end{cases} \tag{2}$$

where,

$adult_{age}$ 15 years

sen_{age} = 25 years

sen_{incr} = 0.01

$$Mortality_{weather} = mort_d * rainfall_{WinterSpring} + mort_e \quad (3)$$

where,

$mort_d$ = –0.00061 mm^{-1}, and

$mort_e$ = 0.26873

$$P_{mortalityAgeWeather} = P_{mortalityAge} * \left(1 + Mortality_{weather}\right) \quad (4)$$

The resultant age-weather plant mortality probability of the post-fire recruits (i.e., plants at age zero) was truncated so that the probabilities cannot be less than 0.911 (i.e., the minimum mortality found within a spring-burned area, Enright and Lamont 1989) or more than 0.987 (Groeneveld et al., 2002). In contrast, recruits from seeds dispersed during the period between fires have a higher probability of mortality due to competition with already-established vegetation.

In the "mean age-weather absolute impacts" scenario (Fig. 4b), P(mortality) was calculated by taking the mean of the two linear regression equations of each mortality rate dataset (spring and autumn dataset, Eqns. 5 and 6, respectively) with plant age and winter–spring rainfall explanatory variables.

$$Spring = \frac{mort_a}{age} + mort_b * weather_{WinterSpring} + mort_c \quad (5)$$

where,

$mort_a$ = 0.29 years,

$mort_b$ = –0.0005888 mm^{-1}, and

$mort_c$ = 0.32

$$Autumn = \frac{mort_d}{age} + mort_e * weather_{WinterSpring} + mort_f \tag{6}$$

where,

$mort_d$ = 0.65 years,

$mort_e$ = –0.0006502 mm^{-1}, and

$mort_f$ = 0.29

For the "LDD cohorts have lower mortality than SDD cohorts", LDD cohorts were assigned the equation with lower mortality (Eqn. 5) and SDD cohorts with higher mortality (Eqn. 6). For the "immigrant cohorts have lower mortality than resident cohorts", immigrant cohorts were assigned the equation with lower mortality (Eqn. 5) and resident cohorts with higher mortality (Eqn. 6). Finally, for the "lower intraspecific competition at earlier life stages under current climate conditions", all cohorts were assigned the equation with lower mortality (Eqn. 5 and Fig. 4c).

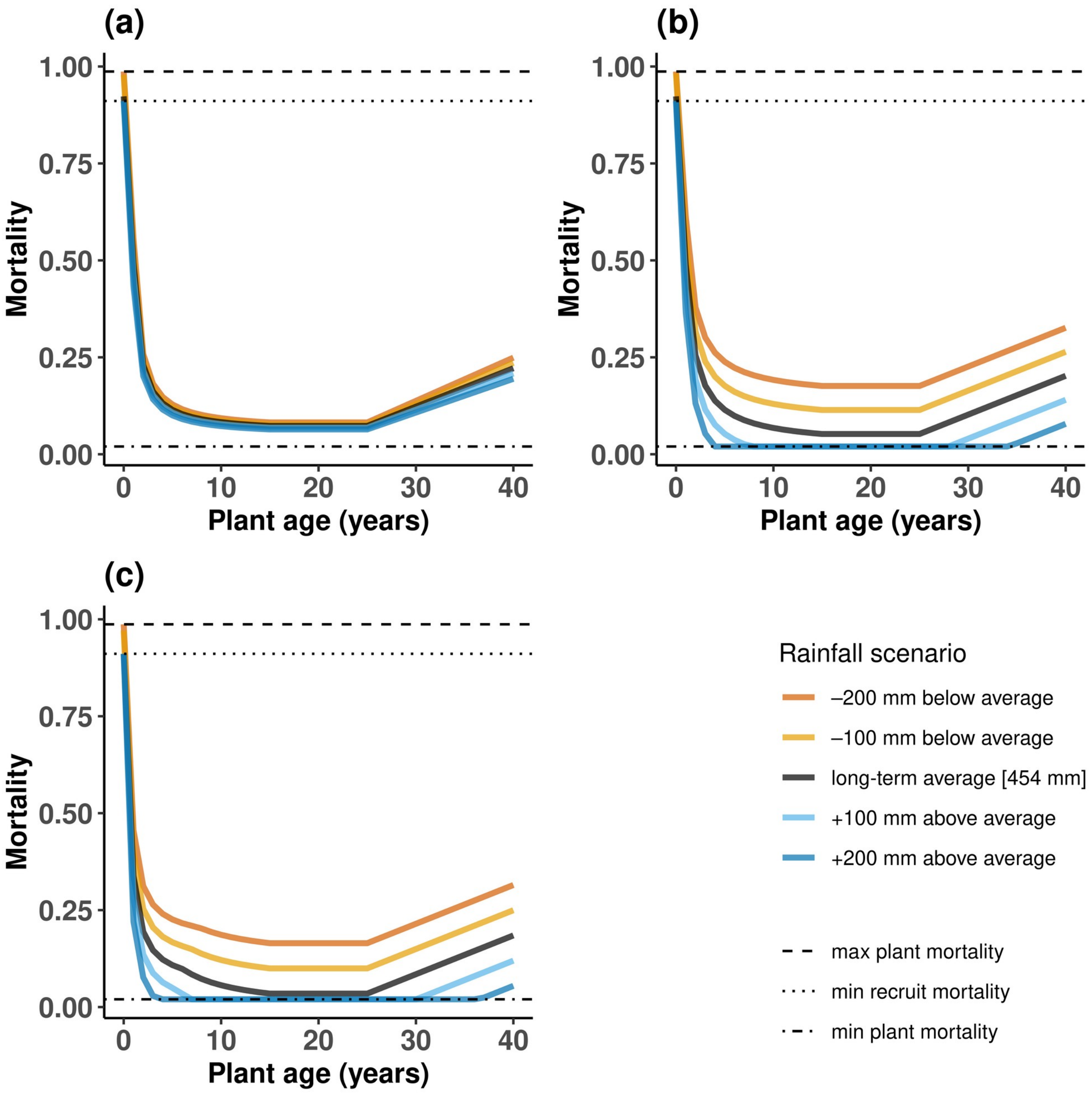

Fig. 4: *Annual mortality probability curve between fires by the age of the B. hookeriana shrub in Eneabba in different rainfall scenarios. Three mortality scenarios are shown: (a) age-weather relative impacts, (b) mean age-weather absolute impacts, and (c) always lowest mortality of age-weather absolute impact (lower intraspecific competition at earlier life stages under current climate conditions).*

2.7.6 Aging of plants and cones

All plants and cones stored in their canopy get one year older. The state variable *age* increases to one, and the elements of the one-dimensional vector *conebank* state variable are shifted one step to the right. The *conebank* vector size (i.e., number of elements) was defined by *cone_cycle* and *seed_longevity*. The *cone_cycle* refers to the time required for cones to reach maturity after successfully pollinating the flowers. We set the parameters *cone_cycle* to one year and the maximum seed longevity to 12 years (Enright et al., 1996),forming the *conebank* vector with 13 elements: The first element refers to the *cone_cycle*, in which the newly produced cones in the submodel “cone production and storage” were stored, and the rest of the elements refer to the seed ages from one to 12 years old.

2.7.7 Density regulation of adult plants

Plants start to produce fertile cones (i.e., reach maturity) at the age of five years (Enright et al., 1996). When plants reached maturity, the carrying capacity was applied to these new plants. A young plant only establishes successfully if the current number of mature plants is lower than the carrying capacity of the population. Otherwise, the plant dies. All new mature plants had the same probability of establishing as a young adult plants.

2.7.8 Cone production and storage

The cone production and storage submodel was executed once plants were five years old or older because few individuals are mature at a younger age (Enright et al., 1996). Cone production and storage consisted of two sub-submodels, executed in the following order: flower production and fertilization (i.e., percentage of fertile cones).

Adult plants (≥ five years old; Enright et al., 1998) produce new cones when weather conditions are favorable, thus increasing the canopy cone bank. There were two approaches to cone production and storage: The first used the age-weather function in Souto-Veiga et al. (2022) to calculate the mean flower production of all plants within cohorts, and the second was implemented here for the simulation experiments that distinguish plants within cohorts. When using plant entities, the number of flowers produced per plant each year was determined from fitted density curves. After calculating the flowers produced, the coefficient of mean increase in flower production due to dune habitat quality was applied. Each dune was assigned one of three identified static habitat qualities: low, moderate, and high (Fig. 13, Section 3). Finally, a percentage of flowers were converted into fertile cones (90% under baseline conditions, and 65% under current conditions).

There were two model approaches of flower production: i) cohort-based and ii) individual-based approach. The cohort-based calculation of the mean flower production per plant was based on regression analysis, and the individual-based approach drew the number of flowers per plant using probability density functions. Below, the two approaches are explained in detail.

2.7.8.1 Flower production using cohort-based approach

Flower production follows a similar two-step hierarchical approach as for the inter-fire plant mortality submodel: (1) flower production is based on the age-standardized logistic curve, and (2) antecedent weather conditions modify the age-based flower production.

For the implementation of flower production, two flower counts per individual were used: baseline data set, 1988–2002 (plant ages 17 to 31 years), and the current data set, 2008–2017 (plant ages 11 to 20 years). For the baseline dataset, Enright (Keith et al., 2014) found a robust linear relationship between mean annual flower production and the total winter–spring rainfall of the previous year (Eqn. 10). In contrast, in our new current data, we found two closely related but slightly more significant covariates: total annual rainfall of the previous year and the sum of winter–spring rainfall of the last three years. We fit the current flower data set using linear mixed-effect models (Eqn. 11). For more details on the regression analysis, see Section 3. Concerning flower production based on plant age, we adapted equation 1 of Enright et al. (1996) to a logistic growth curve (Eqn. 8), with the maximum value of the curve (approx. nine flowers per year) reached at age 15 years (Enright et al., 1996). The value of nine flowers was calculated from the best-fit linear relationship

in the baseline flower count data set (Eqn. 10) with the predictor variable under long-term average winter–spring rainfall conditions (average total winter–spring rainfall in 1965–1990, i.e., 454 mm).

This logistic growth curve of flower production based on plant age is then modified by multiplying the weather coefficient (Enq. 7). This coefficient is a linear division ratio between the flower production under specific rainfall conditions (Enq. 9) and the flower production under long-term average weather conditions in the baseline data set (i.e., approx. nine flowers). Fig. 5 shows the flower production curves in five different rainfall scenarios applying Eqn. 7 for both climate scenarios.

$$Flower\ production = Flower_{Age} * Weather_{coeff} \tag{7}$$

$$Flower_{age} = \frac{a}{\exp\left(c * \left(b - age\right)\right)} \tag{8}$$

where,

a = $flower_a$ = 9.179382 [# of flowers],

b = $flower_b$ = 8.710231 years, and

c = $flower_c$ = 0.6208263 $years^{-1}$

$$Weather_{coefficient} = \frac{Flower_{scenario}}{Flower_{baseline}(longterm_{rainfall})} \tag{9}$$

$$Flower_{baseline} = flr_a * rainfall_{WinterSpring} + flr_b \tag{10}$$

where,

flr_a = 0.053176 mm^{-1}, and

flr_b = –15.2124

$$Flower_{current} = flr_c * rainfall_{Annual} + flr_d * rainfall_{WinterSpring} + flr_e \tag{11}$$

where,

flr_c = 0.022191 mm^{-1},

flr_d = 0.023055 mm^{-1}, and

flr_e = –33.344613

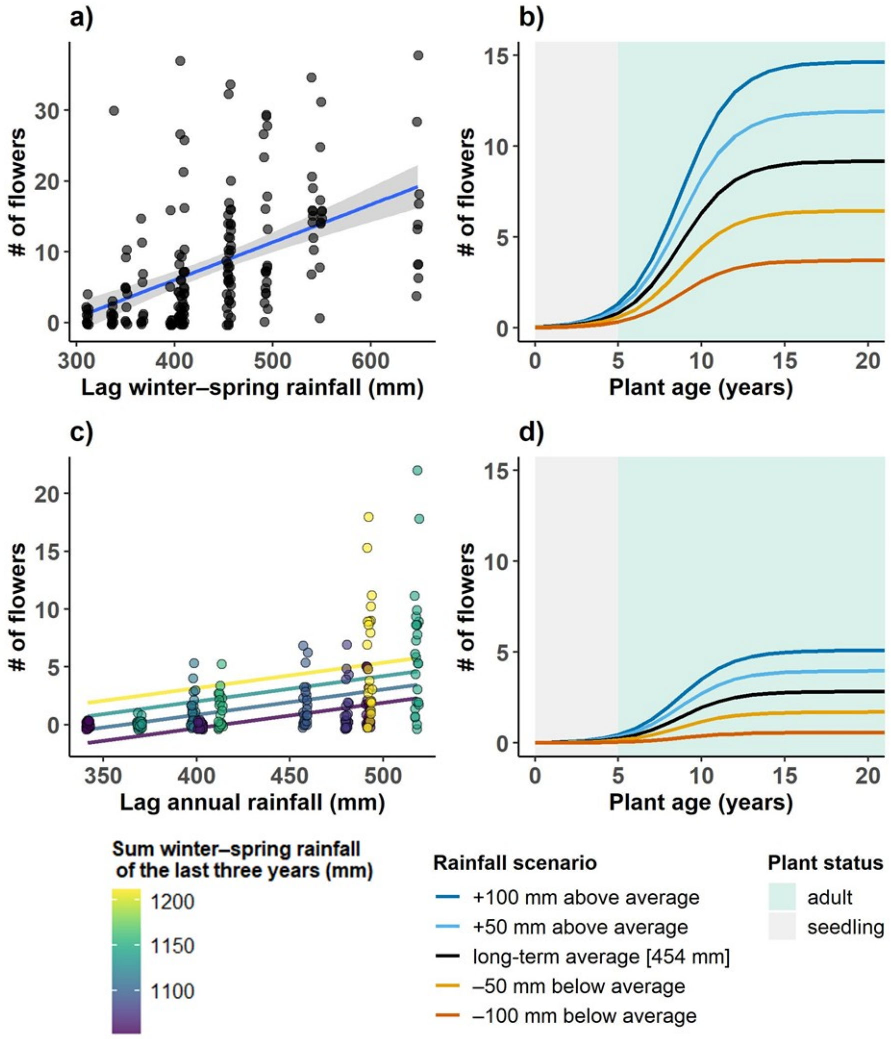

Fig. 5: *The two flower production scenarios used in the simulation experiments. Each row corresponds to a scenario: on the left side the selected model fit of the relationship between annual flower production per adult B. hookeriana plant and rainfall prediction variables is shown, and on the right side the impacts of the left best-fit model to the age-standardized flower production model, which corresponds to the black line in Fig. 4b. The first row shows the best linear mixed-effect model between the flower count baseline data set (1988–2002, plant age 17–31 yeas) and the total rainfall in winter–spring of the previous year (i.e., lag winter–spring). The second row shows the best linear mixed-model between current flower count data set (2008–2017, plant age 11–20 years) and*

2.7.8.1 Flower production using plant individual-based approach

The individual-based approach of flower production was used in the plant entity to include intraspecific variability in plant performance within dunes. Flower count data were classified into "poor" and "good" producers (see Section 3, Data evaluation). With this binary classification in plant performance, we classified the flower count data using the two predictor variables found in Souto-Veiga et al. (2022), i.e., the sum of the winter–spring rainfall of the last three years (*rain1*) and the previous annual rainfall (*rain2*), into different membership functions as done by the fuzzification stage of the fuzzy logic theory (Zadeh, 1988) (Fig. 6 and Tables 4–6). We constructed the membership functions using triangle and trapezoid shapes. The grade of the membership (y-axis) ranges from zero to one and represents the proportion. Then, for each membership function and plant performance class (i.e., poor and good producers), we selected the best fit (lowest AIC value) from three of the most common discrete probability density functions: Poisson, negative binomial, and geometric distribution (Table 4, and Fig. 7).

The flower production calculation of one plant using this individual-based approach is as follows: First, the fuzzy set using the predictor variable *rain1*, i.e., *fuzzy_set_1* (Fig. 6a and Table 4), had two membership functions. Each membership function corresponded to another fuzzy set with *rain2* predictor, i.e., fuzzy set for the "low" (Fig. 6b and Table 5), and "high" membership function (Fig. 6c and Table 6), i.e., *fuzzy_set_low* and *fuzzy_set_high*, respectively. Then, from these two fuzzy sets, the membership function values were calculated: The triangular membership function has three vertices, a, b, and c (from left to right), and the trapezoidal membership function has four vertices, a, b, c, and d (from left to right). For the calculation of the membership function for a given predictor value (i.e., *rain2*), used the Eqn. 12 for the triangular membership function, and Eqn. 13 for the trapezoidal membership function. Finally, the flower production was calculated by picking a random number from the best-fitted probability distribution in those membership functions with values greater than zero (see Fig. 7).

For example, if the weather conditions are 1200 mm for *rain1*, and 525 mm for *rain2*, then the flower count would be as follows: in *Fuzzy_set_1* (Fig. 6a), the proportion of the "low" membership function is zero, and the proportion of "high" is one. Thus the calculation of flower count comes only from the *fuzzy_set_high* (Fig. 6c). In the *fuzzy_set_high*, the 525 mm *rain2* predictor variable

is entirely within the "high" membership function. Therefore, the number of flowers is picked from the distributions in Fig. 7f.

Another example would be when the rainfall predictor variable fall between two membership functions. For instance, for the case of the predictor *rain2* of 458.5 mm in the *fuzzy_set_low* (Fig. 6b), the "medium" and "high" membership functions would be selected with the proportions of 0.198 and 0.802, respectively (calculated with Eqns. 12 and 13, respectively). Then, for each selected membership function, the number of flowers is picked from the best-fit density function and multiplied by the proportion. The number of flowers produced for a given plant is the sum of both values.

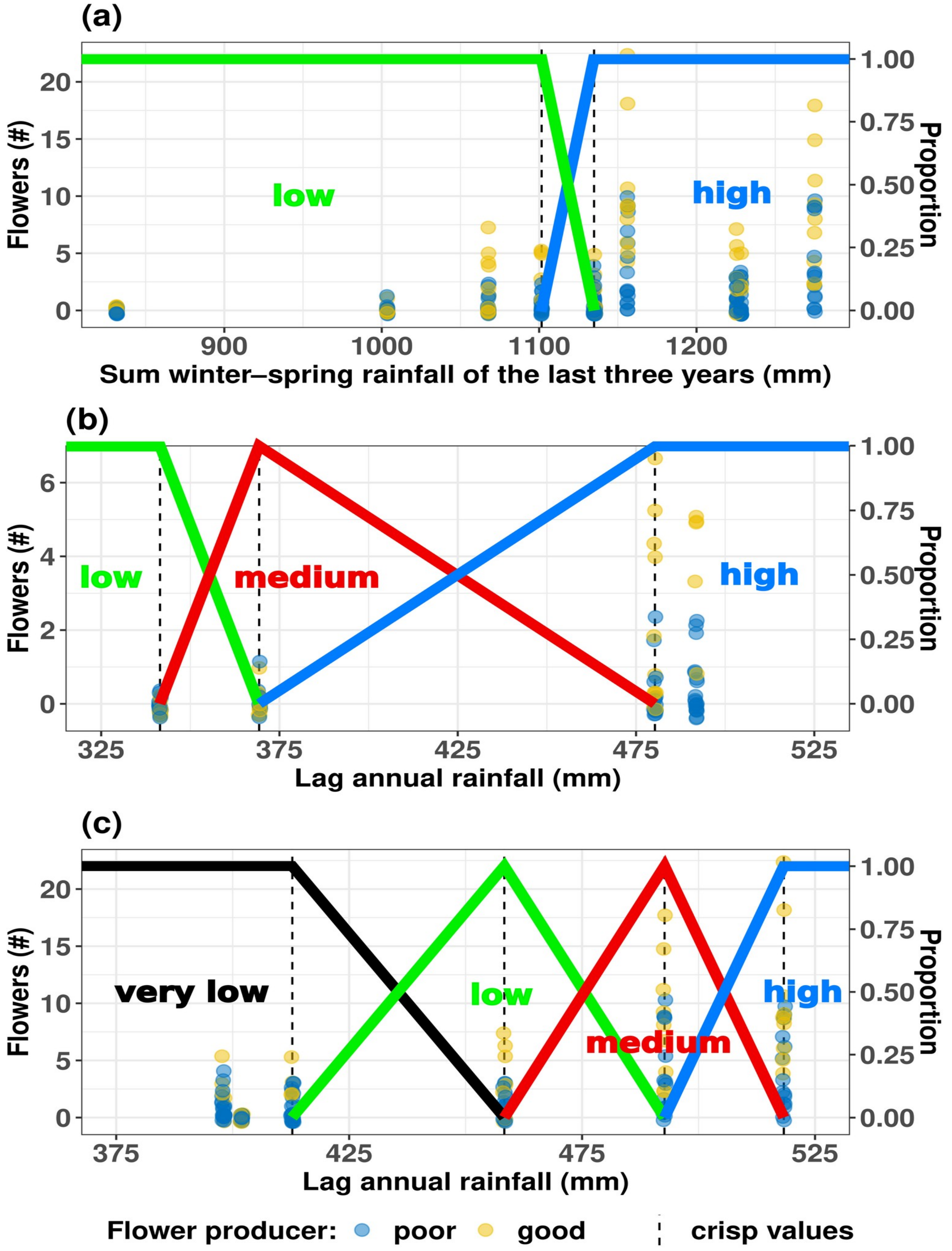

Fig. 6: *Hierarchical fuzzy sets used to classify plant performance. (a) Initial transitional fuzzy set using 'sum of winter–spring rainfall of the last three years', where the membership function labeled as 'low' directs to fuzzy_set_low (b), and the membership function labeled 'high' directs to fuzzy_set_high (c). These subsequent fuzzy sets (b and c) detail the classification of flower counts into 'poor' and 'good' producers, with each flower count within each membership function fitted to a density curve (see Table 7 and Fig. 7 for related density functions).*

Table 4: *Vertex values defining each membership function in Fuzzy_set_1 (see Fig. 6a)*

Name	Shape	left	left middle	right middle	right
low	trapezoid	700	800	1101.6	1134.9
high	trapezoid	1101.6	1134.9	1500	1600

Table 5: *Vertex values defining each membership function in Fuzzy_set_low (see Fig. 6b)*

Name	Shape	left	left middle	right middle	right
low	trapezoid	200	250	341.5	369.3
medium	triangle	341.5	369.3	369.3	480.3
high	trapezoid	369.3	480.3	700	800

Table 6: *Vertex values defining each membership function in Fuzzy_set_high (see Fig. 6c)*

Name	Shape	left	left middle	right middle	right
very low	trapezoid	200	250	412.8	458.3
low	triangle	412.8	458.3	458.3	492.7
medium	triangle	458.3	492.7	492.7	518.3
high	trapezoid	492.7	518.3	800	900

Table 7: *Flower count probability density functions for poor and good producers. The Fuzzy set "low" corresponds to Fig. 6b, and "high" to Fig. 6c.*

Fuzzy set	Membership function	Flower producer class	Distribution type	Distribution parameters
low	low	poor	none	zero flowers
		good	none	zero flowers
low	medium	poor	Poisson	lambda = 0.071
		good	Poisson	lambda = 0.111
low	high	poor	geometric	prob = 0.673
		good	geometric	prob = 0.288
high	very low	poor	geometric	prob = 0.618
		good	geometric	prob = 0.415
high	low	poor	Poisson	lambda = 1.214
		good	geometric	prob = 0.250
high	medium	poor	geometric	prob = 0.203
		good	negative binomial	size = 3.254, mu = 8.443
high	high	poor	geometric	prob = 0.205
		good	negative binomial	size = 6.275, mu = 10.100

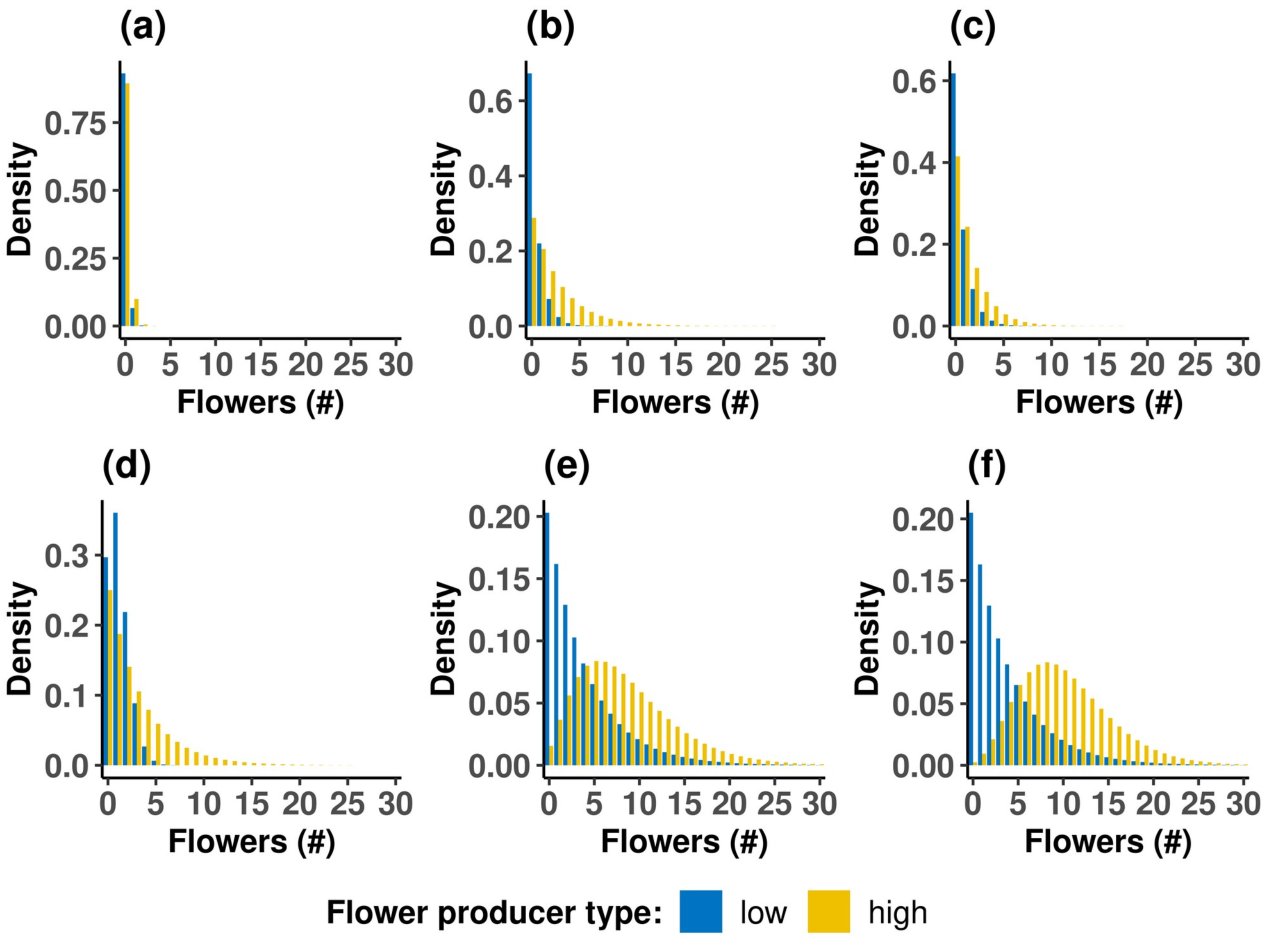

Fig. 7: *Flower count probability density functions for poor (blue) and good (yellow) producers. Each subplot corresponds to a membership function in Fig. 6: (a) "medium", and (b) "high" membership functions in fuzzy_set_low (Fig. 6b). (c) "very low", (d) "low", (e) "medium", and (f) "high" membership functions in fuzzy_set_high (Fig. 6c). The coefficient values for each density distribution are in Table 7.*

$$trianlge(x)\begin{cases}0, & x\leq a\vee x\geq c\\ \frac{x-a}{b-a}, & a>x<b\\ \frac{c-x}{c-b}, & b>x<c\\ 1, & x=b\end{cases} \tag{12}$$

where,

a, b and c correspond to the x-axis values of the vertices of the triangular membership function (from left to right), and x is the predictor's on the x-axis.

$$trapezoid(x)\begin{cases}0, & x\leq a\vee x\geq c\\ \frac{x-a}{b-a}, & a>x<b\\ \frac{d-x}{d-c}, & c>x<d\\ 1, & b\geq x\leq c\end{cases} \tag{13}$$

where,

a, b, c, d correspond to the x-axis values of the vertices of the trapezoidal membership function (from left to right), and x is the predictor's on the x-axis.

2.7.9 Viable seeds per cone age

The calculation of viable seeds per cone age was calculated at the beginning of the simulation because it did not change during the simulation. It was as follows: First, the potential number of seeds was calculated by multiplying the number of cones by the number of follicles per cone, by the number of seeds per follicle (Eqn. 14), followed by taking the proportion of seeds that developed embryos (firm seeds) (Eqn. 15). Then, the number of potential seeds was reduced by the age-dependent insect-damaged seeds and decayed seeds sub-submodels Enright et al. (1996) (Eqns. 16 and 17). We denominated here these two functions as accumulated insect-damaged seed equation, *AI(age)*, and accumulated decayed seeds, *AD(age)*, and are as follows. *AI(age)* is the fraction of lost seeds due to seed decay at a given seed age. The number of seeds with a given age would be *Nseeds (age) = N0 – N0 * AI(age)* considering only insect damage, where *N0* refers to the initial number of seeds:

$$Potential\, seeds = number\ of\ cones * follicles_{cone} * seeds_{follicle} \quad (14)$$

$$Firm\, seeds\, stored = potential\, seeds * firm \quad (15)$$

$$AI\left(age\right) = insect_a * age + insect_b \quad (16)$$

where,

$insect_a$ = 0.02 y^{-1}, and

$insect_b$ = 0.1

$$AD(age) = \exp(decay_a * age - decay_b) \quad (17)$$

where,

$decay_a$ = 0.34 y^{-1}, and

$decay_b$ = 5.95

Since the model runs on an annual resolution, calculating the seeds lost per cone age requires their subtraction from the accumulating seed store at each time step. That is, the non-accumulated seed loss by insect-damaged, *I(age)*, and decayed seeds, *D(age)*, are:

$$I(age) \begin{cases} AI(age), & age = 1 \\ AI(age) - AI(age-1), & age > 1 \end{cases} \quad (18)$$

$$D(age) \begin{cases} AD(age), & age = 1 \\ AD(age) - AD(age-1), & age > 1 \end{cases} \quad (19)$$

The proportion of seeds dispersed from cones due to spontaneous follicle rupture in the absence of fire was estimated for each cone age for *B. hookeriana* populations at Eneabba in 1986 (unpublished). We fitted a logistic growth curve to the accumulated proportion of open follicles per cone age, *AO(age)*, estimating the function parameters from weighted least-squares (see Section 3):

$$AO(age)=\frac{open_a}{1+\exp(open_c*(open_b-age))} \quad (20)$$

where,

$open_a$ = 0.3896,

$open_b$ = 6.6873 years, and

$open_c$ = 0.9588 $years^{-1}$

Thus, the aggregated number of lost seeds due to spontaneous follicle opening would be given by *Nloss = N0 – N0 * A0(age)*. As it is for the inter-fire seed loss equations, the annual proportion of open follicles per cone age, *O(age)*, is the subtraction between the target cone age and the previous cone age to the target:

$$O(age)\begin{cases} AO(age), & age=1 \\ AO(age)-AO(age-1), & age>1 \end{cases} \quad (21)$$

Thus, the number of lost seeds for one year due to spontaneous follicle opening would be given by *Nloss (age) = N0 * 0(age)*.

Finally, the proportion of viable seeds taken from the remaining seeds was applied (viability of seeds of 0.744; Enright et al., 1996). This calculated number of viable seeds per cone age (i.e., derived parameter) was used in the dispersal sub-model and the output of the seed dynamics model.

3. Data evaluation

This TRACE element provides supporting information on: The quality and sources of numerical and qualitative data used to parameterize the model, both directly and inversely via calibration, and of the observed patterns that were used to design the overall model structure. This critical evaluation will allow model users to assess the scope and the uncertainty of the data and knowledge on which the model is based.

Summary:

There are a total of 72 parameters in the MetaSqueeze model, all of which can be specified by the user. 45 relates to plant demography, 10 to dispersal, 11 to fire, three to population characteristics, and three to landscape characteristics. Two variables are not currently used but are retained to increase the model's flexibility and allow easy reparameterization for other applications. Two parameters were calibrated using a pattern-based modeling approach. The complete analysis of the data parameterization is included in the R script "calibration_and_analysis.qmd". Data analysis and visualization were performed using R v.3.5.3 (R Core Team 2019). We used the following R packages: 'ggplot2' v3.3.6 (Wickham, 2016), 'plotly' v4.10.0 (Sievert, 2020), 'dplyr' v1.0.9 (Wickham et al., 2022), 'fitdistrplus' v1.1.8 (Delignette-Muller & Dutang, 2015), 'plyr' v1.8.7 (Wickham, 2011), 'ggpbur' v0.4.0 (Kassambara, 2020), 'ggrepel' v0.9.1 (Slowikowski, 2021), 'reticulate' v1.26 (Ushey et al., 2022).

Section contents

3. Data evaluation.....45
3.1 The parameters related to study area.....46
3.2 The parameters and data related to metapopulation.....46
3.3 The parameters related to fire.....47

3.4 The parameters and data related to dispersal....48
3.4.1 Dispersal kernel of LDD of seeds by post-fire wind....49
3.4.2 Number of follicles....50
3.5 The parameters and data related to plant demographics and characteristics....52
3.5.1 Mortality rate curves....55
3.5.2 Open follicles per cone age....56
3.5.3 Habitat quality....57
3.5.4 Plant performance classes....59
3.5.5 Annual flower count data (i.e., flower production)....61

3.1 The parameters related to study area

The study area covered a 3 km × 5 km area of Eneabba Sandplain in South-West Australia (He et al., 2010), comprised of 100 m × 100 m grid cells (Table 8). We used a 100 m grid cell size resolution because the minimum distance between habitat patches (i.e., dunes) in Eneabba is 100 m (He et al., 2010). Furthermore, since cohorts and plants are spatially implicit within a population (i.e., habitat patches), the use of higher resolution (i.e., smaller grid cell size) would not necessarily increase the prediction power of the model significantly.

Table 8: *Parameters related to study area.*

Parameter	Value	Code name	Description [units] (reference)
cell_size	100	cell_size	Grid cell length of the study area [m].
study_size_x	3000	study_size_x	Study are length in the x-axis [m] (He et al., 2010).

study_size_x	5000	study_size_y	Study area length in the y-axis [m] (He et al., 2010).

3.2 The parameters and data related to metapopulation

The calibrated initial conditions of the sub-populations are shown in Table 9.

Table 9: *Initialization input file of the metapopulation. Eighteen habitat patches (i.e., population ID) were initially occupied, and the other 17 were unoccupied.*

Population ID	Size (ha)	Individuals	Habitat quality
1	21	5	low
2	14	254	low
3	47	826	low
4	49	917	low
5	4	34	low
6	54	292	low
7	8	151	low
8	10	2	low
9	22	62	low
10	5	66	low
11	7	88	low
12	5	72	low
13	3	14	low
14	14	171	low

Population ID	Size (ha)	Individuals	Habitat quality
15	16	93	low
16	16	196	low
17	8	65	low
18	18	255	low

3.3 The parameters related to fire

Table 10: *The parameters related to fire.*

Parameter	Value	Code name	Description [units] (reference)
fire_interval_scn	deterministic (0)	fire_interval_scn	Fire interval scenario [deterministic (0), truncated normal (1), Weibull (2)].
fire_size_scn	deterministic (0)	fire_size_scn	Fire size scenario [deterministic (0), truncated normal (1), Weibull (2)].
fire_scale	patchy (0)	fire_scale	Fire scale [patchy (0), entire study area (1)].
fire_size_x	2121.32	fire_size_x	Length of the semi x-axis of the fire ellipse [m].
fire_size_y	3535.53	fire_size_y	Length of the semi y-axis of the fire ellipse [m].
fire_interval_mean	17	fire_interval_mean	Fire interval mean [years].

Parameter	Value	Code name	Description [units] (reference)
fire_interval_lower_cut	1	fire_interval_lower_cut	Minimum fire interval [years].
burned_lower_cut	3	burned_lower_cut	Minimum fire interval needed to be burned, i.e., minimum fuel load [years] (Groeneveld et al., 2008).
burned_upper_cut	12	burned_upper_cut	Fire interval in which the probability to be burned is 100% [years] (Groeneveld et al., 2008).
fire_a	-	fire_a	Fire interval parameter for density distribution (e.g., Weibull distribution, shape parameter). Note: not used in this study.
fire_b	-	fire_b	Fire interval parameter for a density distribution (e.g., Weibull distribution, scale parameter). Note: not used in this study.

3.4 The parameters and data related to dispersal

Table 11: *The parameter related to dispersal.*

Parameter	Value	Code name	Description [units] (reference)
wind_prop	0.15	wind_prop	Proportion of LDD of seeds by post-fire wind.
wind_a	6.79	wind_a	Log mean parameter of the log-normal probability density function, i.e., dispersal kernel by post-fire wind
wind_b	0.68	wind_b	Log standard deviation parameter of the log-normal probability density function, i.e., dispersal kernel by post-fire wind
wind_direction	random (0)	wind_direction	Wind direction [random (0)] (He et al., 2010).
birds_dist_max	1250	birds_dist_max	Maximum distance of cone dispersal by cockatoos [m] (He

Parameter	Value	Code name	Description [units] (reference)
			et al., 2004).
birds_prop	0.07	birds_prop	Proportion of LDD of cones by cockatoos (Witkowski et al., 1994).
follicles_distr_type	nbinom_mu (3)	follicles_distr_type	Probability density function type of number of follicles per fertile cone.
follicles_distr_a	6.22	follicles_distr_a	"size" parameter of the negative binomial probability density function of number of follicles per fertile cone.
follicles_distr_b	10.08	follicles_distr_b	"mu" parameter of the negative binomial probability density function of number of follicles per fertile cone.
postfire_follicles_open	0.5	postfire_follicles_open	Proportion of follicles opened two hours after the fire (Enright & Lamont, 1989).

3.4.1 Dispersal kernel of LDD of seeds by post-fire wind

The dispersal kernel of LDD of seeds by post-fire wind was obtained by fitting the observed immigrants (He et al., 2010) to three of the most common density distributions used: Weibull,

gamma, and lognormal. The best fit (lowest AIC value, see Table 12) was a log-normal distribution with meanlog = 6.79 m and sdlog = 0.68 m, parameters wind_a and wind_b, respectively. Fig. 8 and Table 12 show the summary statistical results of fitting distributions.

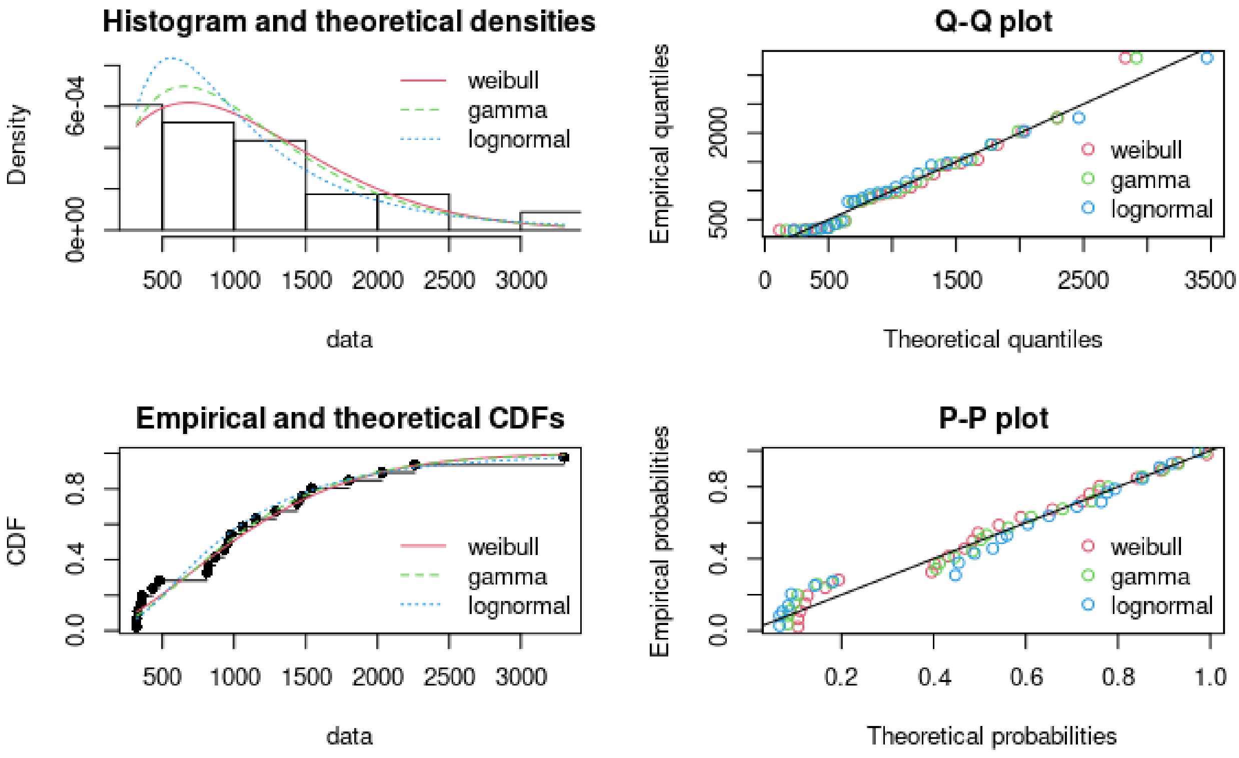

Fig. 8: *The results of the three probability density functions fitted to the observed immigration data (He et al., 2010).*

Table 12: *The AIC values of the three probability density functions fitted to the observed immigration data (see Fig. 8).*

Distribution type	AIC value

lognorm	363.44
gamma	363.80
Weibull	364.79

3.4.2 Number of follicles

The number of follicles per dispersed cone by cockatoos was drawn randomly from the fitted density curve for the baseline or current climate scenario (Fig. 9). The best fit for the baseline scenario was the negative binomial distribution (size = 6.22, and mu = 10.08), and for the current scenario was the Poisson distribution (lambda = 7.33).

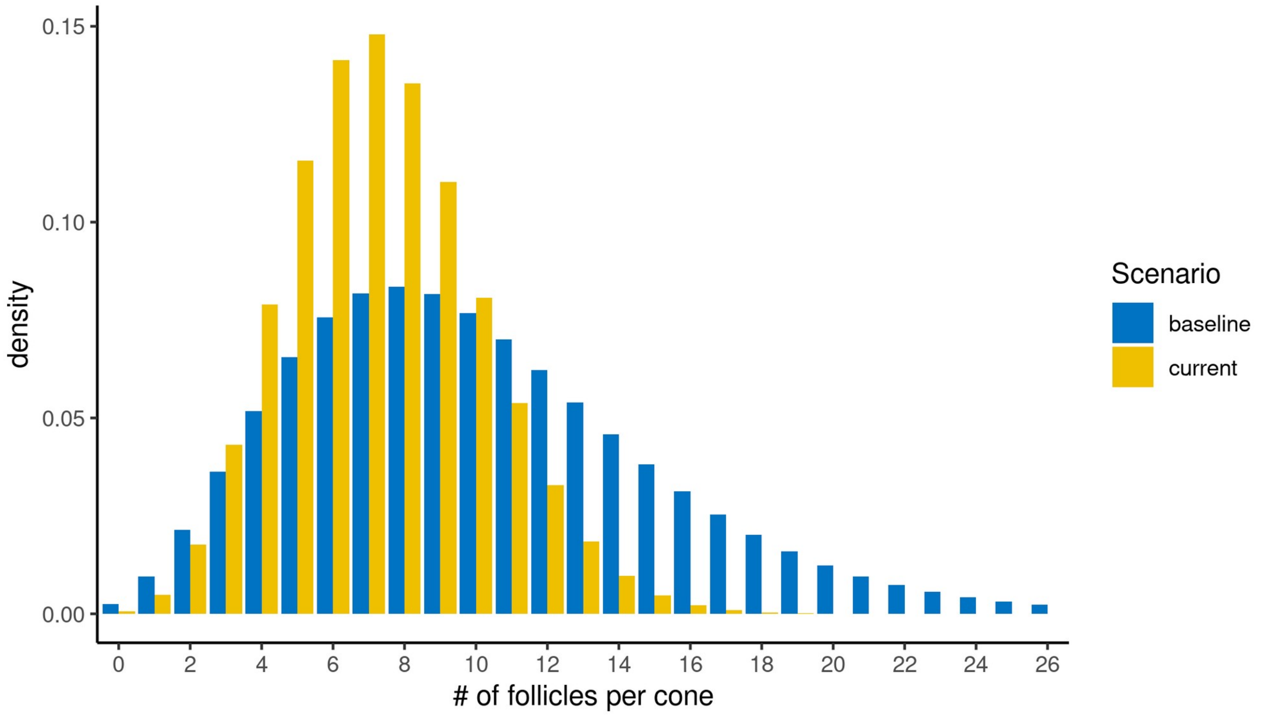

Fig. 9: *The fitted density curves of the number of follicles per cone for the baseline dataset (blue, negative binomial distribution with size = 6.22 and mu = 10.08) and for the current dataset (yellow, Poisson distribution with lambda = 7.33).*

On the other hand, we used the mean number of follicles in order to calculate the canopy seed bank, i.e., 9.97 follicles in 1986 (baseline scenario) and 7.32 in 2018 (current scenario) (Fig. 10).

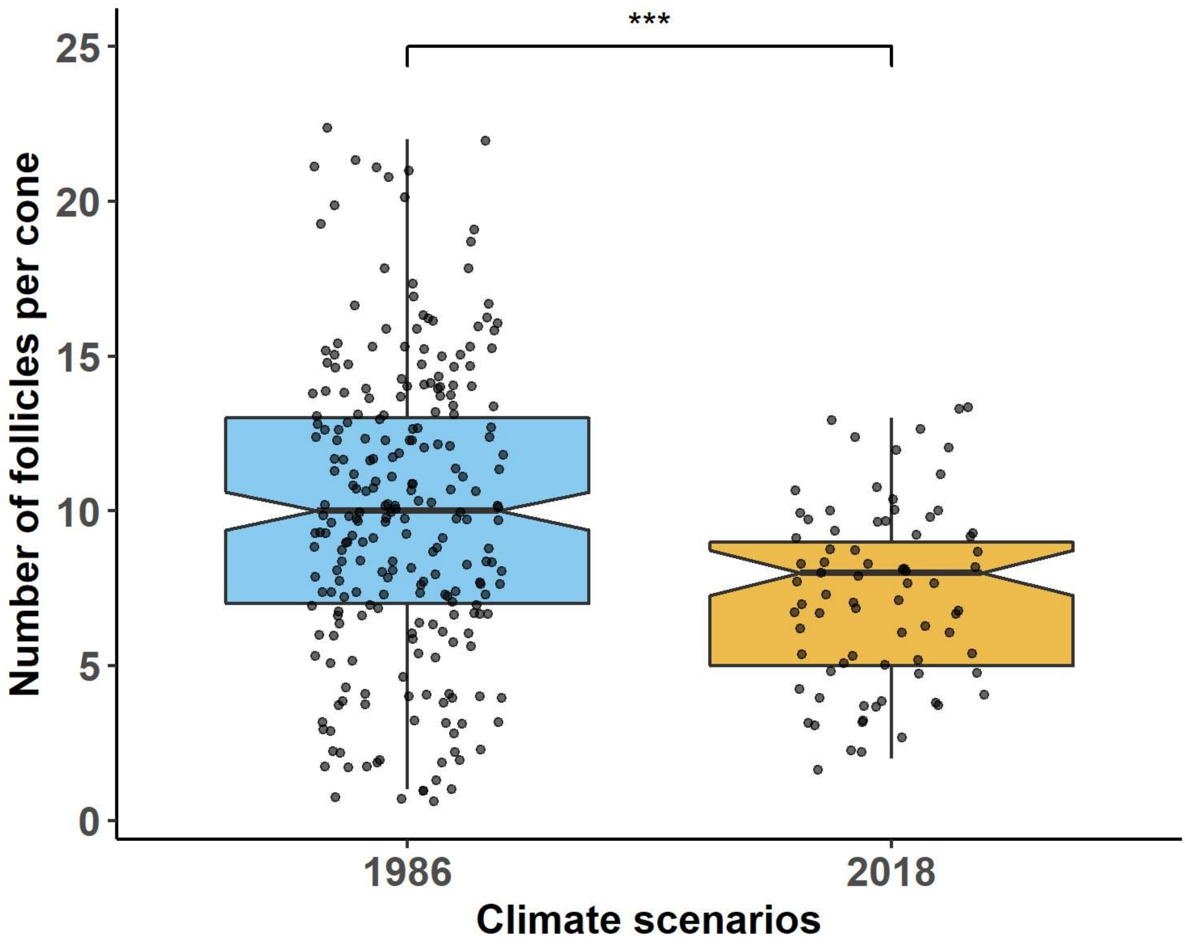

Fig. 10: *Number of follicles per fertile cone (i.e. cones with one or more follicles) in 1986 and 2018. Cones were burned to expose and rupture the follicles, and the number of follicles per cone was counted. In 2018 we collected cones aged 1, 3, and 5 years from 12 plants (n = 77 fertile cones) in a plot (HD8) near our current long-term monitoring plot (HD1). We compared these data with 1986 data for 1, 3, and 5-year-old cones from the same ten plants studied in the proportion of fertile cones (n = 243 fertile cones). A Mann-Whitney test showed that the number of follicles per cone was greater for the 1986 data (median = 10) than for the 2018 data (median = 8), W = 12545.5, p < 0.0001. As model parameters, we used the mean number of follicles per cone, i.e., 9.97 follicles in 1986 (i.e. baseline scenario) and 7.32 in 2018 (current scenario).*

3.5 The parameters and data related to plant demographics and characteristics

Most parameters related to plant demography were from literature and our previous non-spatial population model (Souto-Veiga et al., 2022). Table 13 summarizes all the plant demographic parameters.

Table 13: *The parameters related to plant demography.*

**Parameter**	**Value**	**Code name**	**Description [units] (reference)**
longevity	999	longevity	Maximum plant longevity [years].
young	5	young	Age of the plants in which they begin to produce fertile cones [years] (Enright et al., 1996).
adult	15	adult	Age of the plants in which they reach the maximum flower production and survival [years] (Enright et al., 1996, 1998).
mort_scn	mean absolute (1)	mort_scn	Five mortality scenario (see Section 2.74, Inter-fire plant mortality).
recruit_min	0.911	mort_recruit_post_min	Minimum mortality probability

Parameter	Value	Code name	Description [units] (reference)
			of recruitment (Enright & Lamont, 1989).
recruit_max	0.987	mort_recruit_post_max	Maximum mortality probability of recruitment (Groeneveld et al., 2002).
recruit_mean	0.923	mort_recruit_post_mean	Mean mortality probability of recruitment (Enright & Lamont, 1989; Groeneveld et al., 2002).
recruit_interfire	0	recruit_interfire	Inter-fire recruitment [%]. Note: We set to zero because we excluded inter-fire recruitment in this study. The theoretical inter-fire recruitment is 5% (Enright et al., 1998).
recruit_weather	0.06	recruit_weather	Proportion of weather impacts on recruitment. Approx. 6% per 100 mm around the long-term average winter–spring rainfall (Keith et al., 2014).
sen_age	25	senescence_age	Age of increased mortality [years] (Enright et al., 1998).
sen_incr	0.01	senescence_increase	Annual increase in mortality

Parameter	Value	Code name	Description [units] (reference)
			Probability due to senescence (Enright et al., 1998).
mort_min	0.02	mort_min	Minimum plant mortality probability (plant age ≥ 1 year).
mort_a	0.2877622	mort_a	Parameter 'a' of Eqn. 5 to calculate plant mortality (plant age ≥ 1 year) [years].
mort_b	0.0000000	mort_b	Parameter 'b' of Eqn. 5 to calculate plant mortality (plant age ≥ 1 year) [mm^{-1}].
mort_c	0.3179949	mort_c	Parameter 'c' of Eqn. 5 to calculate plant mortality (plant (plant age ≥ 1 year).
mort_d	0.6475991	mort_d	Parameter 'd' of Eqn. 6 to calculate plant mortality (plant age ≥ 1 year) [years].
mort_e	0.0000000	mort_e	Parameter 'e' of Eqn. 6 to calculate plant mortality (plant age ≥ 1 year) [mm^{-1}].
mort_f	0.2867799	mort_f	Parameter 'f' of Eqn. 6 to calculate plant mortality (plant age ≥ 1 year).

Parameter	Value	Code name	Description [units] (reference)
cone_cycle	1	cone_cycle	Time required for cones and seeds to reach maturity after successful pollination of the flowers [years].
seed_longevity	12	seed_longevity	Maximum seed longevity [years] (Enright et al., 1996).
flower_scn	lme_2 (1)	flower_scn	Flower equation. See submodel Flower production [lme_1 (0), lme_2 (1), nlme_2 (2)].
$flower_a$	9.179382	flower_age_a	Parameter 'a' of Eqn. 8 to calculate the mean flower production per plant based on plant age [#].
$flower_b$	8.710231	flower_age_b	Parameter 'b' of Eqn. 8 to calculate the mean flower production per plant based on plant age [years].
$flower_c$	0.620826	flower_age_c	Parameter 'c' of Eqn. 8 to calculate the mean flower production per plant based on plant age [$years^{-1}$].
flw_a	0.053176	flower_weather_a	Parameter 'a' of Eqn. 10 to

Parameter	Value	Code name	Description [units] (reference)
			calculate the mean flower production per plant based on rainfall conditions under baseline climate conditions [mm^{-1}] (Keith et al., 2014).
flw_b	–15.212370	flower_weather_b	Parameter 'b' of Eqn. 10 to calculate the mean flower production per plant based on rainfall conditions under baseline climate conditions (Keith et al., 2014).
flw_c	0.022191	flower_weather_c	Parameter 'c' of Eqn. 11 to calculate the mean flower production per plant based on rainfall conditions under current climate conditions [mm^{-1}].
flw_d	0.023055	flower_weather_d	Parameter 'd' of Eqn. 11 to calculate the mean flower production per plant based on rainfall conditions under current climate conditions [mm^{-1}].
flw_e	0.000000	flower_weather_e	Parameter 'e' of Eqn. 11 to

Parameter	Value	Code name	Description [units] (reference)
			calculate the mean flower production per plant based on rainfall conditions under current climate conditions.
fertile	90 (baseline), 65 (current)	fertile	Fertile cones per plant [%].
follicles	9.97 (baseline), 7.32 (current)	follicles	Follicles per fertile cone [#].
potential	2	seeds_potential	Potential number of seeds per follicle [#] (Enright et al., 1996).
firm	0.83	seeds_firm	Proportion of firm seeds (Enright et al., 1996).
viable	0.74	seeds_viable	Proportion of viable seeds (Enright et al., 1996).
insect_a	0.02	insect_a	Parameter 'a' of Eqn. 16 [years^{-1}] (Enright et al., 1996).
insect_b	0.18	insect_b	Parameter 'b' of Eqn. 16 (Enright et al., 1996).
decay_a	0.34	decay_a	Parameter 'a' of Eqn. 17 [years^{-1}]

Parameter	Value	Code name	Description [units] (reference)
			(Enright et al., 1996).
decay_b	–5.95	decay_b	Parameter 'b' of Eqn. 17 (Enright et al., 1996).
open_a	0.3896	open_a	Parameter 'a' of Eqn. 20 (Enright et al., 1996).
open_b	6.6873	open_b	Parameter 'b' of Eqn. 20 (Enright et al., 1996).
open_c	0.9588	open_c	Parameter 'c' of Eqn. 20 (Enright et al., 1996).
K	2500	carrying_capacity	Maximum number of adult plant individuals per ha [#] (Esther et al., 2008).
init_cones	85.87	init_cones	Initial cones per plant [#].
init_seeds	623.14	init_seeds	Initial mean viable seeds per plant [#].
long_term_rain	453.85	long_term_rain	Mean total winter–spring rainfall in 1965–1990 [mm] Eneabba Weather Station no. 8225; Australian Bureau of Meteorology.

3.5.1 Mortality rate curves

Fig. 11 shows the linear regression of the two mortality datasets: spring and autumn sites. These two curves were used in different mortality scenarios (see Section 2.7.4 Inter-fire plant mortality).

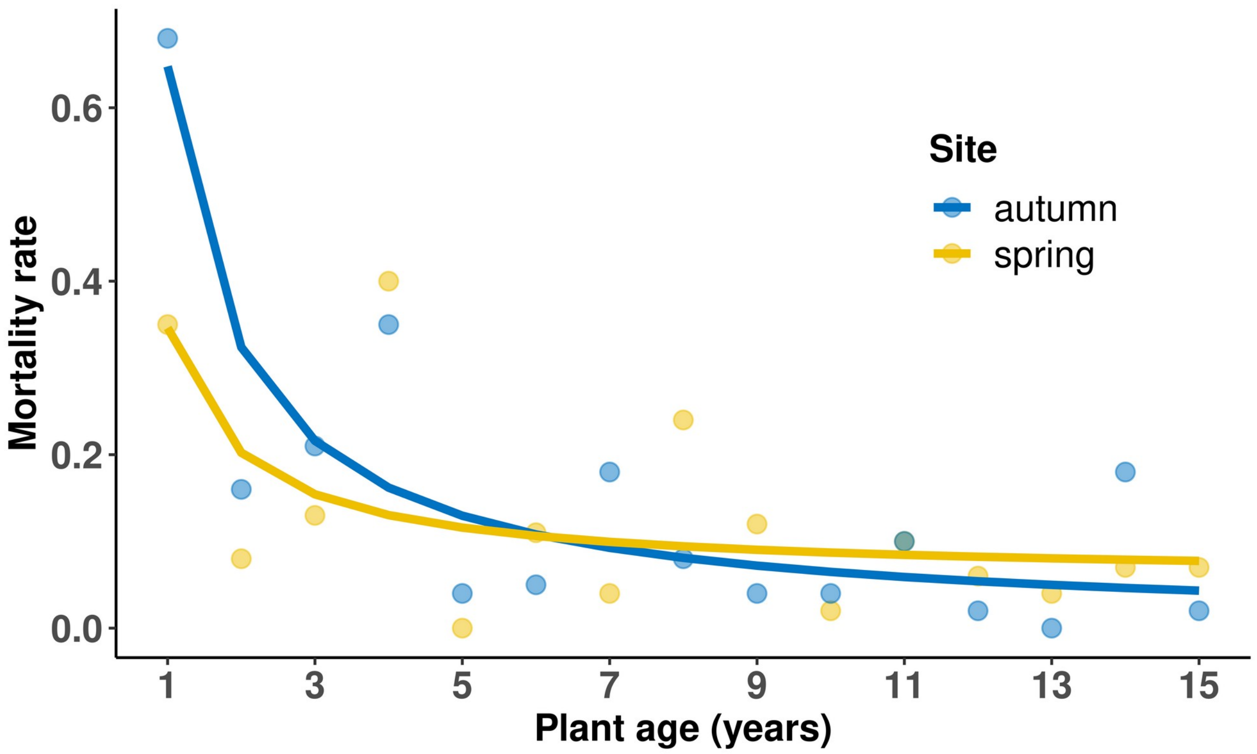

*Fig. 11: Linear regression analysis of the two mortality rate sites, i.e., spring site, and autumn site. The spring formula was as Mortality rate = mort_a / age + mort_b * lag rainfall + mort_c, where mort_a = 0.2877622, mort_b = –0.0005888, and mort_c = 0.3179949 with all the three coefficients at 0.05 level of significance, and the adjusted R-squared = 0.435. The autumn formula was as Mortality rate = mort_d / age + mort_e * lag rainfall + mort_f, where mort_d = 0.6475991, mort_e = –0.0006502, and mort_f = 0.2867799 with all the three coefficients at 0.01 level of significance, and the adjusted R-squared = 0.844.*

3.5.2 Open follicles per cone age

Seeds are dispersed during the inter-fire period from sporadic rupture of follicles. Using nonlinear (weighted) least-squares estimates, we fitted a logistic growth curve using unpublished data on the estimated proportion of follicles open per cone by cone age on 10 16-year-old *B. hookeriana* plants in 1986 in Eneabba (Fig. 12).

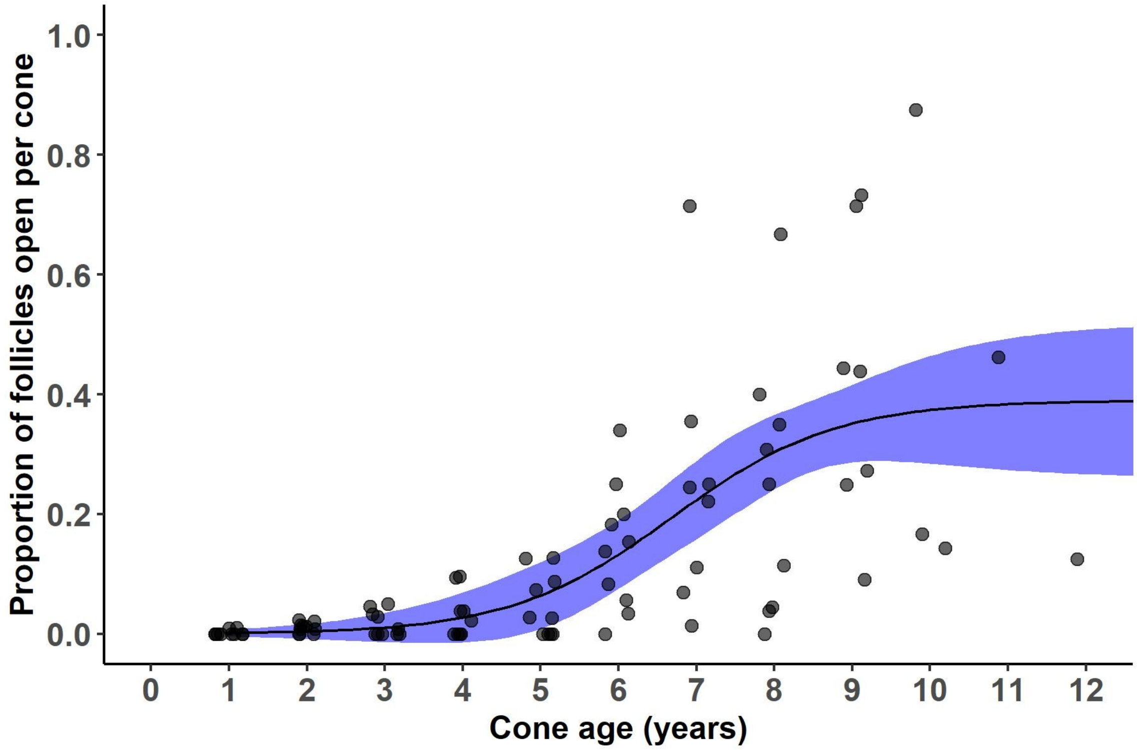

Fig. 12: *Relationship between cone age and the proportion of follicles open per cone in 1986 in Eneabba. The black curve represents the best fitted logistic growth model and the shaded area the 95% CI. The model is: OpenFollicles(cone_age) = a / (1 + exp(c (b – cone_age)), where a = 0.3896, b = 6.6873 years, and c = 0.9588 years–1, with 95% CIs [0.2903, 0.5690], [5.7092, 8.1585], and [0.5173, 2.5298], respectively, and S.E. = 0.1412, d.f. = 85.*

3.5.3 Habitat quality

We had flower count data from the same year (2016) in three different sites: Eneabba (our study area), South Eneabba, and Eneabba Reserve (Fig. 13). They showed a significant difference in flower production. Thus, considering the mean flower production in the Eneabba site (mean = 6.37 flowers) as "low" habitat quality with coefficient 1, we derived the habitat quality coefficient of mean increase in flower production by using a direct relationship. That is, for Eneabba Researve, with a mean of 8.76 flowers, the coefficient of mean increase was 1.38 ("moderate" habitat quality). For the South Eneabba site, with a mean of 14.2 flowers, the coefficient was 2.23 ("high" habitat quality) (Table 14). Each habitat patch (i.e., population) was assigned one of these three identified static habitat qualities (low, moderate, and high).

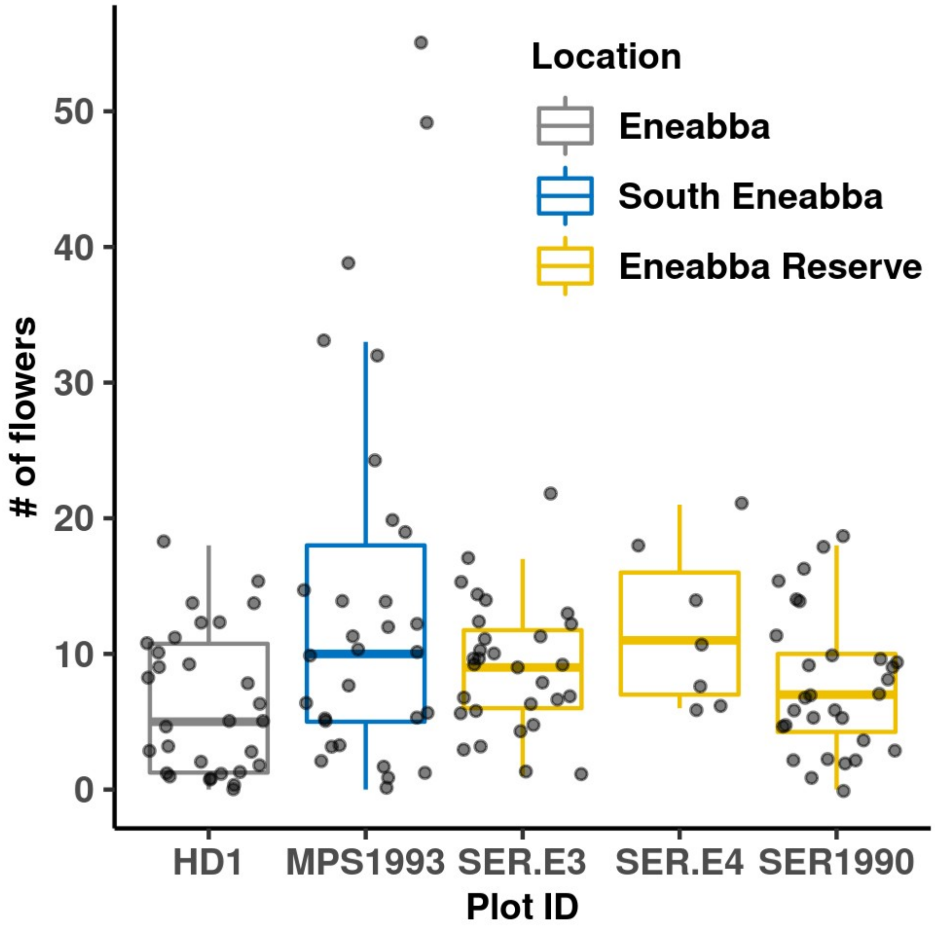

Fig. 13: *Flower count data from 2016 across Eneabba plots of varying post-fire ages, informing habitat quality categorization: 'low' (HD1, 18 years post-fire), 'moderate' (Eneabba Reserve: SER.E3, 29 years; SER.E4, 44 years; and SER1990, 26 years), and 'high' (South Eneabba: MPS1993, 23 years). Plant age is noted, although it does not significantly influence flower production beyond 15 years post-fire. The mean values served as a basis for calculating the habitat quality coefficients of mean increase in flower production, as outlined in Table 14.*

Table 14: *Coefficients of mean increase in flower production based on habitat quality, derived from 2016 flower count data. Eneabba (HD1, 18 years post-fire) serves as the 'low' quality reference (coefficient of 1.00). 'Moderate' quality from Eneabba Reserve plots (26–44 years post-fire) has a coefficient of 1.38, and 'high' quality at South Eneabba (23 years post-fire) has a coefficient of 2.23. Post-fire ages are provided, underscoring that flower production stabilizes after 15 years*

**Site (plot)**	**Habitat quality**	**Mean of flowers (μF)***	**Coefficient of mean increase in flower production**
Eneabba (HD1)	Low	6.367	1.00 (reference)
Eneabba Reserve (SER.E3, SER.E4, and SER1990)	Moderate	8.761	1.38 (μF of Moderate / μF of Low)
South Eneabba (MPS1993)	High	14.200	2.23 (μF of High / μF of Low)

*Refer to Fig. A5 for the distribution of flower counts.

3.5.4 Plant performance classes

The plant entity was included as a model expansion to include intraspecific variability in plant performance within dunes (i.e., individual-based approach). From our flower and cone count data under current climate conditions, we classified plants into two classes using the 75th percentile: poor producers (plants below the 75th percentile in accumulation of cones) and good producers

(above the 75th percentile). Data showed that good producers had significantly higher survival rates (Fig. 14). Table 15 shows the plant performance scenario used in the main manuscript.

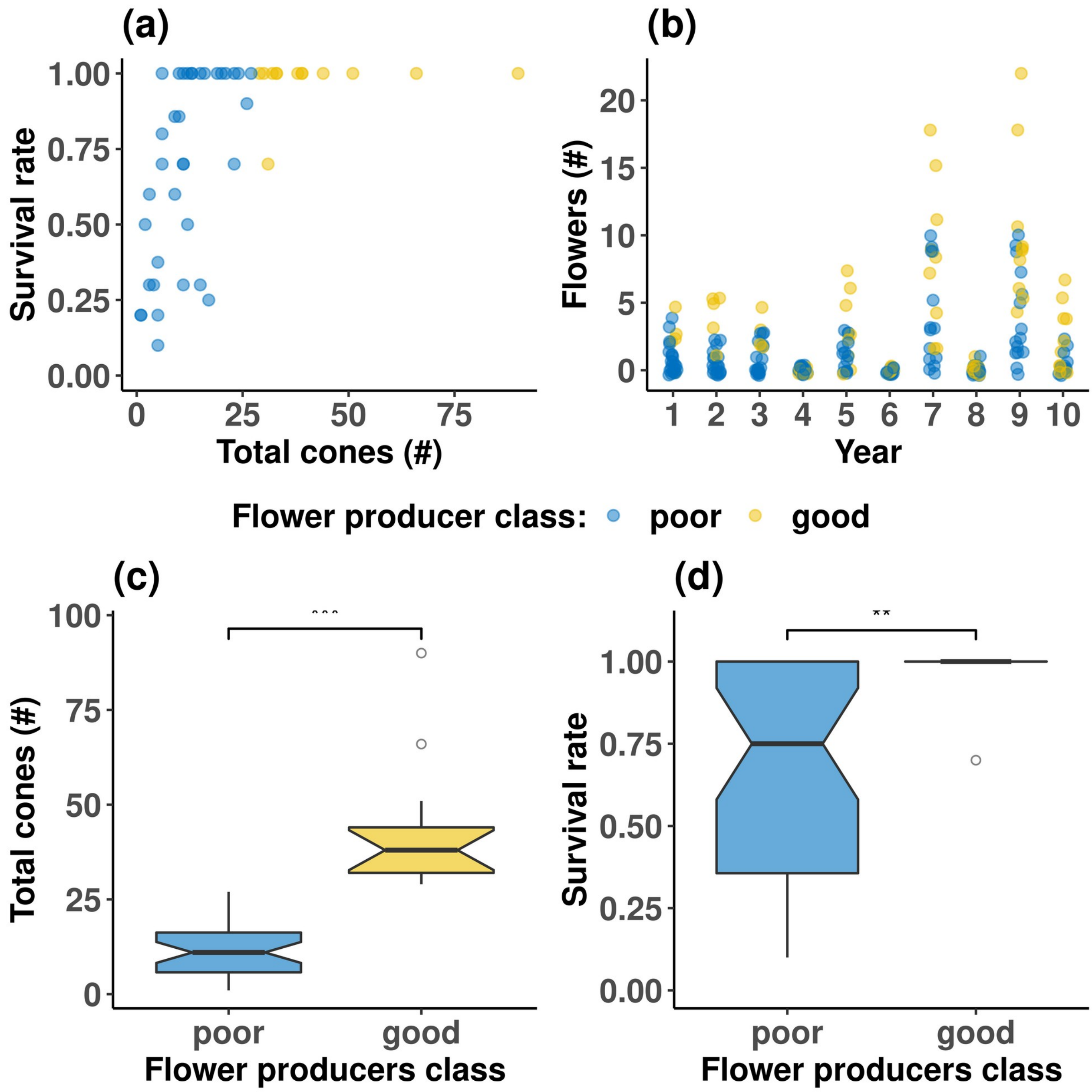

Fig. 14: *Classification of our flower and cone count data under current climate conditions: (a) plants were classified into two poor and good producers using the 75th percentile. (b) Flower count distribution per plant per year with the binary plant performance classification. (c) Good producers held a significantly (Mann-Whitney test) higher number of cones, and (d) had higher survival rates than poor producers.*

Table 15: *Classification of plant performance used in the main results in the main paper.*

Performance class	Performance probability	Performance mortality
poor (0)	0.75	0
good (1)	0.25	–0.06

3.5.5 Annual flower count data (i.e., flower production)

We have two datasets from even-aged cohort monitoring campaigns, in which the number of flowers has been counted once a year in mid-Spring. The baseline dataset ranges from 1988 to 2002 (plant ages 17 to 31 years), and the current dataset from 2008 to 2017 (plant ages 11 to 20 years), which is still ongoing. The current campaign started with 30 individuals in the first year (i.e., 2008). All plants were tagged with an identification number (tag). Due to a high mortality rate in the fourth year, it was decided to include new plants in order to have almost ~30 plants per year in the survey. We selected the closest living plant to the dead plant. In the current dataset, three plants (i.e., tags 604, 606, and 608) were not considered for the regression analysis since they have been receiving extra rainfall from another study. Furthermore, the ongoing flower count in the current campaign in 2018−2020 was not included in our study because the Eneabba weather station stopped working in March 2017.

Keith et al. (2014) found a strong relationship between mean annual inflorescence production and the total rainfall in winter–spring of the previous year with the baseline data. For the current data, the most correlated predictor variables were total annual rainfall of the previous year, and the sum winter–spring rainfall of the last three years. We fitted the regression lines for the current dataset

with linear mixed-effect models using the lme4 package version 1.1.26 (Bates et al. 2015). The variables “year” and “tag” were used as random intercepts.

In the current campaign flower count data there are too many zeros, so we used two different regression techniques for the current climate impacts of flower production: i) the linear mixed-effects model assuming that the residual errors have a Gaussian distribution, and ii) the generalised linear mixed-effects model for the negative binomial family. In addition, we also tested the simulations experiments excluding the second explanatory variable (i.e. the sum of winter–spring rainfall of the last three years) for both linear and generalised linear mixed-effects models under current climate. Thus, we tested in total four regression models for the flower production under current climate conditions in our previous study (Souto-Veiga et al., 2022) (Fig. 15). Table 16 shows the statistical results of the four regression models used in our simulations of our previous study..

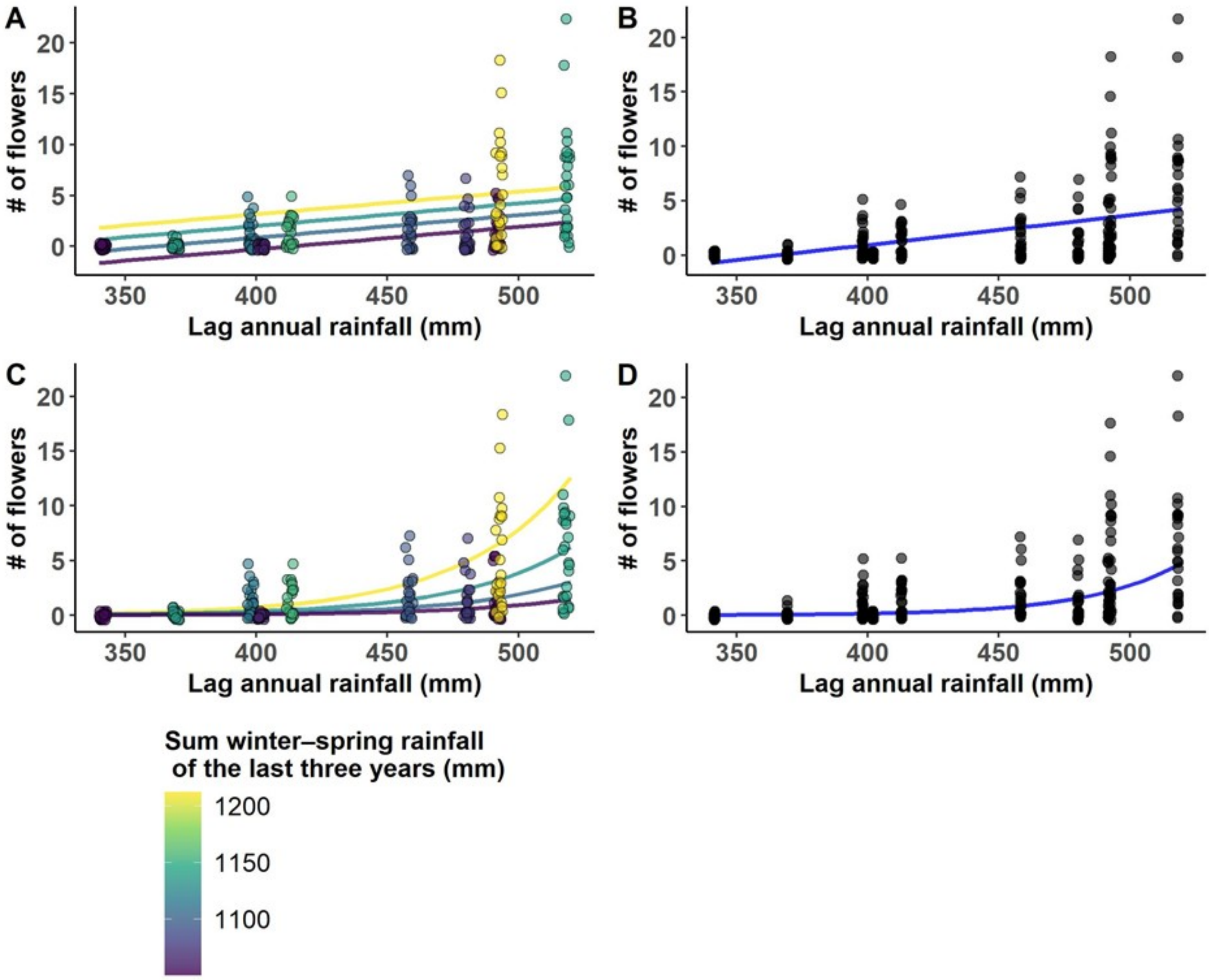

Fig. 15: *Dependence of flower production under current climate conditions (2008–2017, plant age 11–20 years) on the total annual rainfall of previous year (i.e., lag annual rainfall), and sum of winter–spring rainfall of the previous three years using (A) the linear mixed-effect model assuming that the residual error have a Gaussian distribution, and (C) the generalized linear mixed-effect model for the negative binomial family. Model versions B and D use only the predictor variable lag annual rainfall with the same modeling techniques of A and C, respectively. The four formulas had as random intercepts "year" and "tag". See statistical results in Table 16.*

Table 16: *Results of significant of the four linear-mixed models of flower production under current climate conditions (see Fig. 15).*

**Model**	**Predictor variable**	**Estimate of coefficients**		**Model significance**		
		**Estimate**	**95% CI**	**df**	**Deviance**	**Pr(>Chi)**
A	(intercept)	–33.345	[–48.176, –18.518]	1	1137.2	0.004
	lag annual rainfall	0.022	[0.010, 0.034]			
	sum winter–spring rainfall of the last three years	0.023	[0.009, 0.037]			
B	(intercept)	–10.043	[–17.853, –2.242]	2	1145.5	< 0.001
	lag annual rainfall	0.027	[0.001, 0.045]			
C	(intercept)	–26.765	[–45.777, 13.676]	1	692.69	0.032
	lag annual rainfall	0.024	[0.013, 0.039]			
	sum winter–spring rainfall of the last three years	0.014	[0.002, 0.029]			
D	(intercept)	–13.102	[–22.191, –6.673]	1	697.30	0.001
	lag annual rainfall	0.028	[0.014, 0.048]			

4. Conceptual model evaluation

This TRACE element provides supporting information on: The simplifying assumptions underlying a model's design, both with regard to empirical knowledge and general, basic principles. This critical evaluation allows model users to understand that model design was not ad hoc but based on carefully scrutinized considerations.

Summary:

The MetaSqueeze model expands a previous non-spatial population model (Souto-Veiga et al., 2022). The conceptual model is represented in Fig. 1 in Section 2. We discuss here the simplifying assumptions of the different submodels.

Section contents

4. Conceptual model evaluation....64
4.1 Plant mortality....64
4.2 Seed production and storage....65
4.3 Fire....65
4.4 Dispersal....66

4.1 Plant mortality

Banksia hookeriana is a fire-killed shrub; thus, no plants survive after a fire (Enright et al., 1996, 1998). Although there may be survival plants in prescribed fires due to their low intensity, we assume that all fires in the model are wildfires, which are typically highly intense crown fires (Hantson et al., 2017; Nolan et al., 2020; Silva et al., 2018). The probability of plant mortality

between fires depends on plant age and weather conditions. All the seeds are lost when a plant dies between fires (Enright et al., 1998). The different habitat quality of patches (i.e., populations) in our simulations only affect plants' flower production (see Section 3). In higher habitat quality, the survival rates are likely higher. However, we had no empirical data. The only explicit intraspecific competition between plants occurred in the submodel "density regulation of adult plants". Due to the lack of data, we could not incorporate other aspects, such as the competition-density effect or self-thining.

4.2 Seed production and storage

The seed dynamics (i.e., seed storage and loss) were disentangled into separated individual processes linked to empirical data, producing more quantitative and realistic predictions. The processes were: flower production, fertilization, number of follicles per cone, the proportion of firm seeds per cone, the proportion of viable seeds, and seed loss. The flower production was the only process that changed during the simulation run (i.e., dynamic process) because it was the only one where we had enough data to analyze correlation with explanatory variables. Plant age (Enright et al., 1996) and antecedent rainfall conditions (Keith et al., 2014; Souto-Veiga et al., 2022) were strongly correlated with flower production.

The rest of the processes were just taken from the average or linear regression based on cone age since we did not have enough data to correlate them with other explanatory variables (e.g., rainfall). For this reason, since only flower production was the only dynamic process in which plant age and weather conditions impacted the number of flowers produced, we calculated the number of viable seeds per cone at the beginning of the simulation.

4.3 Fire

We did not include the historical fire records in the region because they were much larger than our study area (3 km × 5 km), and thus, it is difficult to link these two different spatial scales. Therefore,

we chose the ellipse shape to create patchy fire events as it is the most used fire shape in ideal conditions (Glasa & Halada, 2008; Green, 1983), and most fires in the region are wind-driven with elliptical shapes (Enright et al., 2012). We defined the "baseline" patchy fire size as the circumscribed ellipse (Fig. 3, Section 2). With this approach, we could systematically test different fire sizes relative to the baseline size of the study area.

To determine if a population had sufficient fuel load to be burned, we used the fire spread formula of Groeneveld et al. (2008). This formula takes only the variable time since the last fire to determine the probability of a population being burned. We did not include a more complex fire modeling approach because it would make more complex to understand the results of the simulations and thus to answer the research questions set out in our study. Moreover, fire independence from other factors, such as weather conditions, allowed us to explore a broader range of scenarios.

4.4 Dispersal

We used a more probabilistic approach for dispersal processes. We would need more empirical data to use a more mechanistic approach for seed dispersal. Furthermore, only the habitat patches are located explicitly on the landscape matrix. Cohorts and plants are not spatially-explicit. We believe that using our dispersal kernel is a good approximation to include recolonizations and immigration-caused population growth effects events to simulate a spatially structured metapopulation.

Little is known concerning the dispersal of cones by cockatoos (He et al., 2004). Since cockatoos feed mainly from Banksia species (Johnston et al., 2016), we considered that they disperse cones only within the source patch or between patches, i.e., direct dispersal.

5. Implementation verification

This TRACE element provides supporting information on: (1) whether the computer code for implementing the model has been thoroughly tested for programming errors and (2) whether the implemented model performs as indicated by the model description.

Summary:

> **The computer code was continuously checked and tested during the implementation. Such tests were: code debugging observing particular state variables, implementation of processes individually before adding to the model, stress tests with extreme parameter values, and controlled simulation experiments. We followed the C++ core guidelines, used C++ code analysis (CppCoreCheck and Clang-tidy), and used gcc compiler flags for compilation to enable warnings.**

We rigorously evaluated the model to ensure that each submodel and the model behaved as intended. We first re-implemented our published non-spatial population model in C++ 17 (the old version was in C++ 11). After the re-implementation, we debugged all the submodels and created some simulation tests to see that the results were similar to the old version code: such as seed dynamics per plant.

We followed the C++ core guidelines to follow "modern C++" standards. Input parameters are read from text files. The main input simulation file is composed of subfiles. Each subfile corresponds to a group of related parameters (e.g., fire parameters, dispersal parameters). Before starting the simulation, all parameters are verified that they are in the correct format and range.

6. Model output verification

This TRACE element provides supporting information on: (1) how well model output matches observations and (2) how much calibration and effects of environmental drivers were involved in obtaining good fits of model output and data.

Summary:

> **In this study, the initial individuals per population and the percentage of long-distance dispersal (LDD) of cones and seeds were inversely determined via calibration. The rest of the parameters were all directly calculated from the literature data source and own data (see Section 3).**

We used the cross-multiplication method to calculate how many initial individuals per population with 623.14 viable seeds in the canopy per individual (mean viable seeds of 20–30-years-old plants in 1000 simulation runs) were needed at the beginning of the simulation to have a similar number of 8-year-old plants as observed in the field. This method was made for each mortality scenario tested. Table 17 shows the calculated mean initial individuals per population per mortality scenario in 1000 simulation runs.

The percentage of cones and seeds dispersed by cockatoos and post-fire wind were inversely calibrated (Railsback & Grimm, 2019) using two empirical patterns: the total immigration rate in the metapopulation and the average of genetic populations (i.e., population ID) per habitat patch. We calibrated both parameters for the four mortality scenarios tested (Fig. 16). In order to get similar levels of the observed immigration rate in He et al. (2010) with 5.5% or in He et al. (2004) with 6.8% (and more than two population IDs per habitat patch), LDD plants or immigrants plants had to have lower mortality than SDD plants in order to show the realistic percentage of cones and seeds dispersed (Figs. 16c and 16d). We selected 7% of cones dispersed by birds as it was observed

that approx. 7% of the flowers were removed by cockatoos (Witkowski et al., 1994). Concurrently, 15% of dispersed seeds by the post-fire wind were picked to reach the observed immigration rate with ±10% error (Fig. 16).

Table 17: *Calibration of initial plants per population in each mortality scenario.*

**Population ID**	**Pre-fire population size (# of individuals) in each mortality scenario**		
	**Mortality 0**	**Mortality 1**	**Mortality 2 and 3**
1	4	5	8
2	201	254	394
3	651	826	1276
4	723	917	1416
5	27	34	53
6	230	292	451
7	119	151	233
8	2	2	4
9	49	62	96
10	52	66	103
11	69	88	136
12	57	72	112
13	11	14	22
14	135	171	263
15	73	93	143
16	155	196	304
17	51	65	101

18	202	255	396

Note: the mortality scenarios 3 and 4 have the same number of initial plants (i.e., prefire population size), because there is no LDD, and thus no immigrants.

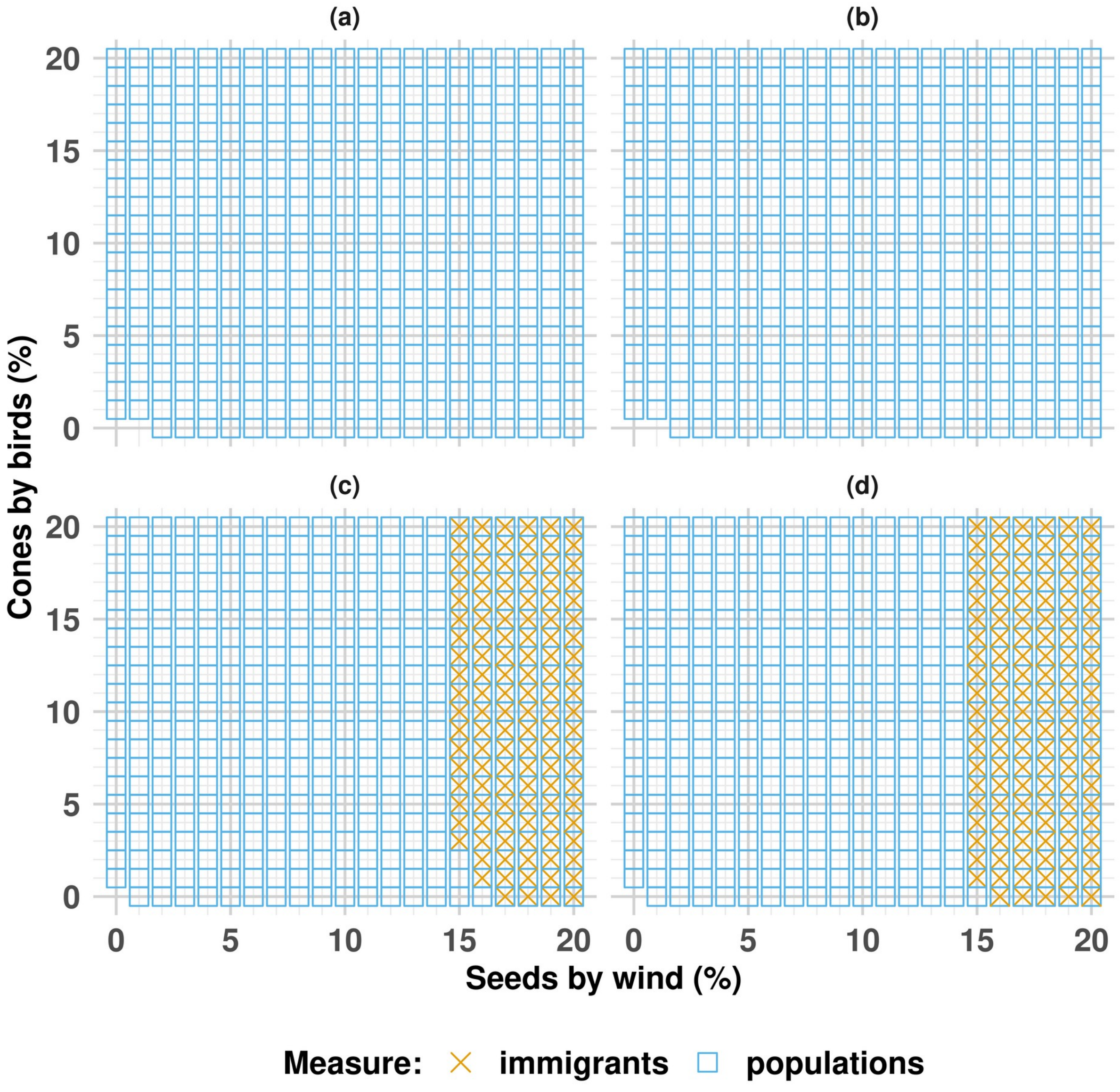

Fig. 16: Calibration results for LDD parameters via inverse modeling, showcasing the outcomes across the first four inter-fire plant mortality scenarios , which correspond to (a) age-weather relative impacts, (b) mean age-weather absolute impacts, (c) LDD cohorts with lower mortality than SDD cohorts, and (d) immigrant cohorts with lower mortality than resident cohorts, respectively. Each subplot examines a range of combinations between percentages of LDD of cones by birds (0–20%, y-axis) and LDD of seeds by wind (0–20%, x-axis), assessing their effectiveness in matching two empirical patterns: the percentage of immigrants and the number of population IDs per habitat patch. The model was considered successful in replicating these patterns when the immigrant percentage aligned with the observed immigration rate within a ±10% error (i.e., 4.95–7.48%) and when the number of population IDs was equal to or greater than two, as observed by He et al. (2004, 2010). The fifth mortality scenario is not shown, as its results mirrored those of scenarios (a) and (b), failing to differentiate mortality rates among cohort types and thus not providing additional insights into the calibration process aimed at replicating observed patterns of seed dispersal

7. Model analysis

This TRACE element provides supporting information on: (1) how sensitive model output is to changes in model parameters (sensitivity analysis), and (2) how well the emergence of model output has been understood.

Summary:

A local sensitivity analysis was performed altering one parameter at a time by ±5% and ±10%. We calculated the mean metapopulation persistence as a result of the model from 900 replicates (30 study area replicates and each study area replicate was 30 times replicated), where the maximum persistence time was set to 500 years. We measured the sensitivity of each variation by calculating the percentage deviation between the mean persistence time of the reference values and the mean persistence time for each varied parameter.

We conducted a local sensitivity analysis to identify which parameters are susceptible to changes. The sensitivity experiment input file was generated using the R script named *sensitivity_generator.R*, and the calculation of sensitivity measures in *calibration_and_analysis.qmd*.

Table 18 shows the parameter values used in the local sensitivity analysis. Parameter values that cannot exceed specific values, such as probabilities, were truncated when necessary. In addition, all parameters set as a natural number (i.e., a non-negative integer) were varied by taking the integer part of the altered value when the reference value was decreased and taking the next integer when it was increased. With this approach, all parameter variations were different from the reference value. For example, the reference value for seed longevity is 12 years, so the altered values are 10.8, 11.4,

12.6 and 13.2, and since seed longevity is a non-negative integer, the altered values selected were 10, 11, 13 and 14, respectively.

Most of the model parameters were robust to ±5% and ±10% shifts from their reference values, i.e., this variation of the parameters showed little effect on metapopulation persistence (Table 19). For example, all alterations of LDD parameters did not affect metapopulation persistence by more than 1%. The most sensitive parameters were those related to post-fire recruitment and flower production, where the persistence time changed by two and three times from the alteration of these parameters. Post-fire recruitment is more sensitive to flower production parameters. A 10% decrease in the upper limit and mean of post-fire recruitment mortality parameters increased metapopulation persistence by approx. 60%.

Table 18: *Parameter values used in the local sensitivity analysis, conducted under current climate conditions with deterministic fire intervals. This table presents the reference values for each parameter alongside their variations at –10%, –5%, +5%, and +10%. Parameters that were truncated due to their nature (e.g., probabilities) are indicated with their percentage of variation in parentheses*

**Parameter (code)**	**Reference**	**Variation**			
		**–10%**	**–5%**	**5%**	**10%**
carrying_capacity	2 500	2 250	2 375	2 625	2 750
init_cones	85.868	77.281	81.575	90.161	94.455
init_seeds	623.141	560.827	591.984	654.298	685.455
long_term_rain	453.848	408.463	431.156	476.54	499.233
fire_size_x	2 121.320	1 909.188	2 015.254	2 227.386	2 333.452
fire_size_y	3 535.534	3 181.981	3 358.757	3 712.311	3 889.087

Parameter (code)	Reference	Variation			
		–10%	–5%	5%	10%
fire_interval_mean	17	15	16	18	19
burned_lower_cut	3	2 (–33.3%)	2 (–33.3%)	4 (33.3%)	4 (33.3%)
burned_upper_cut	12	10 (–16.7%)	11 (–8.3%)	13 (8.3%)	14 (16.7%)
wind_prop	0.15	0.135	0.142	0.158	0.165
wind_a	6.787	6.108	6.448	7.126	7.466
wind_b	0.676	0.608	0.642	0.71	0.744
birds_dist_max	1 250.000	1 125.000	1 187.500	1 312.500	1 375.000
birds_prop	0.07	0.063	0.066	0.074	0.077
follicles_distr_a	7.325	6.592	6.959	7.691	8.057
follicles_distr_b	0	0	0	0	0
postfire_follicles_open	0.5	0.45	0.475	0.525	0.55
young	5	4 (–20%)	4 (–20%)	6 (20%)	6 (20%)
adult	15	13 (–13.3%)	14 (–6.7%)	16 (6.7%)	17 (13.3%)
recruit_post_min	0.911	0.82	0.865	0.957	0.987
recruit_post_max	0.987	0.911 (–7.7%)	0.938	1 (1.3%)	1 (1.3%)
recruit_post_mean	0.923	0.831	0.877	0.969	1
recruit_weather	0.06	0.054	0.057	0.063	0.066
senescence_age	25	22 (–12%)	23 (–8%)	27 (8%)	28 (12%)
senescence_increase	0.01	0.009	0.01	0.01	0.011
mort_min	0.02	0.018	0.019	0.021	0.022
mort_a	0.288	0.259	0.273	0.302	0.317

**Parameter (code)**	**Reference**	**Variation**			
		**–10%**	**–5%**	**5%**	**10%**
mort_b	−0.001	−0.001	−0.001	−0.001	−0.001
mort_c	0.318	0.286	0.302	0.334	0.35
mort_d	0.648	0.583	0.615	0.68	0.712
mort_e	−0.001	−0.001	−0.001	−0.001	−0.001
mort_f	0.287	0.258	0.272	0.301	0.315
cone_cycle	1	0 (–100%)	0 (–100%)	2 (100%)	2 (100%)
seed_longevity	12	10 (–16.7%)	11 (–8.3%)	13 (8.3%)	14 (16.7%)
flower_age_a	9.179	8.261	8.72	9.638	10.097
flower_age_b	8.71	7.839	8.275	9.146	9.581
flower_age_c	0.621	0.559	0.59	0.652	0.683
flower_weather_a	0.053	0.048	0.051	0.056	0.058
flower_weather_b	−15.212	−13.691	−14.452	−15.973	−16.734
flower_weather_c	0.022	0.02	0.021	0.023	0.024
flower_weather_d	0.023	0.021	0.022	0.024	0.025
flower_weather_e	−33.345	−30.010	−31.677	−35.012	−36.679
pollination	65	58.5	61.75	68.25	71.5
follicles	7.32	6.588	6.954	7.686	8.052
seeds	2	1.8	1.9	2.1	2.2
firm_seeds	0.83	0.747	0.788	0.872	0.913
viable_seeds	0.744	0.67	0.707	0.781	0.818

Parameter (code)	Reference	Variation			
		–10%	–5%	5%	10%
insect_a	0.02	0.018	0.019	0.021	0.022
insect_b	0.18	0.162	0.171	0.189	0.198
decay_a	0.34	0.306	0.323	0.357	0.374
decay_b	−5.950	−5.355	−5.652	−6.247	−6.545
open_a	0.39	0.351	0.37	0.409	0.429
open_b	6.687	6.019	6.353	7.022	7.356
open_c	0.959	0.863	0.911	1.007	1.055

Table 19: *Results of the local sensitivity analysisperformed under current climate conditions with deterministic fire intervals. It shows the percent deviation of the simulation results for the mean persistence time, relative to variations in each parameter (-10%, -5%, +5%, +10%). When a parameter is changed, all other parameters remain constant, allowing for a focused assessment of each parameter's influence. For the reference values and their specific variations, see Table 18. Results showing more than twice the percentage deviation in parameter change are highlighted.*

Parameter	Variation			
	–10%	–5%	5%	10%
carrying_capacity	–0.72	–0.12	–0.77	–1.4
init_cones	–0.29	–1.15	–0.34	–1.46

**Parameter**	**Variation**			
	**–10%**	**–5%**	**5%**	**10%**
init_seeds	–1.89	–0.24	0.05	–0.53
long_term_rain	37.86	14.42	–12.35	–20.54
fire_size_x	0.24	–0.72	–0.57	–1.26
fire_size_y	–1.24	–0.7	–0.15	–0.35
fire_interval_mean	–7.92	–3.42	1.6	3.81
burned_lower_cut	–0.3	–0.47	–1.02	–0.96
burned_upper_cut	–0.84	–1.13	–0.59	0.24
wind_prop	–1.09	–1.16	–1.15	–1.19
wind_a	0.19	–0.52	–0.56	–1.04
wind_b	–1.1	–0.97	–0.46	–0.35
birds_dist_max	–0.9	–0.07	–0.62	–0.72
birds_prop	–0.41	–0.54	–0.54	0.14
follicles_distr_a	–1	–1.79	–0.88	–1.44
follicles_distr_b	–1.55	–0.29	–1.59	–0.9
postfire_follicles_open	–0.15	–0.71	–0.33	–1.12
young	–0.8	–0.99	–0.67	–0.86
adult	–2.37	–1.19	–0.27	–0.15
recruit_post_min	–1.25	0.12	–4.34	–24.29
recruit_post_max	62.1	34.59	–47.06	–5.74
recruit_post_mean	57.67	38.6	–23.38	–24.94
recruit_weather	1.85	–0.03	–1.66	–3.68

**Parameter**	**Variation**			
	**–10%**	**–5%**	**5%**	**10%**
senescence_age	–3.22	–2.04	–0.4	0.4
senescence_increase	–0.82	–0.27	–1.15	–1.58
mort_min	–1.75	–0.93	–0.56	–1.46
mort_a	2.69	1.91	–2.28	–3.51
mort_b	–7.71	–4.35	3.63	8.58
mort_c	13.52	6.21	–6.14	–11.68
mort_d	5.97	2.44	–3.58	–5.82
mort_e	–8.88	–5.11	4.15	9.3
mort_f	12.96	4.71	–6.02	–11.27
cone_cycle	2.9	1.66	–2.83	–2.12
seed_longevity	–2.26	–0.42	–0.99	–0.85
flower_age_a	–3.59	–1.87	1.04	2.18
flower_age_b	1.91	0.31	–0.99	–4.54
flower_age_c	–1.86	–0.35	–1.44	–0.34
flower_weather_a	9.55	3.79	–3.28	–6.79
flower_weather_b	–5.23	–2.36	1.53	3.54
flower_weather_c	–10.56	–5.89	3.16	9.11
flower_weather_d	–22.88	–14	12.68	26.61
flower_weather_e	32.69	15.72	–16.18	–27.68
pollination	–3.64	–2.03	0.47	1.8

**Parameter**	**Variation**			
	**–10%**	**–5%**	**5%**	**10%**
follicles	–3.09	–2.5	–0.15	2.63
seeds	–3.77	–3.02	1.1	2.29
firm_seeds	–2.75	–1.16	1.2	1.82
viable_seeds	–2.18	–2.81	0.61	1.07
insect_a	0.31	–1.15	–1.07	–1.29
insect_b	–0.39	–0.3	–1.19	–0.52
decay_a	–0.08	–0.65	–1.73	–1.43
decay_b	–0.36	–0.91	–1.24	–0.79
open_a	0.35	–1.5	–0.92	–0.71
open_b	–0.49	–0.31	–0.32	0.05
open_c	–0.53	–0.53	–1.52	–0.54

8. Model output corroboration

This TRACE element provides supporting information on: How model predictions compare to independent data and patterns that were not used, and preferably not even known, while the model was developed, parameterized, and verified. By documenting model output corroboration, model users learn about evidence, which, in addition to model output verification, indicates that the model is structurally realistic so that its predictions can be trusted to some degree.

Summary:

We would need more data to compare the results of our model with independent data. We compared the baseline (i.e., reference) scenario with the current scenario. Most of the model parameters were based on older, published references to the baseline climate scenario for two main reasons: i) new data were not available or were insufficient, and ii) the inclusion of more differences between the climate scenarios would make it more complex to understand the results of the simulations for interpretation and to answer the research questions set out in our study. The parameters of the current climate scenario that differ from the reference scenario were the weather impacts on flower production (flower count data), fertile cones, and the number of follicles per cone (i.e., fruit set).

Literature cited

Breshears, D. D., Fontaine, J. B., Ruthrof, K. X., Field, J. P., Feng, X., Burger, J. R., Law, D. J., Kala, J., & Hardy, G. E. St. J. (2021). Underappreciated plant vulnerabilities to heat waves. *New Phytologist*, *231*(1), 32–39. https://doi.org/10.1111/nph.17348

Bullock, J. M., Wichmann, M. C., Hails, R. S., Hodgson, D. J., Alexander, M. J., Morley, K., Knopp, T., Ridding, L. E., & Hooftman, D. A. P. (2020). Human-mediated dispersal and disturbance shape the metapopulation dynamics of a long-lived herb. *Ecology*, *101*(8), e03087. https://doi.org/10.1002/ecy.3087

Delignette-Muller, M. L., & Dutang, C. (2015). fitdistrplus: An R Package for Fitting Distributions. *Journal of Statistical Software*, *64*(4), 1–34. https://doi.org/10.18637/jss.v064.i04

Dornier, A., Pons, V., & Cheptou, P.-O. (2011). Colonization and extinction dynamics of an annual plant metapopulation in an urban environment. *Oikos*, *120*(8), 1240–1246. https://doi.org/10.1111/j.1600-0706.2010.18959.x

Enright, N. J., Fontaine, J. B., Bowman, D. M., Bradstock, R. A., & Williams, R. J. (2015). Interval squeeze: Altered fire regimes and demographic responses interact to threaten woody species persistence as climate changes. *Frontiers in Ecology and the Environment*, *13*(5), 265–272. https://doi.org/10.1890/140231

Enright, N. J., Keith, D. A., Clarke, M. F., & Miller, B. P. (2012). Fire regimes in Australian sclerophyllous shrubby ecosystems: Heathlands, heathy woodlands and mallee woodlands. *Flammable Australia: Fire Regimes and Biodiversity in a Changing World. Melbourne: CSIRO Publishing*, 215–235.

Enright, N. J., & Lamont, B. B. (1989). Seed banks, fire season, safe sites and seedling recruitment in five co-occurring *Banksia* species. *The Journal of Ecology*, *77*(4), 1111. https://doi.org/10.2307/2260826

Enright, N. J., Lamont, B. B., & Marsula, R. (1996). Canopy seed bank dynamics and optimum fire regime for the highly serotinous shrub, *Banksia hookeriana*. *The Journal of Ecology*, *84*(1), 9. https://doi.org/10.2307/2261695

Enright, N. J., Marsula, R., Lamont, B. B., & Wissel, C. (1998). The ecological significance of canopy seed storage in fire-prone environments: A model for resprouting shrubs. *Journal of Ecology*, *86*(6), 960–973. https://doi.org/10.1046/j.1365-2745.1998.00311.x

Esther, A., Groeneveld, J., Enright, N. J., Miller, B. P., Lamont, B. B., Perry, G. L. W., Schurr, F. M., & Jeltsch, F. (2008). Assessing the importance of seed immigration on coexistence of plant functional types in a species-rich ecosystem. *Ecological Modelling*, *213*(3), 402–416. https://doi.org/10.1016/j.ecolmodel.2008.01.014

Flannigan, M. D., Krawchuk, M. A., Groot, W. J. de, Wotton, B. M., & Gowman, L. M. (2009). Implications of changing climate for global wildland fire. *International Journal of Wildland Fire*, *18*(5), 483–507. https://doi.org/10.1071/WF08187

Glasa, J., & Halada, L. (2008). On elliptical model for forest fire spread modeling and simulation. *Mathematics and Computers in Simulation*, *78*(1), 76–88. https://doi.org/10.1016/j.matcom.2007.06.001

Grainger, S., Fawcett, R., Trewin, B., Jones, D., Braganza, K., Jovanovic, B., Martin, D., Smalley, R., & Webb, V. (2022). Estimating the uncertainty of Australian area-average temperature anomalies. *International Journal of Climatology*, *42*(5), 2815–2834. https://doi.org/10.1002/joc.7392

Green, D. G. (1983). Shapes of simulated fires in discrete fuels. *Ecological Modelling*, *20*(1), 21–32. https://doi.org/10.1016/0304-3800(83)90029-7

Grimm, V., Berger, U., Bastiansen, F., Eliassen, S., Ginot, V., Giske, J., Goss-Custard, J., Grand, T., Heinz, S. K., Huse, G., Huth, A., Jepsen, J. U., Jørgensen, C., Mooij, W. M., Müller, B., Pe'er, G., Piou, C., Railsback, S. F., Robbins, A. M., … DeAngelis, D. L. (2006). A standard protocol for describing individual-based and agent-based models. *Ecological Modelling*, *198*(1), 115–126. https://doi.org/10.1016/j.ecolmodel.2006.04.023

Grimm, V., Berger, U., DeAngelis, D. L., Polhill, J. G., Giske, J., & Railsback, S. F. (2010). The ODD protocol: A review and first update. *Ecological Modelling*, *221*(23), 2760–2768. https://doi.org/10.1016/j.ecolmodel.2010.08.019

Grimm, V., Railsback, S. F., Vincenot, C. E., Berger, U., Gallagher, C., DeAngelis, D. L., Edmonds, B., Ge, J., Giske, J., Groeneveld, J., Johnston, A. S. A., Milles, A., Nabe-Nielsen, J., Polhill, J. G., Radchuk, V., Rohwäder, M.-S., Stillman, R. A., Thiele, J. C., & Ayllón, D. (2020). The ODD protocol for describing agent-based and other simulation models: A second update to improve clarity, replication, and structural realism. *Journal of Artificial Societies and Social Simulation*, *23*(2), 7. https://doi.org/10.18564/jasss.4259

Groeneveld, J., Enright, N. J., & Lamont, B. B. (2008). Simulating the effects of different spatio-temporal fire regimes on plant metapopulation persistence in a Mediterranean-type region. *Journal of Applied Ecology*, *45*(5), 1477–1485. https://doi.org/10.1111/j.1365-2664.2008.01539.x

Groeneveld, J., Enright, N. J., Lamont, B. B., & Wissel, C. (2002). A spatial model of coexistence among three *Banksia* species along a topographic gradient in fire-prone shrublands. *Journal of Ecology*, *90*(5), 762–774. https://doi.org/10.1046/j.1365-2745.2002.00712.x

Hanski, I. (1998). Metapopulation dynamics. *Nature*, *396*(6706), Article 6706. https://doi.org/10.1038/23876

Hantson, S., Scheffer, M., Pueyo, S., Xu, C., Lasslop, G., Van Nes, E. H., Holmgren, M., & Mendelsohn, J. (2017). Rare, intense, big fires dominate the global tropics under drier conditions. *Scientific Reports*, *7*(1). Scopus. https://doi.org/10.1038/s41598-017-14654-9

He, T., Krauss, S. L., Lamont, B. B., Miller, B. P., & Enright, N. J. (2004). Long-distance seed dispersal in a metapopulation of Banksia hookeriana inferred from a population allocation analysis of amplified fragment length polymorphism data. *Molecular Ecology*, *13*(5), 1099–1109. https://doi.org/10.1111/j.1365-294X.2004.02120.x

He, T., Lamont, B. B., Krauss, S. L., & Enright, N. J. (2010). Genetic connectivity and inter-population seed dispersal of Banksia hookeriana at the landscape scale. *Annals of Botany*, *106*(3), 457–466. https://doi.org/10.1093/aob/mcq140

Henzler, J., Weise, H., Enright, N. J., Zander, S., & Tietjen, B. (2018). A squeeze in the suitable fire interval: Simulating the persistence of fire-killed plants in a Mediterranean-type ecosystem under drier conditions. *Ecological Modelling*, *389*, 41–49. https://doi.org/10.1016/j.ecolmodel.2018.10.010

Johnston, T. R., Stock, W. D., & Mawson, P. R. (2016). Foraging by Carnaby's Black-Cockatoo in Banksia woodland on the Swan Coastal Plain, Western Australia. *Emu - Austral Ornithology*, *116*(3), 284–293. https://doi.org/10.1071/MU15080

Kassambara, A. (2020). *ggpubr: "ggplot2" Based Publication Ready Plots*. https://CRAN.R-project.org/package=ggpubr

Keith, D. A., Lindenmayer, D., Lowe, A., Russell-Smith, J., Barrett, S., Enright, N. J., Fox, B. J., Guerin, G., Paton, D. C., Tozer, M. G., & others. (2014). 7 Heathlands. *Biodiversity and Environmental Change: Monitoring, Challenges and Direction*, 213.

Lozano, O. M., Salis, M., Ager, A. A., Arca, B., Alcasena, F. J., Monteiro, A. T., Finney, M. A., Giudice, L. D., Scoccimarro, E., & Spano, D. (2017). Assessing climate change impacts on wildfire exposure in Mediterranean areas. *Risk Analysis*, *37*(10), 1898–1916. https://doi.org/10.1111/risa.12739

Matsumoto, M., & Nishimura, T. (1998). Mersenne Twister: A 623-dimensionally Equidistributed Uniform Pseudo-random Number Generator. *ACM Trans. Model. Comput. Simul.*, *8*(1), 3–30. https://doi.org/10.1145/272991.272995

Mouillot, F., Rambal, S., & Joffre, R. (2002). Simulating climate change impacts on fire frequency and vegetation dynamics in a Mediterranean-type ecosystem. *Global Change Biology*, *8*(5), 423–437. https://doi.org/10.1046/j.1365-2486.2002.00494.x

Nolan, R. H., Boer, M. M., Collins, L., Resco de Dios, V., Clarke, H., Jenkins, M., Kenny, B., & Bradstock, R. A. (2020). Causes and consequences of eastern Australia's 2019–20 season of mega-fires. *Global Change Biology*, gcb.14987. https://doi.org/10.1111/gcb.14987

Railsback, S. F., & Grimm, V. (2019). *Agent-Based and Individual-Based Modeling: A Practical Introduction, Second Edition*. Princeton University Press.

Sievert, C. (2020). *Interactive Web-Based Data Visualization with R, plotly, and shiny*. Chapman and Hall/CRC. https://plotly-r.com

Silva, S. S. D., Fearnside, P. M., Graça, P. M. L. D. A., Brown, I. F., Alencar, A., & Melo, A. W. F. D. (2018). Dynamics of forest fires in the southwestern Amazon. *Forest Ecology and Management*, *424*, 312–322. Scopus. https://doi.org/10.1016/j.foreco.2018.04.041

Slowikowski, K. (2021). *ggrepel: Automatically Position Non-Overlapping Text Labels with "ggplot2."* https://CRAN.R-project.org/package=ggrepel

Souto-Veiga, R., Groeneveld, J., Enright, N. J., Fontaine, J. B., & Jeltsch, F. (2022). Declining pollination success reinforces negative climate and fire change impacts in a serotinous, fire-killed plant. *Plant Ecology*, *223*(7), 863–881. https://doi.org/10.1007/s11258-022-01244-7

Timbal, B., Arblaster, J. M., & Power, S. (2006). Attribution of the Late-Twentieth-Century Rainfall Decline in Southwest Australia. *Journal of Climate*, *19*(10), 2046–2062. https://doi.org/10.1175/JCLI3817.1

Ushey, K., Allaire, J. J., & Tang, Y. (2022). *reticulate: Interface to "Python."* https://CRAN.R-project.org/package=reticulate

Valente, F., & Laurini, M. (2021). Spatio-temporal analysis of fire occurrence in Australia. *Stochastic Environmental Research and Risk Assessment*, *35*(9), 1759–1770. https://doi.org/10.1007/s00477-021-02043-8

Wickham, H. (2011). The Split-Apply-Combine Strategy for Data Analysis. *Journal of Statistical Software*, *40*(1), 1–29.

Wickham, H. (2016). *ggplot2: Elegant Graphics for Data Analysis*. Springer-Verlag New York. https://ggplot2.tidyverse.org

Wickham, H., François, R., Henry, L., & Müller, K. (2022). *dplyr: A Grammar of Data Manipulation*. https://CRAN.R-project.org/package=dplyr

Witkowski, E. T. F., Lamont, B. B., & Obbens, F. J. (1994). Commercial Picking of Banksia hookeriana in the Wild Reduces Subsequent Shoot, Flower and Seed Production. *The Journal of Applied Ecology*, *31*(3), 508. https://doi.org/10.2307/2404446

Zadeh, L. A. (1988). Fuzzy logic. *Computer*, *21*(4), 83–93. https://doi.org/10.1109/2.53
