# Supplementary figures and images for: Climate change may shift metapopulations towards unstable source‐sink dynamics in a fire‐killed, serotinous shrub
Source: Ecol Evol. 2024 Jun 3;14(6):e11488. doi: 10.1002/ece3.11488 (PMC11148395; doi:10.1002/ece3.11488)

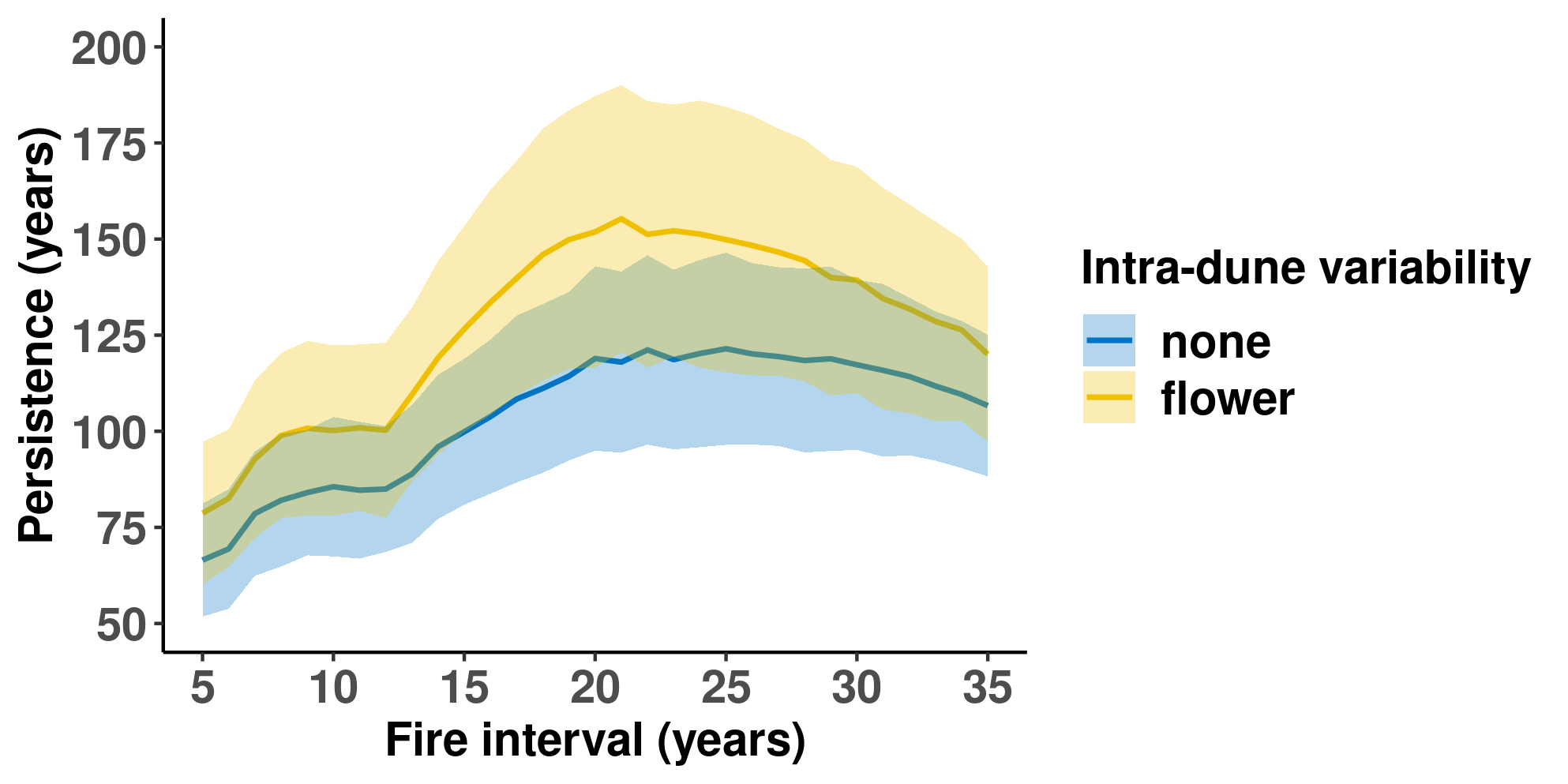

Supplement: Supplementary file 1 — Data S1 [file ECE3-14-e11488-s001.zip › ece311488-sup-0001-SupinfoS1/figure_A10.tiff]

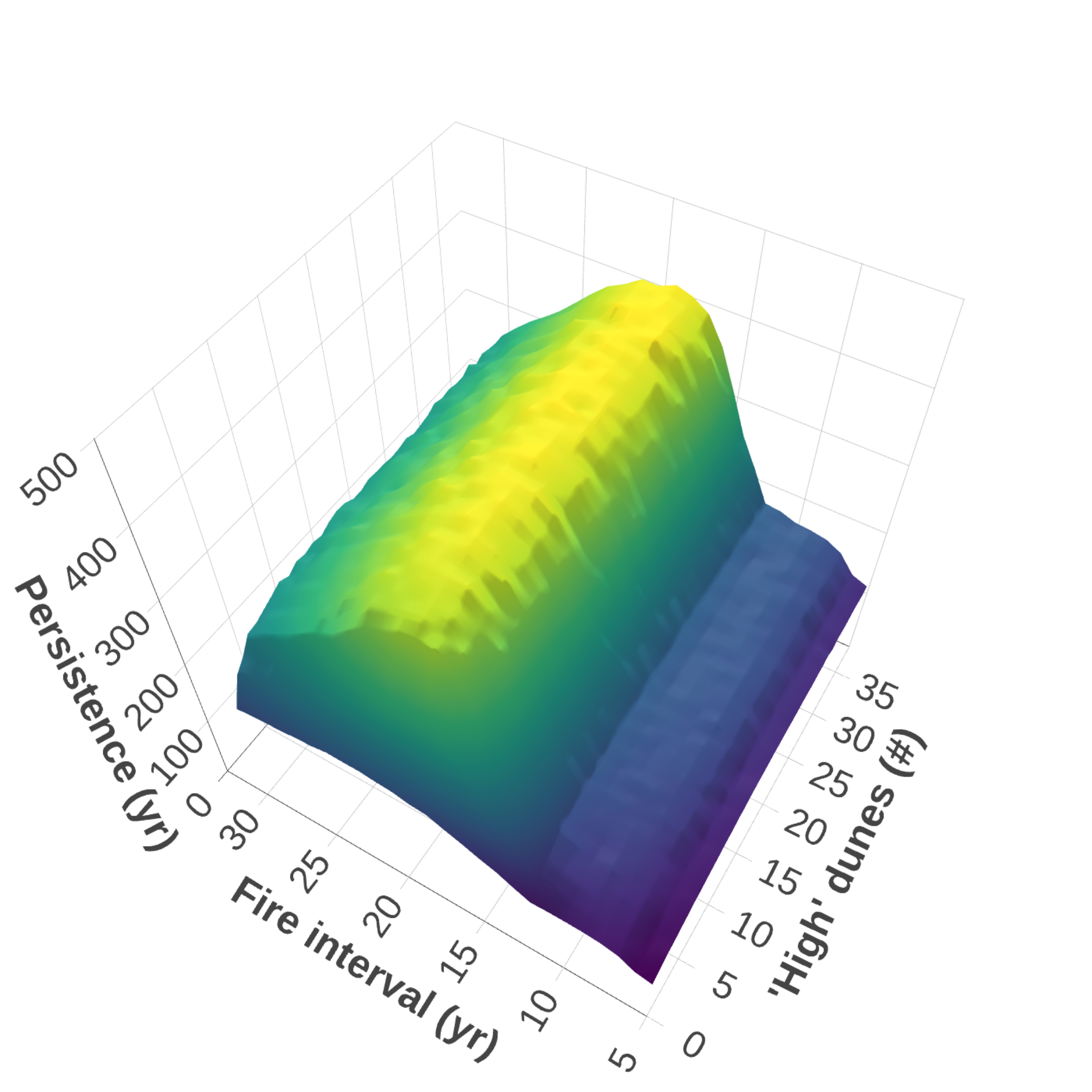

Supplement: Supplementary file 1 — Data S1 [file ECE3-14-e11488-s001.zip › ece311488-sup-0001-SupinfoS1/figure_A11.tiff]

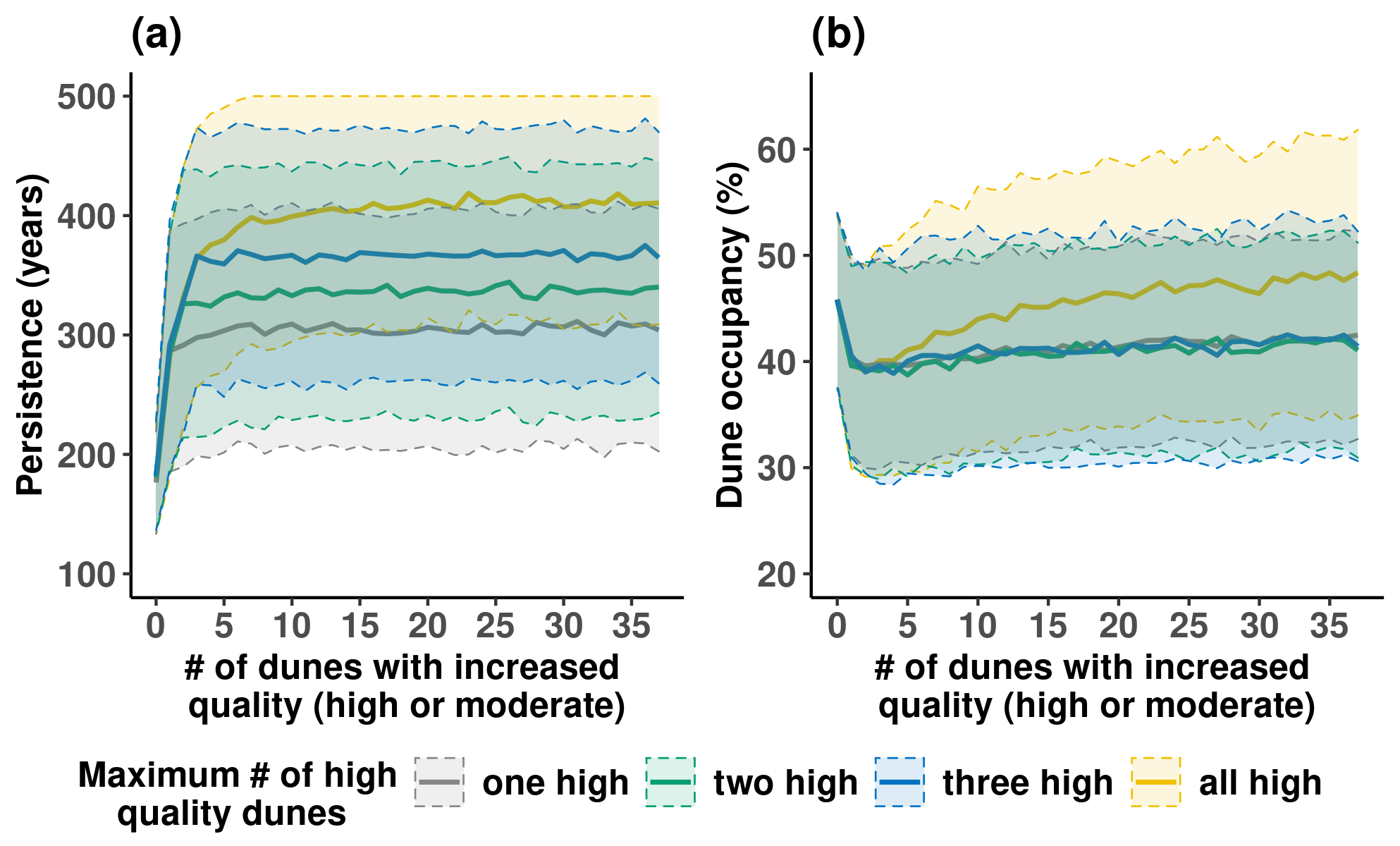

Supplement: Supplementary file 1 — Data S1 [file ECE3-14-e11488-s001.zip › ece311488-sup-0001-SupinfoS1/figure_A12.tiff]

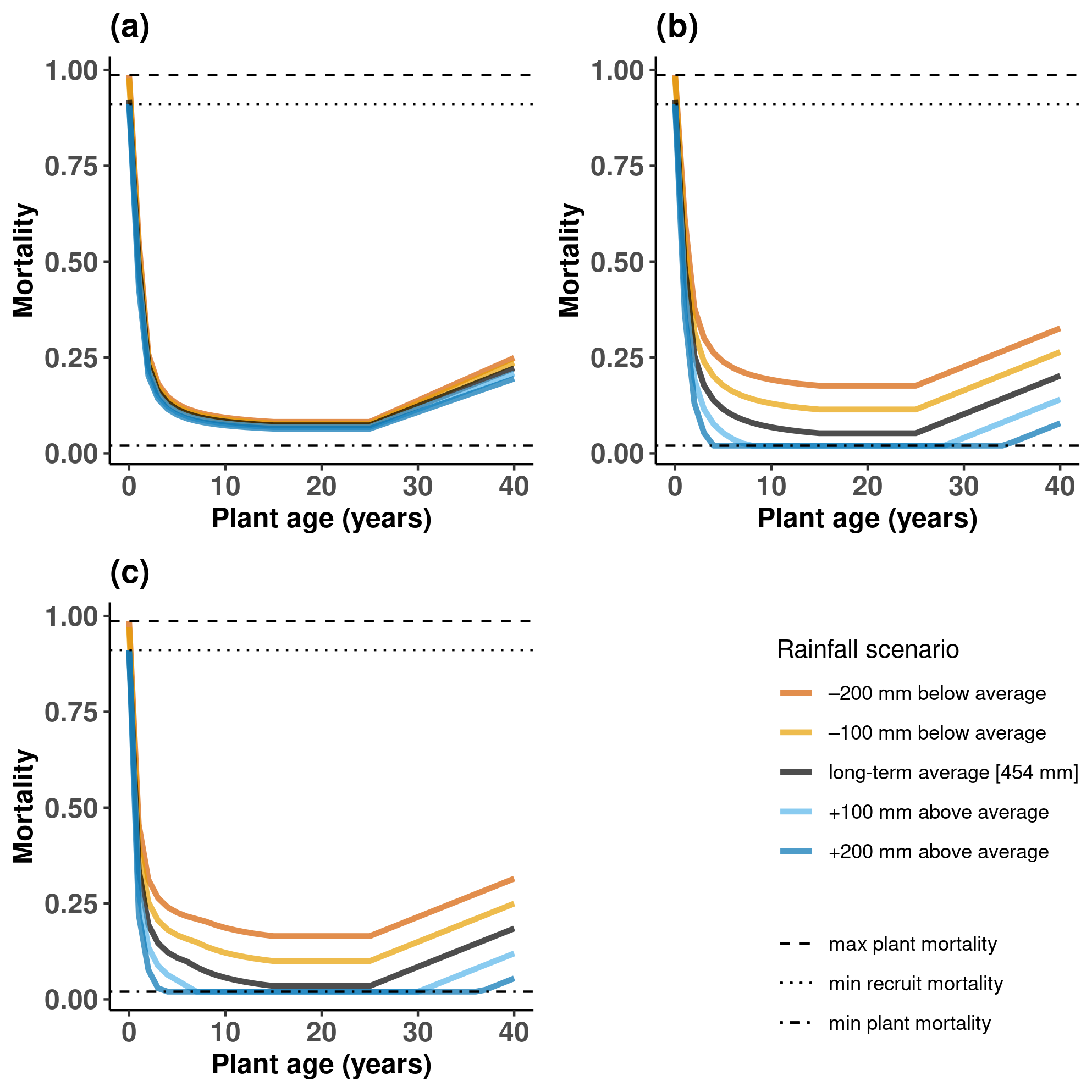

Supplement: Supplementary file 1 — Data S1 [file ECE3-14-e11488-s001.zip › ece311488-sup-0001-SupinfoS1/figure_A2.tiff]

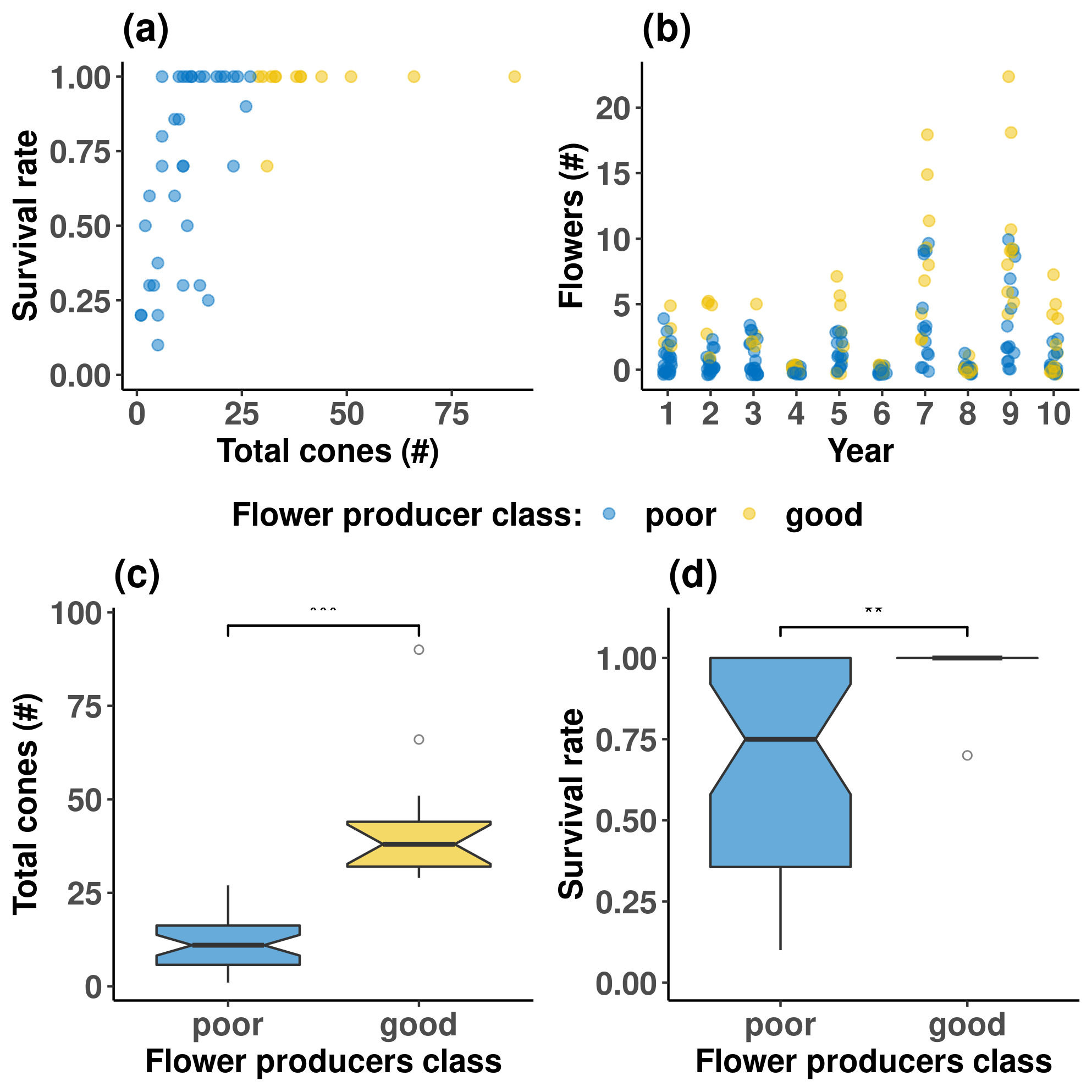

Supplement: Supplementary file 1 — Data S1 [file ECE3-14-e11488-s001.zip › ece311488-sup-0001-SupinfoS1/figure_A3.tiff]

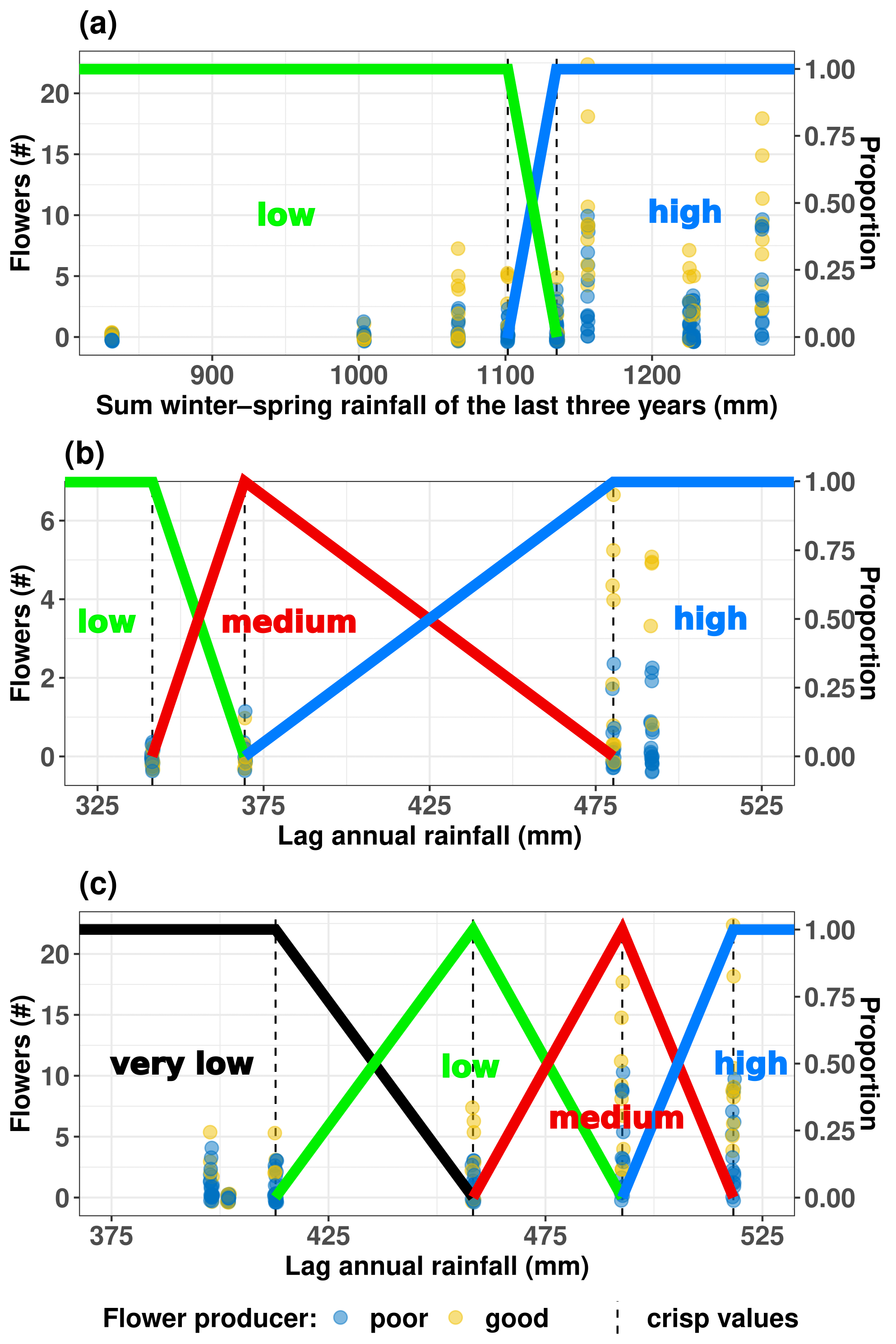

Supplement: Supplementary file 1 — Data S1 [file ECE3-14-e11488-s001.zip › ece311488-sup-0001-SupinfoS1/figure_A4.tiff]

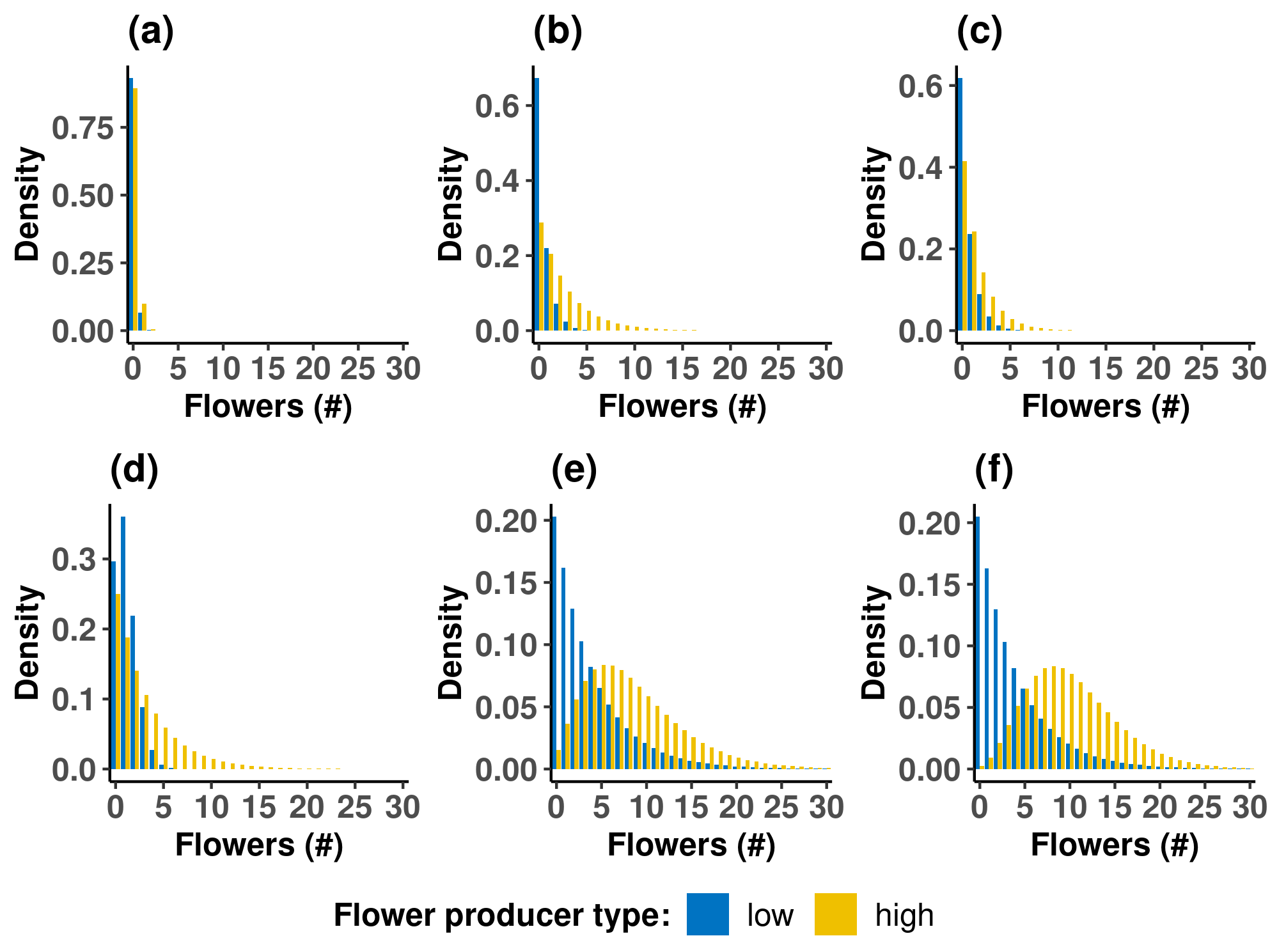

Supplement: Supplementary file 1 — Data S1 [file ECE3-14-e11488-s001.zip › ece311488-sup-0001-SupinfoS1/figure_A5.tiff]

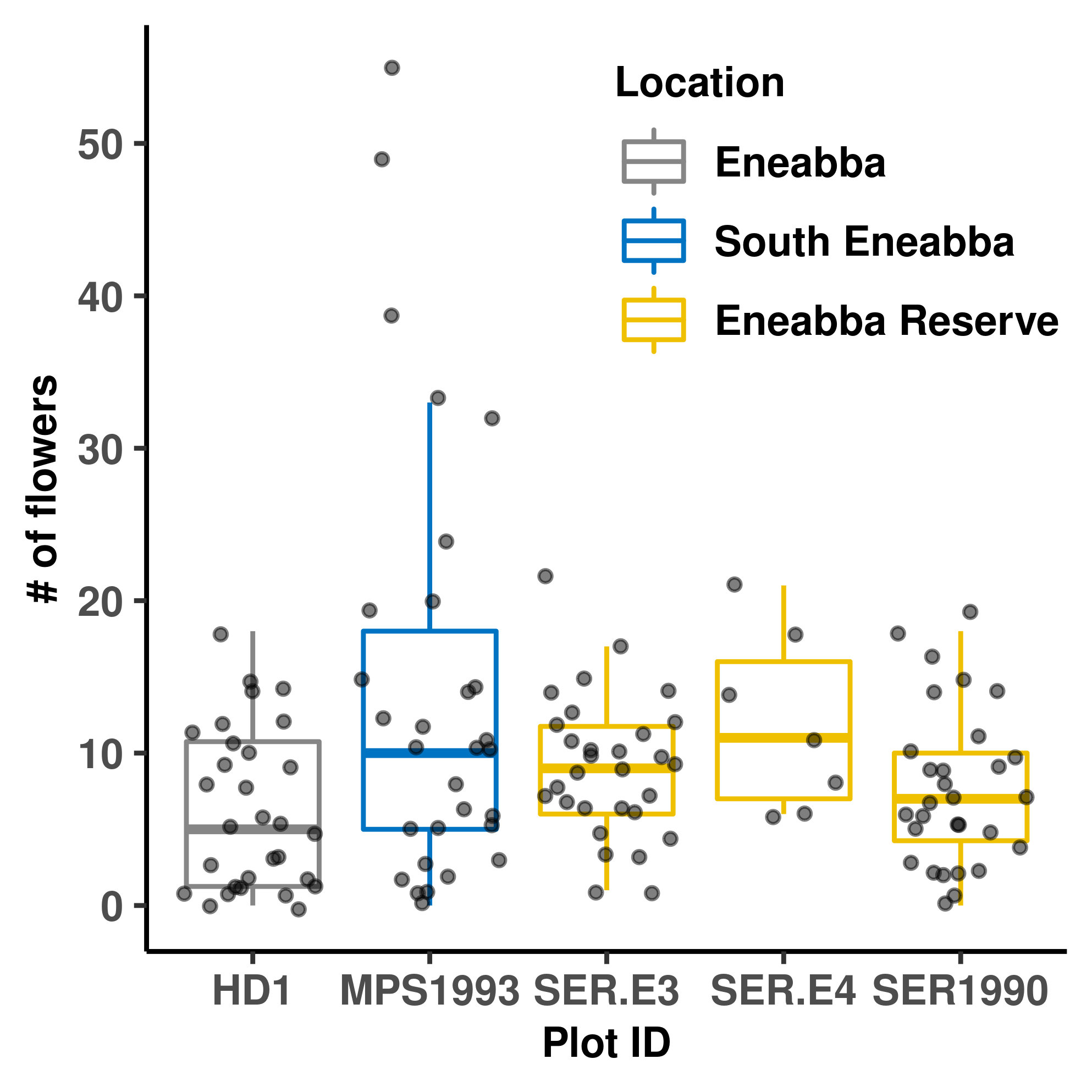

Supplement: Supplementary file 1 — Data S1 [file ECE3-14-e11488-s001.zip › ece311488-sup-0001-SupinfoS1/figure_A6.tiff]

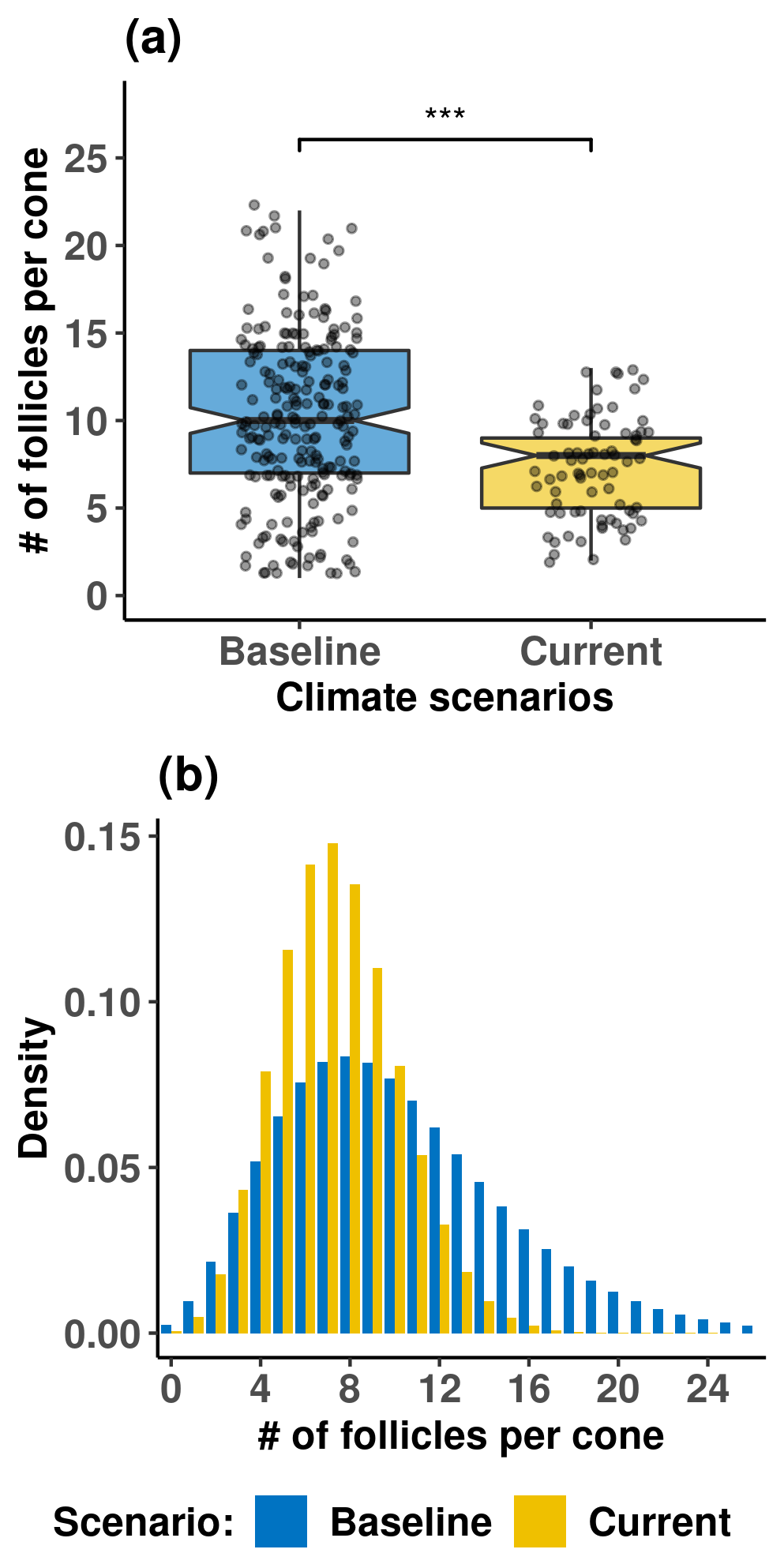

Supplement: Supplementary file 1 — Data S1 [file ECE3-14-e11488-s001.zip › ece311488-sup-0001-SupinfoS1/figure_A7.tiff]

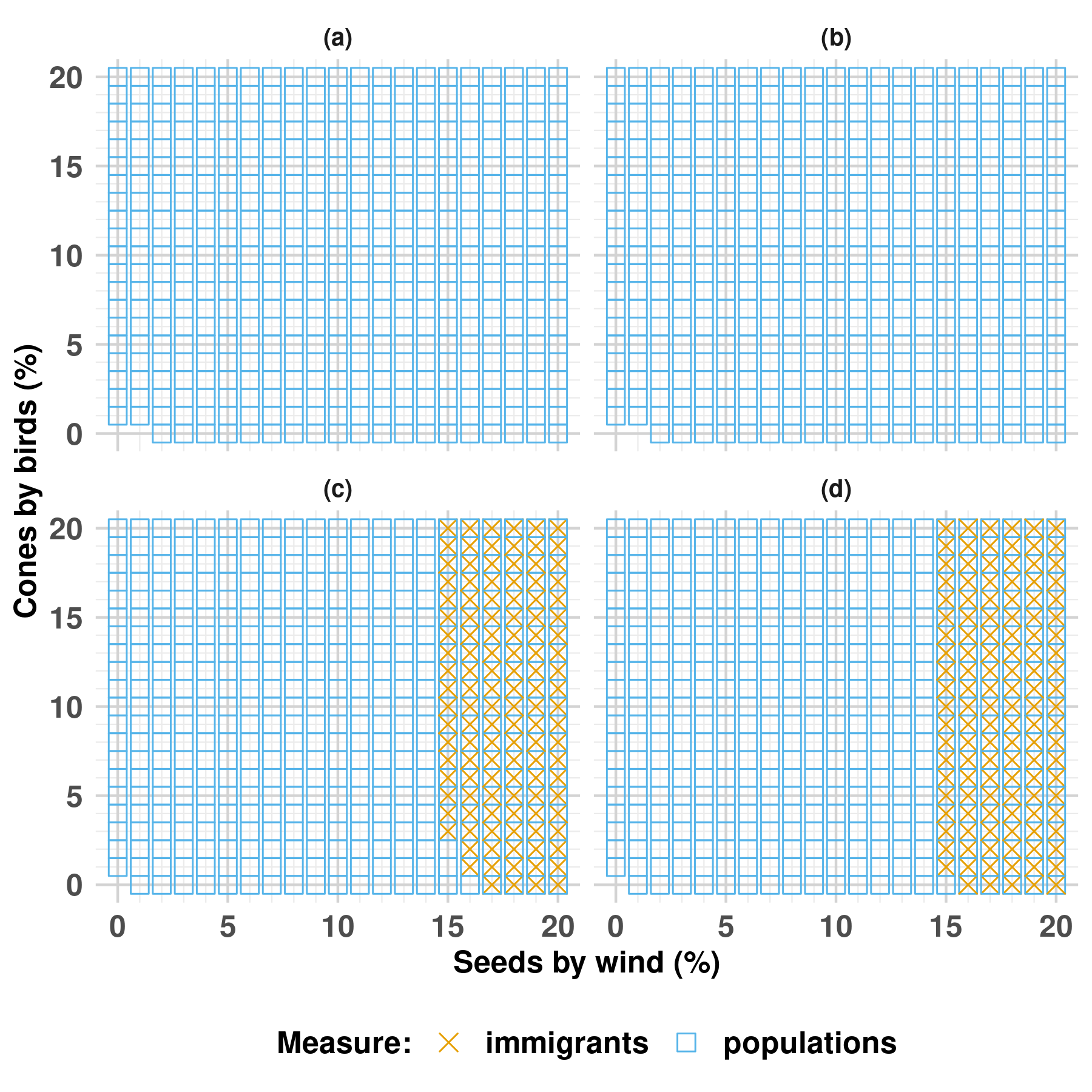

Supplement: Supplementary file 1 — Data S1 [file ECE3-14-e11488-s001.zip › ece311488-sup-0001-SupinfoS1/figure_A8.tiff]

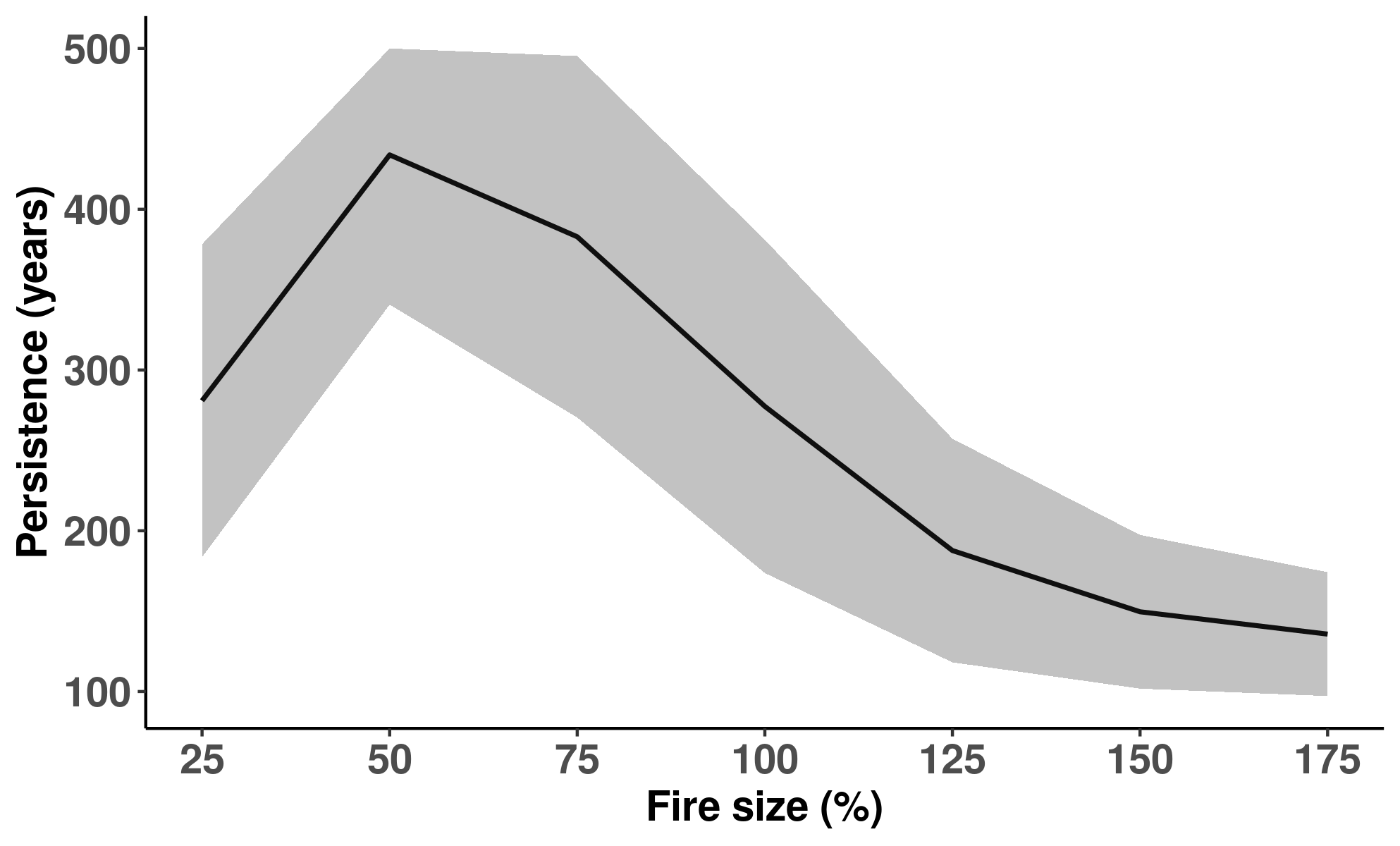

Supplement: Supplementary file 1 — Data S1 [file ECE3-14-e11488-s001.zip › ece311488-sup-0001-SupinfoS1/figure_A9.tiff]
